# Convergence and divergence in gesture repertoires as an adaptive mechanism for social bonding in primates

**DOI:** 10.1098/rsos.170181

**Published:** 2017-11-29

**Authors:** Anna Ilona Roberts, Sam George Bradley Roberts

**Affiliations:** 1 Department of Psychology, University of Chester, Parkgate Road, Chester CH1 4BJ, UK; 2 School of Natural Sciences and Psychology, Liverpool John Moores University, Byrom Street, Liverpool L3 3AF, UK

**Keywords:** chimpanzees, gestural repertoire, homogeneity, heterogeneity, social network analysis, generalized linear model

## Abstract

A key challenge for primates living in large, stable social groups is managing social relationships. Chimpanzee gestures may act as a time-efficient social bonding mechanism, and the presence (homogeneity) and absence (heterogeneity) of overlap in repertoires in particular may play an important role in social bonding. However, how homogeneity and heterogeneity in the gestural repertoire of primates relate to social interaction is poorly understood. We used social network analysis and generalized linear mixed modelling to examine this question in wild chimpanzees. The repertoire size of both homogeneous and heterogeneous visual, tactile and auditory gestures was associated with the duration of time spent in social bonding behaviour, centrality in the social bonding network and demography. The audience size of partners who displayed similar or different characteristics to the signaller (e.g. same or opposite age or sex category) also influenced the use of homogeneous and heterogeneous gestures. Homogeneous and heterogeneous gestures were differentially associated with the presence of emotional reactions in response to the gesture and the presence of a change in the recipient's behaviour. Homogeneity and heterogeneity of gestural communication play a key role in maintaining a differentiated set of strong and weak social relationships in complex, multilevel societies.

## 1. Introduction

One of the most intriguing questions in the science of human origins involves the definition of language, its fundamental function and the selective pressures that were responsible for its evolution [1–6]. Viewed as a system of cognition and communication, one key selection pressure for the evolution of language may have been to facilitate social bonding and group cohesion in increasingly large groups of hominins [7,8]. The extent to which language can act as a social bonding mechanism may be affected by the degree of overlap in the repertoire between communication partners (homogeneity) [9]. For instance, a series of studies has shown that language and accent are one of the fundamental categories by which people categorize others into groups [10–14]. Even young, five- to six-month-old infants show a preference for people speaking in their native language, compared to foreign speakers [10]. Further, because language and accent are inseparable from the person and difficult to falsify, they may have been an important social marker through the course of human evolution, facilitating the development of ‘tag-based’ cooperation across increasingly large and dispersed social groups [9,11,14,15]. Language does not fossilize, so examining how repertoire homogeneity in non-human primates relates to social bonding can provide insights into the importance of overlap in communication repertoire in facilitating both dyadic interactions and large-scale sociality through the course of hominin evolution [15].

Until recently, the question of language evolution and sociality has received an almost exclusive research focus on the vocal modality of non-human primates, because the principal medium of human language is vocal [16]. However, the vocal and gestural components of language operate in a complementary fashion and human communication depends strongly also on visual cues. Gestural communication is defined as voluntary movements of the arms, head, body postures and locomotory gaits [17–26]. It has been theorized that language in humans evolved primarily in the gestural modality, because primates use gestures intentionally and have a greater control over their limbs than their vocal output. Moreover, the acquisition of new vocal signals in primate species other than humans is debated, whereas acquisition of gestures has been claimed in many studies [7,18,27–36]. However, the arguments in favour of the gestural origins of language are mainly based on findings in captive apes and cannot be extrapolated to wild populations because there are different selective pressures involved in captive compared to wild apes. For instance, the communicative behaviour of captive apes may be biased by frequent contact with humans in early ontogeny [37,38].

Wild East African chimpanzees (*Pan troglodytes schweinfurthii*) provide a valuable opportunity to assess the link between the social bonding and the homogeneity of gestural communication of primates. Chimpanzees form socially and geographically circumscribed communities, within which they associate in temporary subgroups (parties) that vary in size, composition and duration [39]. The community size can be in the range of 20–150 individuals, and the community as a whole is rarely seen together in one place [39]. Chimpanzees are frugivores and communities defend a communal home range, which is typically in the range of 5–35 km^2^. Individuals in the wider community are thus often temporarily and spatially separated, but maintain long-term relationships through repeated and reciprocated interactions in many contexts [40–42].

One important mechanism to maintain social relationships in chimpanzees is grooming behaviour—grooming releases endorphins, enabling strong social bonds to be developed [43]. As well as grooming interactions between kin, grooming creates familiarity between unrelated group members originating in the knowledge of past relationships with the social partner that forms the basis for trust and social bond formation. In smaller networks, primates can form strong bonds with all network members such as kin and unrelated individuals and maintain relationships with them through grooming behaviour. However, the increasing time and cognitive demands on managing social relationships in networks containing many conspecifics imply that some network members become unfamiliar and ties become progressively weaker [44]. Thus in large social groups, there are dyads with less frequent interaction and the network contains an increasing number of individuals who cannot rely on grooming to maintain social relationships [45]. The ability to maintain network cohesion in large social groups depends on behaviours that enable primates to develop a social bond with individuals in the absence of grooming behaviour.

The social bond between group members could develop on the basis of phenotypic similarity such as shared facial characteristics [46]. The tendency to interact with others who have similar phenotypic characteristics is driven by fitness benefits of cooperating with kin [47,48]. In this case, phenotypic similarity enables the signaller to develop trust and bond with the dyad partner. However, when there are individuals in the group that share limited phenotypic similarity, the ability to establish social bonds with unrelated individuals may limit the capacity to maintain large social networks. When a lack of phenotypic similarity limits the ability to bond with unfamiliar or unrelated group members, individuals can use external cues such as communication similarity to create a tolerant context in the absence of prior relationships or genetic relatedness.

Similarity in communication can be established by increasing overlap in gestural expressions of the signaller's affect that release neurohormones associated with social bonding in the recipients [43]. These expressions are communicative to the recipients about the affect evoked by the social relationship and therefore influence the strength of social bonding between dyad partners. Moreover, similarity can occur in gestures used in a goal-directed way, whereby the signaller has a goal and uses a gesture that refers to the role of the recipient in attaining the signaller's goal, by indicating to the recipient through the gesture what they have to do. The gestures are responded to by goal-directed activity that matches the goal of the signaller, enabling more efficient social bonding between dyad partners [49,50].

Similarity in communication can arise by pruning down a set of innate gestures into a shared subset [26,51–53]. Such pruning occurs in repeated interactions with dyad partners during which individuals identify which gestures are effective. For instance, iterated learning experiments with humans have demonstrated that convergence of communication arises out of repeated interactions[54–60].

However, socially driven acquisition of gestures could be beneficial to both signallers and the recipients. When individuals who preferentially interact also preferentially learn from each other [61,62], gesture acquisition results in bonded dyads and groups with an increasingly large repertoire of homogeneous communication [63]. This homogenization may increase as the network of relationships individual primates have to manage increases. The patterns of homogenization may be differentiated by modalities of gestures such as visual (received through looking), tactile (received through touch) and auditory (received through hearing), as these modalities are differentially suited to maintaining different types of social bonds [64]. Visual or tactile gestures are most effective in one-to-one interactions, coordinating behaviour and acting as a time-efficient social bonding mechanism [45]. However, one-to-one gestures require a high degree of close proximity between the signaller and receiver to be detected in a dense forest habitat. Thus, there is a limit to which primates can keep increasing similarity of their gestures in one-to-one gestural interactions as the network size increases.

The need to manage the differentiated social network of strong and weak ties may lead to homogenization of communication that can be used to manage relationships between unfamiliar and unrelated individuals [9,65,66]. This type of communication may enable social bonding by prompting release of social neurohormones, which helps to create social bonds in the absence of prior grooming behaviour. In the absence of frequent grooming, this type of communication can increase the degree of similarity perceived between unfamiliar or unrelated interactants through some external cue that approximates phenotypic similarity that can be acquired without a need to be involved in direct one-to-one relationships with the signaller. A particular case of gesture acquisition in which the external evaluation of communication may be crucial for both learning and social bonding is object use. For instance, chimpanzees use objects to make loud sounds that can be perceived by both the immediate audience and the out-of-sight audience, enabling individuals to learn social signals and bond ‘at a distance’ [45]. Auditory gestures such as drumming have a higher amplitude, meaning they can evoke stronger emotions that may be better suited as a larger scale bonding mechanism [45,64,67,68]. Individuals with a greater degree of homogeneity in their communicative repertoires of visual, tactile and auditory gestures may therefore coordinate differentiated relationships better, when compared with individuals with lower levels of homogeneity [45,60,64,69,70].

Patterns of overlap in gestural communication can be influenced by the demography of the surrounding audience. In addition to the role of overlap in gestures in chimpanzee sociality, one aspect of primate social relationships that has not been considered in relation to the efficacy of social bonding is the absence of overlap in the repertoire of gestural communication (heterogeneity). For instance, avian studies postulated that heterogeneous communication can draw attention to the signaller when the immediate audiences are large, and this role is not necessarily restricted to avian species [71]. When phenotypic similarity with the immediate audience is high, signalling dissimilarity through heterogeneous gestures can increase the salience of the signaller to the recipient. Standing out in the crowd of similar others can enable signallers to gain a competitive advantage over individuals similarly suited to the social bonding with the recipient. By definition, heterogeneous gestures signal dissimilarity from the members of the social group or membership of a different social group independently of the phenotype characteristics, but the role of heterogeneous signalling in regulating social dynamics has so far been neglected. Very few studies to date have explored the potentially important role of heterogeneous communication in the coordination of social behaviour in primates.

Hence, it could be predicted that homogeneity and heterogeneity in the gestural repertoire would be useful in effectively managing social relationships with conspecifics, as reflected in the relationship between features of the gestural repertoire and social interaction. The relationship between homogeneity of communication and social dynamics has previously been described in the vocal domain of the chimpanzees. Studies have examined acoustic similarities in the panthoot calls within the group (panthoot recipients joining in the panthoot matching the acoustic structure of the initiator) [72] and group-level acoustic differences in panthoots [73]. In the gestural domain, studies have reported intra- and intergroup differences in the grooming hand-clasp gesture [74–78] and homogeneity of attention-getting auditory gestures across mother–offspring chimpanzee dyads [79,80]. However, research has not systematically examined how overlap in the whole gesture repertoire relates to sociality, either in captive or wild species [81].

This study examines how the homogeneity of gestural repertoire (defined as the degree of overlap in the presence or absence of gesture types in the repertoire between pairs of individuals, i.e. the number of shared gesture types) and heterogeneity of gestural repertoire (number of unshared gesture types) are related to social bonds of wild chimpanzees, measured by the duration of social bonding behaviours (joint feeding, joint resting, joint travelling, grooming, visual attention and in proximity) per hour pairs of chimpanzees spend in the same party. Moreover, we examine how the immediate audience (number of the individuals of similar age or sex present within 10 m) influences the use of these gestures. In this study, we predict that homogeneity and heterogeneity in the gestural repertoire of chimpanzees are not distributed randomly across dyads, but can be explained by biological factors (maternal kinship, age similarity, sex similarity), social relationships (duration of time spent in social bonding behaviour—when within 2 m, per hour spent in the same party), audience size (e.g. number of opposite-sex partners) social network size (centrality) and characteristics of gesture modality [61,62]. We examined the influence of these factors on dyadic and group-level homogeneity and heterogeneity in the gestural repertoire in the Sonso group of wild East African chimpanzees (*Pan troglodytes schweinfurthii*) in Budongo Forest, Uganda, Africa.

## 2. Material and methods

### 2.1. Data collection protocol

The data collection and methods for this study were approved by the Budongo Conservation Field Station research committee, the Uganda National Council for Science and Technology (NS 124) and the Uganda Wildlife Authority (UWA/TBDP/RES/50). In this study, observations of 12 habituated East African chimpanzees (*Pan troglodytes schweinfurthii*) (six adult males and six adult females) of the Sonso community, the Budongo Conservation Field Station, Budongo Forest Reserve in Uganda (www.budongo.org) were conducted in September 2006, between April and July 2007 and March and June 2008. The focal subjects were chosen on the basis of lack of any limb injuries and to represent age and rank classes as equally as possible. All of the females selected as focal subjects were parous. Full details of the study site and subjects have been described previously [82], so only brief details are given here. The behaviour of the chimpanzees was recorded during a standardized observation period using focal animal follows. The subjects were chosen systematically and, to avoid dependency in the dataset, consecutive samples of the same focal subject were taken at least 20 min apart. The data for this study came from the following sources. First, 18-min focal follows were performed. These consisted of nine scans at 2 min intervals of the identity of individuals present within 10 m of the focal subject and more than 10 m away from the focal individual who were in the same party. The party was defined as the group of individuals within a spread of about 35 m. Second, the identity of the adult nearest neighbour of the focal individual, the distance between the focal individual and the nearest neighbour (in metres), and the presence or absence of visual attention between the focal individual and the nearest neighbour were recorded. The visual attention was scored on the basis of bodily orientation rather than precise gaze direction between dyad partners. The activity state of both the focal subject and the nearest neighbour were also recorded (e.g. grooming, feeding, travel). Third, gestures were continuously recorded using a digital video camera recorder and this was accompanied by a verbal description of context, i.e. the identity of the signaller and the recipient, their behaviour prior to and after production of the gesture, and goal directedness. The presence of any calls accompanying the gestures was also noted. The sampling of association patterns was conducted by an experienced field assistant who was unaware of the aims of the study. The field assistants undergo an inter-observer reliability test annually, an interval which is sufficient to maintain the consistency of scoring of the group composition and proximity across field assistants, with results consistently above 0.85 for Spearman's rank correlation coefficient, *r*_s_. The video recording of the gestures was carried out by A.R. and thus the social data and gestural data were collected independently of each other. Full details of the methods of data collection have been described previously [17,83,84].

### 2.2. Video analyses of gestural communication

The video footage was viewed on a television and coded. The initial catalogue of non-verbal behaviours was scored as an act of gestural communication if it was an expressive movement of the limbs or head and body posture that was: (i) mechanically ineffective (a gesture always elicited a change in the recipient's behaviour by non-mechanical means); (ii) communicative (i.e. at the level of the gesture type, communication was consistently associated with a change in the behaviour of the recipient after the signal), thus gestures occurred in social circumstances and only social circumstances when gestures were used were included in the dataset; for instance, if a chimpanzee gestured for non-social means (e.g. turn the back to change position rather than turn the back to initiate grooming), such circumstances of gesturing would not be considered [17]; and (iii) intentional. We used criteria for intentionality scoring that were previously developed to define intentionality in human infants and have been widely used in primates [85,86]. These include: (i) the presence of an audience; (ii) response waiting (the signaller directs a gesture at a recipient and observes the recipient's response during and after the gesture); (iii) the production of a gesture is sensitive to the recipient's visual attention state; and (iv) the signaller persists in gesture production when the recipient fails to respond. These intentionality criteria were evaluated for each gesture type separately, using pooled data across all subjects. Gestures above the threshold of 60% of cases were classified as intentional.

Full details of how gestures were categorized, including video clips of each gesture type, have been provided previously [17,84]. Briefly, visual, manual gesture types were established on the basis of 29 distinct morphological features, such as trajectory and orientation, using hierarchical cluster and discriminant function analysis [84]. Other gesture types were established qualitatively on the basis of objective judgement of the similarity of morphology of gestures (i.e. presence/absence and type of head, trunk, arm movement; posture; social orientation) [17]. This latter type of procedure has been widely used to identify distinct gesture types in chimpanzees [18,25,26,87] and other primates [51,52,88–90].

In line with previous definitions using this database of gestures [17], a ‘gesture sequence’ was defined as one or more gestures made consecutively by one individual towards the same recipient, within the same context, within a maximum of 30 s interval. There were a total of 545 gesture sequences included in the analysis. These gesture sequences included both single gestures and series of gestures, and in all sequences one of the 12 focal chimpanzees was the signaller. For each gesture sequence, we recorded the identity of the signaller (the individual performing a gesture), the identity of the recipient (the individual at whom the gesture was most clearly directed, as determined from the orientation of head and body of the signaller during or immediately after performing a gesture, i.e. the signaller had the recipient within its field of view), the recipient's behaviour after production of the gesture (response), the signaller's behaviour prior to and after production of the gesture, and the eliciting stimuli if present (e.g. presence of an intra-party dispute).

Gestures were classified according to the sensory modality (visual, tactile, short-range auditory, long-range auditory) [64]. The coding for the context and modality of a gesture was validated by a second coder, who scored a random sample of 10.42% of the gesture sequences examined in this paper. Cohen's κ coefficient showed that reliability was excellent for the modality of gesturing (*K* = 0.946) [91]. In addition, another sample of 50 sequences of gestures was coded by a second coder for intentionality (response waiting and persistence) and the Cohen's κ coefficient showed good reliability (*K* = 0.74). Reliability of coding into gesture types has been described in a previous publication [84], with the Cohen's Kappa coefficient showing good reliability (*K* = 0.76).

### 2.3. Behavioural measures

To reduce pseudoreplication, we aimed to sample each focal subject when the party had a unique composition, i.e. there was a change in composition of either focal males or females from the time the last subsample of the preceding focal follow was taken. The proximity (examined here for independence of 10 m associations only) and grooming scans were taken 2 min apart during the 18 min sample duration. The samples of each consecutive focal subject were taken at least 20 min apart. To ensure that this sampling procedure did not bias our results, we tested for similarity in association patterns between scans taken at 2 (scan 1), 4 (scan 2) and 18 min (scan 9) of the focal sample, including both sexes. There was no significant difference in the number of times focal and non-focal subjects were in close proximity at scan 1 (median = 2, IQ range = 0–5) and scan 2 (median = 2, IQ range = 1–5, Wilcoxon's signed-ranks test, *T* = 411.50, *N* = 132, *p* = 0.435). However, there was a significant difference in the number of times focal and non-focal subjects were in close proximity at scan 1 and scan 9 (median = 2, IQ range = 1–4; Wilcoxon's signed-ranks test, *T* = 2656.50, *N* = 132, *p* = 0.011). Similarly, there was no significant difference in the number of times focal and non-focal subjects were in the same party at scan 1 (median = 5, IQ range: 3–10) and scan 2 (median = 5, IQ range: 3–10; Wilcoxon's signed-ranks test, *T* = 218.50, *N* = 132, *p* = 0.571). However, there was a significant difference in the number of times focal and non-focal subjects were in the same party at scan 1 and scan 9 (median = 5, IQ range: 2–10; Wilcoxon's signed-ranks test, *T* = 1460, *N* = 132, *p* = 0.010). Thus, the adjacent scans were similar for 10 m associations and were treated as continuous. As 10 m associations and 2 m associations were correlated, we made an assumption of independence for both of these measures. Moreover, party-level associations were also treated as continuous. However, first and final sample scans differed for 10 m associations and party-level associations and therefore we treated these scans as independent, as well as the samples preceding and succeeding the focal follow. Behavioural measures were then derived, calculating the duration of time each pair of chimpanzees spent engaged in affiliative behaviours, per hour that pair of chimpanzees spent in the same party. These affiliative behaviours were: joint feeding, joint resting, joint travelling, giving grooming, receiving grooming, mutual grooming, visual attention towards the focal, visual attention away from the focal and proximity. An example of how these measures were computed can be found in the electronic supplementary material, S1 and a more detailed description has been provided previously [45].

### 2.4. Attribute measures

To control for the influence of demography, factors such as age, kinship, sex and reproductive state need to be taken into account when examining chimpanzees' propensity to associate with each other. Genetic data obtained in previous studies provided the basis for ascertaining kin relationships in the Sonso community and we scored chimpanzee dyads according to the presence or absence of kinship [82]. In the wild, chimpanzees reach physical and social maturity in the age range of 15–16 years [39]. The Sonso community is a long-running site and therefore the age of most adult subjects in the community is known. We classified dyads of chimpanzees as belonging to the same (5 years or less of age difference) or a different (above 5 years of age difference) age class [92]. Moreover, chimpanzee dyads were scored according to oestrous similarity. The reproductive status of the female was scored on the basis of the female sexual swelling, which is an enlarged area of the perineal skin varying in size over the course of the menstrual cycle. The reproductive status of the female was recorded as oestrous if, during the observation period, the female exhibited maximum tumescence (sexual swelling) and was observed mating with the males. The size of the sexual swelling was rated on a scale of 1–4, with the maximum swelling size scored as 4.

All focal males were observed to mate with the females and, therefore, assumed to be reproductively active. Dyads were classified as reproductively active (males and oestrous females), or non-reproductively active (all other combinations). Sex similarity was scored based on observable morphological characteristics referring to sex. Full details of the categorization of attribute data can be found in the electronic supplementary material, table S1.

### 2.5. Gestural repertoire homogeneity

For each gesture sequence, homogeneity was determined for each gesture type used. If the gesture type (e.g. arm beckon) was present in the repertoires of both the signaller and the receiver, this was classified as homogeneous. By contrast, if the arm beckon appeared in the repertoire of the signaller but not the recipient, it would be classified as heterogeneous. As gesture sequences can sometimes consist of a series of individual gestures, each gesture type used was classified separately as homogeneous or heterogeneous. Thus for a gesture sequence consisting of a series of five different gesture types, three gesture types could be classified as homogeneous and two as heterogeneous. For each gesture sequence, the ‘homogeneous repertoire size’ refers to the number of different homogeneous gesture types used by the signaller, and the ‘heterogeneous repertoire size’ refers to the number of different heterogeneous gesture types used. If only homogeneous repertoire size was taken into account, a repertoire size of three homogeneous gestures could represent a gesture sequence of five gestures, with three homogeneous gesture types and two heterogeneous gesture types, or a sequence of three gestures, all of which were homogeneous. Thus, we considered both homogeneous and heterogeneous repertoire size in our analyses, to identify sequences where heterogeneous gesture types were used.

For the network analysis, the overall number of gesture types shared (homogeneous) and not shared (heterogeneous) between dyad partners was included in the analyses rather than gesture rates. Because we provided the overall number of shared and non-shared gestures in this instance, for heterogeneous gestures the total number of non-shared gestures was given. For instance, if BB had 5 gesture types that were absent in HW repertoire and HW had two gesture types that were absent in BB repertoire, the heterogeneous repertoire size for both the BB HW dyad and the HW BB dyad was 7.

To determine the degree of homogeneity in the gestural repertoires between focal chimpanzees, using social network analysis, we used Cohen's κ coefficient [93]. This statistic measures the degree of agreement in the presence or absence of gestures in the repertoires of dyad partners. If the repertoire of gestures of a focal individual is in complete agreement with the repertoire of gestures of the dyad partner, then the Cohen's κ coefficient equals 1 (exactly the same repertoire) for that dyad. However, if the repertoire of gestures is in complete disagreement with the repertoire of gestures of the dyad partner, then the Cohen's κ coefficient equals −1 (completely different repertoire). In this study, the repertoire of gestures of each focal subject that were performed towards other adult individuals was compared within dyads (electronic supplementary material, table S2) [17]. In addition to all gestures combined, the Cohen's κ coefficient for each dyad was computed separately for visual, tactile, auditory short-range and auditory long-range gestures. In these analyses, 10 focal subjects (90 dyads) contributed data—subjects KU and ZM were excluded as they did not gesture in all modalities towards other adults. In previous research comparing repertoires within and between primate groups, Cohen's κ has been the most commonly used statistical technique, based on the presence or absence of gesture types from the repertoire [17,18,52]. The data pertaining to this part of this study has been placed in the electronic supplementary material, S2.

### 2.6. Generalized linear mixed modelling

The panthoot call is a type of chimpanzee vocalization which is broadcast at a wider audience. When performing social network analysis, we took all of the individuals who were within 10 m of the signaller as recipients of the gestures accompanied by panthoot call. Generalized linear mixed modelling (GLMM) prohibits this action, however, because all of the observations have to be independent from each other. In this case, all sequences of gestures that contained solely visual gestures and panthoots (low-intensity panthoot) were counted as directed by the signaller at the most dominant individual in the party. By contrast, all sequences of gestures containing panthoots and auditory gestures were assumed to be directed at a nearest neighbour of the focal individual (high-intensity panthoot) [45]. The data used in this study consisted of 545 gesture sequences produced by the 12 focal chimpanzees. In line with previous work based on this dataset [94], we used GLMM to examine how homogeneity and heterogeneity in gestural communication was related to the bonding behaviours. GLMM is a modified form of regression analysis designed to deal with data that are hierarchically structured. The random effects in all models were the identity of the focal individual and we included random intercepts for these effects. Random slopes were not used in these models as the key focus was on how the predictor variables relating to homogeneity were associated with the different bonding behaviours, rather than how the effects of these differed between the 12 focal subjects. In these GLMMs, the data were hierarchically structured with two levels—level 1 was the focal individual and level 2 was the recipient of the gesture. The response variables in the GLMMs were continuous (repertoire size, duration of social behaviour) or binary: the presence or absence of a gesture, demography (e.g. sex difference).

The models were fitted using a binomial error structure with logit link. In all of the analyses, the demographic relationships (e.g. age similarity), bonding status (e.g. duration of joint travel) and the presence or absence of response were controlled for. However, when demography was a response variable, the analyses only considered the presence and absence of a gesture. When analysing the relationship between homogeneous and heterogeneous gesture presence (the response variable) and the response type to the gesture, only single, unimodal gestures were considered because a previous study showed that including combinations of gestures was likely to influence the type of response made to the gesture [95]. In all analyses where audience size was included, the audience size excluded the signaller and the recipient of the gesture. Moreover, only one category of audience was included in each analysis (e.g. size of audience of same-age and different-age partners was included separately from size of audience of same-sex and opposite-sex partners). The descriptive statistics regarding variables included in the GLMMs are provided in table 1. The Generalized Linear Mixed Models function in IBM SPSS Statistics 22 was used for all the GLMM models. The data used in all GLMM models can be found in the electronic supplementary material, S3–S8.

**Table 1.** Variables included in generalized linear mixed models. Owing to missing data, the total number of cases differs between variables. The missing values are denoted as 999 in all datasets. All durations are in minutes.

predictor variable	definition	mean ± s.d. or presence/absence
sex difference	sex difference between the focal subject and the recipient (0 = opposite sex, 1 = same sex)	0 = 227, 1 = 318
age difference	age difference between the focal subject and the recipient (0 = different age, 1 = same age)	0 = 378, 1 = 167
oestrous difference	oestrous relationship between the focal subject and the recipient: 0 = reproductively inactive (unoestrous female–unoestrous female, unoestrous female–oestrous female, oestrous female–oestrus female, unoestrous female–male, male–male), 1 = reproductively active (male–oestrous female)	0 = 391, 1 = 122
maternal kinship	maternal kinship presence between the focal subject and the recipient (0 = non-kin, 1 = kin)	0 = 530, 1 = 14
joint feeding	duration of joint feeding with the dyad partner when within 2 m and nearest neighbours per hour spent in the same party	1.26 ± 1.86
joint resting	duration of joint resting with the dyad partner when within 2 m and nearest neighbours per hour spent in the same party	1.90 ± 2.25
joint travel	duration of joint travelling with the dyad partner when within 2 m and nearest neighbours per hour spent in the same party	0.77 ± 1.58
grooming given	duration of grooming given to the dyad partner per hour spent in the same party	2.22 ± 2.76
grooming received	duration of grooming received from the dyad partner per hour spent in the same party	1.03 ± 1.98
grooming mutual	duration of mutually grooming with the dyad partner per hour spent in the same party	1.59 ± 3.67
attention present	duration of mutual bodily orientation presence with the dyad partner when within 2 m and nearest neighbours per hour spent in the same party	6.03 ± 7.86
attention absent	duration of mutual bodily orientation absence with the dyad partner when within 2 m and nearest neighbours per hour spent in the same party	3.81 ± 3.48
proximity	duration of proximity with the dyad partner when within 2 m and nearest neighbours per hour spent in the same party	9.79 ± 9.55
response absence or presence	presence of any change in the behaviour of the recipient following production of the gesture (0 = absent, 1 = present)	0 = 208, 1 = 258
goal-directed response or emotional display	change of behaviour by means of goal-directed response, whereby recipient performs some action that conforms to the goal of the signaller (e.g. starts to groom) or emotional display, which may include tactile, visual or vocal behaviour, produced by the recipient after the gesture which is not followed by goal-directed action that conforms to the goal of the signaller (e.g. embrace during travel, whereby signallers travel immediately before and after the embrace (0 = activity, 1 = emotional display)	0 = 166, 1 = 92
response by vocal display	change of behaviour by means of vocal display, which involves production of sound via vocal tract by the recipient, which is not followed by goal-directed action towards the signaller (e.g. pantgrunt during travel, whereby signallers travel before and after the pantgrunt (0 = absent, 1 = present)	0 = 400, 1 = 66
repertoire size of homogeneous gesture	total number of gesture types in a sequence which are present in both signaller's and recipient's repertoire of gestures present in the sequence	1.07 ± 0.82
repertoire size of heterogeneous gesture	total number of gesture types in a sequence produced by a signaller towards the recipient which are not present in the recipient's repertoire of gestures	0.48 ± 0.94
repertoire size of homogeneous visual gesture	total number of visual gesture types in a sequence (gestures that can only be received by looking at the signaller) which are present in both signaller's and recipient's repertoire	0.57 ± 0.71
repertoire size of homogeneous tactile gesture	total number of tactile gesture types in a sequence (gesture received by physical contact between the signaller and the recipient) which are present in both the signaller's and recipient's repertoire	0.10 ± 0.38
repertoire size of homogeneous auditory short-range gesture	total number of auditory short-range gesture types in a sequence (gesture can be received by hearing from a short distance without direct visual contact) which are present in both the signaller's and recipient's repertoire	0.24 ± 0.42
repertoire size of homogeneous auditory long-range gesture	total number of auditory long-range gesture types in a sequence (gesture can be received by hearing from a long distance without direct visual contact) which are present in the both signaller's and recipient's repertoire	0.15 ± 0.41
repertoire size of heterogeneous visual gesture	total number of visual gesture types in a sequence (gesture can only be received by looking at the signaller) which are not present in the recipient's repertoire of gestures	0.30 ± 0.62
repertoire size of heterogeneous tactile gesture	total number of tactile gesture types in a sequence (gesture received by physical contact between the signaller and the recipient) which are not present in the recipient's repertoire of gestures	0.07 ± 0.28
repertoire size of heterogeneous auditory short-range gesture	total number of auditory short-range gesture types in a sequence (gesture can be received by hearing from a short distance without direct visual contact) which are not present in the recipient's repertoire of gestures	0.01 ± 0.14
repertoire size of heterogeneous auditory long-range gesture	total number of auditory long-range gesture types in a sequence (gesture can be received by hearing from a long distance without direct visual contact) which are not present in the recipient's repertoire of gestures	0.08 ± 0.45
homogeneous gesture	sequence contains a gesture which is absent (0) or present (1) in the recipient's repertoire	0 = 76, 1 = 328
heterogeneous gesture	sequence contains a gesture which is absent in the recipient's repertoire: 0 = absent, 1 = present	0 = 274, 1 = 130
heterogeneous or homogeneous gesture	gesture types used in a sequence is either present in both signaller's and recipient's repertoire of gestures (1) or absent (0)	0 = 76, 1 = 274
presence or absence of homogeneous visual gesture	presence (1) or absence (0) of homogeneous visual gesture types in a sequence (gesture can be received by hearing from a long distance without direct visual contact)	0 = 215, 1 = 189
presence or absence of homogeneous tactile gesture	presence (1) or absence (0) of homogeneous tactile gesture types in a sequence (gesture can be received by hearing from a long distance without direct visual contact)	0 = 370, 1 = 34
presence or absence of homogeneous auditory short-range gesture	presence (1) or absence (0) of homogeneous auditory short-range gesture types in a sequence (gesture can be received by hearing from a long distance without direct visual contact)	0 = 306, 1 = 98
presence or absence of homogeneous auditory long-range gesture	presence (1) or absence (0) of homogeneous auditory long-range gesture types in a sequence (gesture can be received by hearing from a long distance without direct visual contact)	0 = 351, 1 = 53
presence or absence of heterogeneous visual gesture	presence (1) or absence (0) of heterogeneous visual gesture types in a sequence (gesture can be received by hearing from a long distance without direct visual contact)	0 = 307, 1 = 97
presence or absence of heterogeneous tactile gesture	presence (1) or absence (0) of heterogeneous tactile gesture types in a sequence (gesture can be received by hearing from a long distance without direct visual contact)	0 = 378, 1 = 26
presence or absence of heterogeneous auditory short-range gesture	presence (1) or absence (0) of heterogeneous auditory short-range gesture types in a sequence (gesture can be received by hearing from a long distance without direct visual contact)	0 = 398, 1 = 6
presence or absence of heterogeneous auditory long-range gesture	presence (1) or absence (0) of heterogeneous auditory long-range gesture types in a sequence (gesture can be received by hearing from a long distance without direct visual contact)	0 = 385, 1 = 19
audience same age as focal	number of same-age partners in the audience within 10 m from the focal subject	0.32 ± 0.02
audience different age than focal	number of different-age partners in the audience within 10 m from the focal subject	0.65 ± 0.04
audience same sex as focal	number of same-sex partners in the audience within 10 m from the focal subject	0.57 ± 0.04
audience opposite sex from focal	number of opposite-sex partners in the audience within 10 m from the focal subject	0.40 ± 0.03

### 2.7. Social network analysis

To complement the GLMM analysis, we used social network analysis to examine how individual variation in homogeneity for the focal chimpanzees was related to indegree and outdegree for bonding behaviours. Behavioural and communication networks were created for each behaviour type separately. Each network matrix consisted of 12 rows and 12 columns, with each row and column denoting a different focal chimpanzee. The values in each cell of the matrix represented the value for that particular behaviour for a specific pair of chimpanzees (e.g. the duration of time BB and HW spent in close proximity, per hour they spent in the same party). In the main analyses on factors influencing the proximity of dyads, the proximity and communication networks used in this study were weighted, i.e. each cell consisted of a continuous value representing the value of behaviour, rather than a 1 or a 0 indicating the presence or absence of a tie. In repertoire homogeneity networks, the value of Cohen's κ coefficient between dyads was entered in the matrices. The repertoire homogeneity networks were undirected. For instance, the overlap in the gestural repertoire between BB and HW was the same as the overlap between HW and BB. The proximity networks were treated as directed. For instance, the duration of time spent in close proximity by BB to HW may be different from the duration of time spent in close proximity by HW to BB.

From these network matrices, centrality measures were calculated, using normalized degree centrality [96]. Normalized degree centrality is defined as the average value of each row or column of the network matrix, i.e. the average value of that behaviour for each focal chimpanzee. The homogeneity network was undirected and therefore only the *n* degree value for each focal subject was obtained. This stands for the mean value of homogeneity of the gestural repertoire of each focal subject with all possible ties which are present. The behavioural network was different for the focal–non-focal subject dyads (e.g. BB to HW proximity was different from HW to BB proximity) and therefore indegree and outdegree were calculated separately. Outdegree refers to proximity directed by the focal chimpanzee to conspecifics, while indegree refers to proximity directed by conspecifics towards the focal chimpanzee.

General standard inferential statistics cannot be used on network data because the observations that make up network data are not independent of each other. Thus, randomization (or permutation) tests are used, whereby the observed value is compared against a distribution of values generated by a large number of random permutations of the data. The proportion of random permutations in which a value as large (or as small) as the one observed is then calculated, and this provides the *p*-value of the test [97]. MRQAP regression (multiple regression quadratic assignment procedure) was used to determine the relationships between social bonding networks and homogeneity of gestures [97]. MRQAP regression is similar to standard regression because it enables the examination of the effect of a number of predictor variables (e.g. visual and tactile homogeneity networks, sex similarity of a dyad) on an outcome variable (e.g. proximity network). Among several different types of MRQAP regression that are available, we used Double Dekker Semi-Partialling MRQAP regression, which is more robust against the effects of network autocorrelation and skewness in the data [98]. The number of permutations used in this analysis was 2000. For the node-level regressions, we used a similar procedure, using 10 000 random permutations to assess the effect of a number of predictor variables (e.g. the normalized mean degree for homogeneity of gestures, sex of focal chimpanzee) on the outcome variable (e.g. proximity in degree). All the social network analyses were carried out using UCINET 6 for Windows [99]. The data used in all social network analyses can be found in the electronic supplementary material, S2.

## 3. Results

The description of all variables included in the models is provided in table 1. The demographic details of the study group are provided in table 2. In all sections, only significant positive or negative associations between variables are reported. Further findings that elaborate on results presented in this section can be found in the electronic supplementary material.

**Table 2.** Demographic details of the study group.

focal subject ID	sex	year of birth	female reproductive status	pantgrunt given indegree	pantgrunt given outdegree
BB	male	1987 ± 1 year	—	0.937 (*β*)	0.134
HW	male	1993 ± 1 year	—	0.295	0.58
KT	male	1993	—	0	0.237
KU	female	∼1979	pregnant	0	0.231
KW	female	∼1981	nursing	0	0.609
ML	female	∼1975	cycling	0	0.407
MS	male	1991	—	0.313 (*γ*)	0.299
NB	female	∼1962	cycling	0	0.691
NK	male	1982 ± 1 year	—	2.224 (*α*)	0
RH	female	∼1965	nursing	0	0.21
SQ	male	1991 ± 1 year	—	0.258	0.268
ZM	female	∼1968	cycling	0	0.361

### 3.1. Social bonding behaviour and demography

We used GLMMs to examine the relationship between duration of time spent in social bonding behaviour and demographic characteristics of dyads across sequences of gestures. Table 3 presents a summary of this analysis. In terms of the influence of the demographic characteristics of the signaller and the recipient, chimpanzee dyads who were the same sex spent a longer duration of time mutually grooming (*β* = −1.854, s.e. = 0.732, *p* = 0.012), visually attending (*β* = −3.547, s.e. = 1.264, *p* = 0.005), visually non-attending (*β* = −1.220, s.e. = 0.433, *p* = 0.005) and in proximity (*β* = −4.825, s.e. = 1.320, *p* < 0.001). Moreover, the chimpanzee dyads who had were in the same age category spent a longer duration of time in joint travel (*β* = −1.914, s.e. = 0.963, *p* = 0.048), giving grooming (*β* = −2.469, s.e. = 0.763, *p* = 0.001), visually non-attending (*β* = −2.671, s.e. = 1.340, *p* = 0.047) and in proximity (*β* = −10.860, s.e. = 5.159, *p* = 0.036). Further, dyad partners with the same reproductive status showed a pattern of significantly longer duration of time spent in mutual grooming (*β* = −1.717, s.e. = 0.635, *p* = 0.007), visually attending (*β* = −3.020, s.e. = 1.039, *p* = 0.004) and in proximity (*β* = −2.684, s.e. = 1.250, *p* = 0.033). Finally, dyad partners related through maternal kinship spent a longer duration of time in the following social bonding behaviours: joint feeding (*β* = −1.831, s.e. = 0.198, *p* < 0.001), joint resting (*β* = −1.759, s.e. = 0.386, *p* < 0.001), giving grooming (*β* = −3.330, s.e. = 0.527, *p* < 0.001), receiving grooming (*β* = −1.755, s.e. = 0.563, *p* = 0.002), mutually grooming (*β* = −4.080, s.e. = 1.075, *p* < 0.001), visually attending (*β* = −10.646, s.e. = 2.025, *p* < 0.001), visually non-attending (*β* = −8.374, s.e. = 0.279, *p* < 0.001) and in proximity (*β* = −17.903, s.e. = 1.782, *p* < 0.001).

**Table 3.** Summary of results showing GLMM of the influence of demographic factors within sequences of gestures on the duration of bonding behaviours. Red squares indicate a negative value of *β*-coefficient. Blank squares indicate a relationship that was not statistically significant. If *β*-coefficient is positive, the category of zero in the predictor variable is associated with higher values of the dependent variable. If *β-*coefficient is negative, the category of zero in the predictor variable is associated with lower values of the dependent variable.

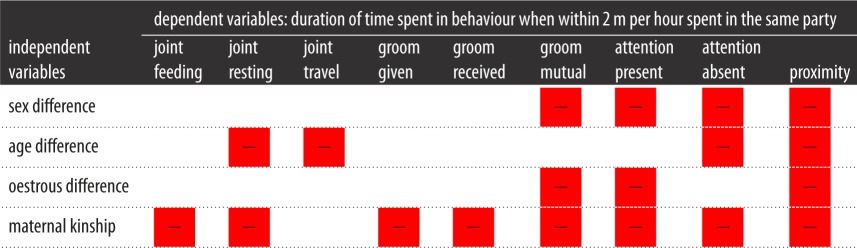

### 3.2. Homogeneous and heterogeneous gestures and demography

We used GLMMs to examine the relationship between demography and the presence or absence of homogeneous and heterogeneous gestures. Table 4 summarizes these findings. Overall, the presence of homogeneous gestures was more likely than the presence of heterogeneous gestures between individuals of the same age class (*β* = −2.517, s.e. = 0.761, *p* = 0.001), maternal kin (*β* = −0.919, s.e. = 0.386, *p* = 0.018) and reproductively inactive partners (*β* = 1.434, s.e. = 0.621, *p* = 0.022). Homogeneous visual gestures were produced between partners who were of the same age (*β* = −1.555, s.e. = 0.402, *p* < 0.001), same sex (*β* = −1.510, s.e. = 0.567, *p* = 0.008), maternal kin (*β* = −1.201, s.e. = 0.033, *p* < 0.001) and who were reproductively inactive (*β* = 2.263, s.e. = 0.651, *p* = 0.001). Homogeneous tactile gestures were used by maternal kin (*β* = −1.204, s.e. = 0.161, *p* < 0.001) and reproductively inactive partners (*β* = 3.889, s.e. = 0.723, *p* < 0.001). Homogeneous auditory short-range gestures were used by same age (*β* = −3.931, s.e. = 0.879, *p* < 0.001), same sex (*β* = −2.379, s.e. = 0.877, *p* = 0.007), maternal kin (*β* = −2.065, s.e. = 0.277, *p* < 0.001) and reproductively inactive dyads (*β* = 3.672, s.e. = 0.663, *p* < 0.001). Chimpanzee dyads who were of the same age category (*β* = −0.643, s.e. = 0.236, *p* = 0.007) but who were not maternal kin (*β* = 17.251, s.e. = 0.249, *p* < 0.001) used homogeneous auditory long-range gestures.

**Table 4.** Summary of results of GLMM of the relationship between the presence and absence of homogeneous and heterogeneous gestures within sequences and demographic factors. Green squares indicate a positive value of the *β*-coefficient; red squares indicate a negative value of the *β*-coefficient. Blank squares indicate a relationship that was not statistically significant. If the *β*-coefficient is positive, then the category of zero in the predictor variable is associated with the category of one in the dependent variable. If the *β*-coefficient is negative, then the category of zero in the predictor variable is associated with the category of zero in the dependent variable.

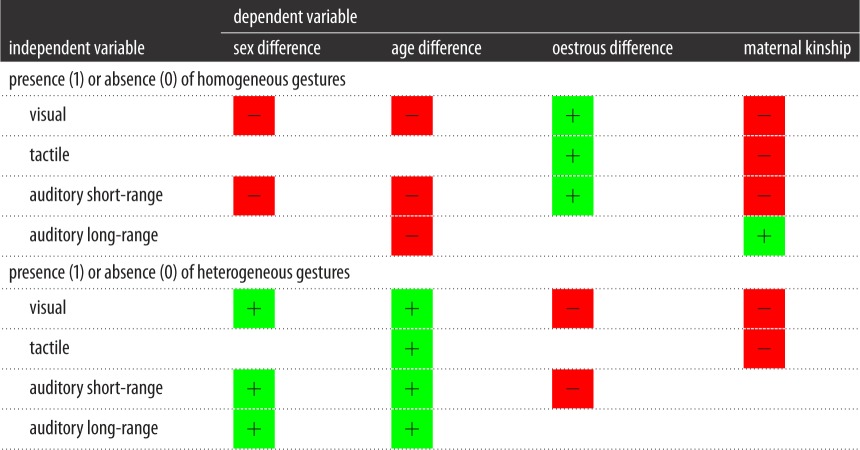

Heterogeneous visual gestures were present within dyads who were of opposite sex (*β* = 1.702, s.e. = 0.294, *p* < 0.001) and different age (*β* = 2.194, s.e. = 0.748, *p* = 0.004), and also who were reproductively active (*β* = −2.471, s.e. = 0.408, *p* < 0.001) and who were maternal kin (*β* = −0.566, s.e. = 0.159, *p* < 0.001). Different-age dyads (*β* = 1.980, s.e. = 0.417, *p* < 0.001) and dyads maternally related (*β* = −1.199, s.e. = 0.197, *p* < 0.001) were likely to use heterogeneous tactile gestures. Heterogeneous auditory short-range gestures were present between opposite sex (*β* = 11.174, s.e. = 0.069, *p* < 0.001), different age (*β* = 11.925, s.e. = 0.027, *p* < 0.001) and in reproductively active dyads (*β* = −4.072, s.e. = 1.055, *p* < 0.001). Opposite sex (*β* = 3.939, s.e. = 1.329, *p* = 0.003) and different age (*β* = 11.699, s.e. = 0.024, *p* < 0.001) characterized dyads who used heterogeneous auditory long-range gestures.

### 3.3. Homogeneity of gestures (Cohen's κ coefficient) and social bonding

We used multiple regression quadratic assignment procedures (MRQAP) to examine the relationship between homogeneity of gestures (Cohen's κ value) according to modality and the duration of time spent in bonding behaviour between dyads. Table 5 provides information on the mean value of ties of gesture networks. A greater degree of overlap in auditory short-range gestures was associated with a longer duration of: proximity (*r*^2^ = 0.316, *β* = 0.455, *p* = 0.003), travel (*r*^2^ = 0.391, *β* = −0.388, *p* = 0.010), feeding (*r*^2^ = 0.206, *β* = 0.290, *p* = 0.024), mutual grooming (*r*^2^ = 0.222, *β* = −0.451, *p* = 0.009), grooming received (*r*^2^ = 0.178, *β* = 0.366, *p* = 0.005), grooming given (*r*^2^ = 0.175, *β* = 0.310, *p* = 0.002), attention away (*r*^2^ = 0.211, *β* = 0.394, *p* = 0.010) and attention towards (*r*^2^ = 0.211, *β* = 0.527, *p* = 0.004). Greater homogeneity in tactile gestures was associated with a greater duration of grooming received (*r*^2^ = 0.178, *β* = 0.213, *p* = 0.008). By contrast, a greater degree of overlap in auditory long-range gestures was associated with a shorter duration of: proximity (*r*^2^ = 0.316, *β* = −0.364, *p* = 0.003), travel (*r*^2^ = 0.236, *β* = −0.388, *p* = 0.005), mutual grooming (*r*^2^ = 0.222, *β* = −0.363, *p* = 0.005) and attention towards the recipient (*r*^2^ = 0.211, *β* = −0.313, *p* = 0.007). Finally, greater homogeneity in visual gestures between pairs of chimpanzees was not significantly related to any of the bonding behaviours examined. Table 6 presents a summary of these results.

**Table 5.** Mean value of ties across dyadic gesture networks.

communication type	Cohen's κ coefficient^a^	dyadic repertoire size of homogeneous gestures	dyadic repertoire size of heterogeneous gestures
gestures overall	0.238	14.20	37.60
visual gesture	0.284	8.28	18.75
tactile gesture	0.167	2.96	10.03
auditory short-range gesture	0.259	0.50	1.92
auditory long-range gesture	0.201	2.45	6.89

^a^Cohen's κ coefficient ranges from −1 (completely different repertoire) to +1 (exactly the same repertoire).

**Table 6.** Summary of results MRQAP regression models predicting Cohen's κ, dyadic repertoire size of homogeneous gestures and dyadic repertoire size of heterogeneous gestures from duration of time spent in social bonding behaviour between dyads across four modalities of gestures: visual (V), tactile (T), auditory short-range (ASR) and auditory long-rage (ALR). Green squares indicate a positively significant relationship between the two variables; red squares indicate a negatively significant relationship. Blank squares indicate a relationship that was not statistically significant.

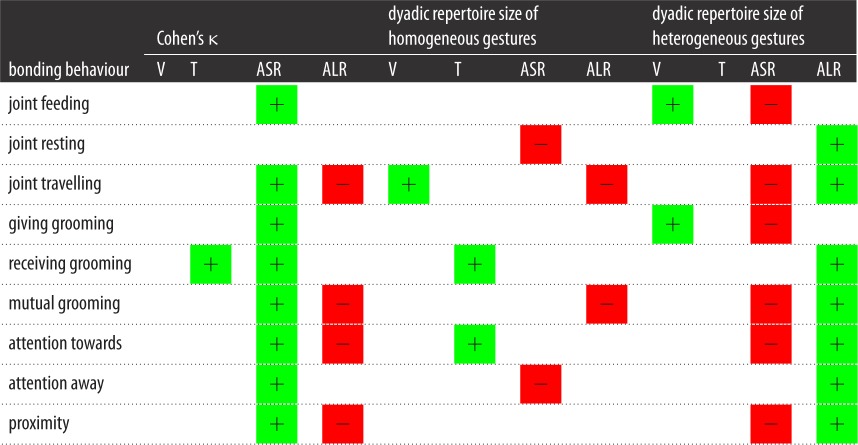

### 3.4. Dyadic repertoire size of homogeneous and heterogeneous gestures and social bonding

The next set of analysis used MRQAP to examine the associations between the homogeneous dyadic repertoire size (the number of gestures each dyad shares) and the bonding behaviours. The information pertaining to the mean value of ties of dyadic repertoire size of the homogeneous gesture network is given in table 5. This dyadic repertoire size was based on the total number of gesture types that each focal individual had in common with the dyad partner, rather than the number of homogeneous gesture types shared between the signaller and the recipient in a sequence of gestures used in the GLMMs. When the four modalities were considered in one model, for visual gestures, the dyadic repertoire size was positively associated with the duration of joint travel (*r*^2^ = 0.154, *β* = 0.417, *p* = 0.044). The dyadic repertoire of tactile gestures was positively associated with the duration of grooming received (*r*^2^ = 0.142, *β* = 0.367, *p* = 0.011) and attention towards (*r*^2^ = 0.203 *β* = 0.398, *p* = 0.040). By contrast, for auditory short-range gestures, the dyadic repertoire size was negatively associated with the duration of attention away (*r*^2^ = 0.118, *β* = −0.273, *p* = 0.026) and joint resting (*r*^2^ = 0.103, *β* = −0.273, *p* = 0.012). Finally, the dyadic repertoire size of auditory long-range gestures was negatively related to the duration of mutual grooming (*r*^2^ = −0.136, *β* = −0.414, *p* = 0.024 and joint travel (*r*^2^ = 0.154, *β* = −0.366, *p* = 0.033). Table 6 presents a summary of these results.

Table 5 gives the mean value of ties of dyadic repertoire size of the heterogeneous gesture networks. In this section, we used MRQAP to examine the associations between the heterogeneous dyadic repertoire size (the number of gestures each dyad did not share) and the bonding behaviours. This repertoire size counted the total number of gesture types that the focal subject had but the non-focal subject did not have in their repertoire and this was added to the total number of gesture types that the non-focal subject had but the focal subject did not have in their repertoire of gestures. For visual gestures, there was a positive association between repertoire size and duration of grooming given (*r*^2^ = 0.285, *β* = 0.220, *p* = 0.044) and joint feeding (*r*^2^ = 0.285, *β* = −0.184, *p* = 0.049). For auditory long-range gestures, there was a positive association between the repertoire size and duration of proximity (*r*^2^ = 0.215, *β* = −0.391, *p* = 0.002), rest (*r*^2^ = 0.087, *β* = −0.213, *p* = 0.049), travel (*r*^2^ = 0.160, *β* = 0.285 *p* = 0.031), grooming mutual (*r*^2^ = 0.166, *β* = 0.399, *p* = 0.009), grooming received (*r*^2^ = 0.122, *β* = 0.358, *p* = 0.009), attention away (*r*^2^ = 0.120, *β* = −0.266, *p* = 0.030) and attention towards (*r*^2^ = 0.258, *β* = 0.380, *p* = 0.006). By contrast, for auditory short-range gestures, the dyadic repertoire size of heterogeneous gestures was negatively associated with the duration of proximity (*r*^2^ = 0.215, *β* = −0.259, *p* = 0.013), the duration of travel (*r*^2^ = 0.160, *β* = −0.198, *p* = 0.038), grooming mutual (*r*^2^ = 0.166, *β* = −0.273 *p* = 0.011), grooming given (*r*^2^ = 0.174, *β* = −0.193, *p* = 0.037), attention towards (*r*^2^ = 0.285, *β* = −0.329, *p* = 0.006) and joint feeding (*r*^2^ = 0.186, *β* = −0.231, *p* = 0.005). Table 6 presents a summary of these results.

Using gesture present or absent as a dependent variable, we further examined how homogeneous gesture presence or absence was predicted by demography and social bonding variables for visual and auditory long-range gestures using GLMM. Visual homogeneous gestures were associated with a longer duration of time spent resting (*β* = 0.847, s.e. = 0.270, *p* = 0.002) and receiving grooming (*β* = 0.355, s.e. = 0.128, *p* = 0.006) but a shorter duration of time visually attending (*β* = −0.731, s.e. = 0.201, *p* < 0.001) and non-attending (*β* = −0.989, s.e. = 0.367, *p* = 0.007) to a dyad partner who was more probably maternal kin (*β* = −4.935, s.e. = 1.087, *p* = 0.001) of the signaller. Homogeneous auditory long-range gestures were associated with a longer duration of time spent in joint feeding (*β* = 0.598, s.e. = 0.277, *p* = 0.032) and travel (*β* = 1.326, s.e. = 0.233, *p* < 0.001) with individuals who were unrelated through maternal kinship (*β* = 7.696, s.e. = 1.552, *p* < 0.001). However, auditory long-range homogeneous gestures were associated with shorter duration of time receiving grooming (*β* = −0.237, s.e. = 0.111, *p* = 0.033) and time spent in close proximity (*β* = −0.459, s.e. = 0.183, *p* = 0.012).

### 3.5. Network size

The next set of analyses used node-level regression to examine the relationship between bonding network centrality (variance in the extent to which individual chimpanzees received bonding behaviours from conspecifics) and homogeneity centrality (variance in the extent to which the gestural repertoires of individual chimpanzees overlapped with the gestural repertoire of conspecifics). The values of network centrality for the variables significantly associated with each other can be found in table 7. In these models, the bonding behaviours were the dependent variable (e.g. proximity indegree) and the predictor variables were the homogeneity centrality (based on Cohen's κ). In all models, the control variables were included relating to kinship, age, sex and duration of time spent within 10 m of an oestrus female.

**Table 7.** The repertoire homogeneity *n* degree (Cohen's κ coefficient) and social behaviour centrality (only variables showing significant associations with homogeneity of repertoire are given) across *N* = 10 chimpanzees. Cohen's κ coefficient ranges from −1 (completely different repertoire) to +1 (exactly the same repertoire). The highest values of centrality are indicated in italics. Only gestures produced by the focal adult chimpanzee to another adult are included.

	the repertoire homogeneity *n* degree (Cohen's κ coefficient)^a^				social behaviour indegree and outdegree^b^			
focal subject ID	auditory long-range	auditory short-range	tactile	visual	attention away indegree	groom received indegree	joint rest outdegree	joint feed indegree
BB	0.239	0.446	0.209	0.329	2.813	0.75	*1.25*	1.208
HW	0.324	0.24	0.137	0.337	2.735	*1.349*	0.949	0.494
KT	0.251	0.308	0.145	0.323	0.534	0	0.38	0.335
KW	0.157	0.339	0.197	0.234	1.054	0	0	0.303
ML	0.01	−0.087	0.047	0.294	1.93	0.235	1.086	1.365
MS	*0*.*356*	*0*.*446*	0.172	0.317	*3.879*	0.899	0.367	*2.365*
NB	−0.048	−0.124	0.109	0.145	0.219	0.342	0.464	0.207
NK	0.265	0.446	0.21	0.295	3.263	0.191	0.807	0.853
RH	0.157	0.303	0.142	0.223	0	0	0.777	0
SQ	0.303	0.271	*0.305*	*0.348*	1.254	0.805	0.61	0.259

^a^The gesture network is undirected therefore normalized degree centrality (*n* degree) is calculated. Normalized degree centrality is the average value of each row or column of the network matrix i.e. the average value of that behaviour for each focal chimpanzee.

^b^The social bonding network is directed, therefore indegree and outdegree are calculated separately. Outdegree refers to behaviours directed by the focal chimpanzee to conspecifics, whilst indegree refers to behaviours directed by conspecifics towards the focal chimpanzee.

In models containing all four modalities, for auditory short-range and tactile gestures, there was no significant relationship between homogeneity centrality and bonding behaviours. For visual gestures, homogeneity centrality was only related to outdegree for joint resting (*r*^2^ = 0.721, *β* = 2.168, *p* = 0.049). By contrast, homogeneity centrality for auditory long-range gestures was related to indegree for joint feeding (*r*^2^ = 0.789, *β* = 2.801, *p* = 0.046), indegree for receiving grooming (*r*^2^ = 0.914, *β* = 3.445, *p* = 0.019) and indegree for attention away (*r*^2^ = 0.714, *β* = 2.581, *p* = 0.049). Table 8 presents a summary of these findings.

**Table 8.** Summary of results of node-level regressions predicting indegree (IN) and outdegree (OUT) from homogeneity centrality for the four modalities included in the same model: visual (V), tactile (T), auditory short-range (ASR) and auditory long-range (ALR). Green squares indicate a positively significant relationship between the two variables. Blank squares indicate a relationship that was not statistically significant.

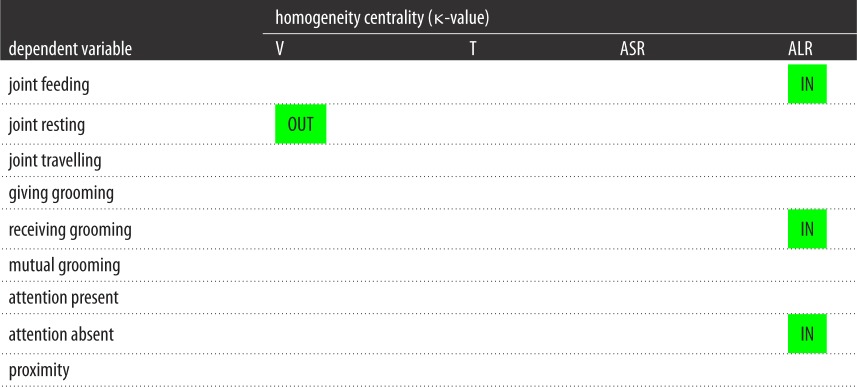

Furthermore, we used node-level regression to examine the relationship between bonding network centrality (variance in the extent to which individual chimpanzees received bonding behaviours from conspecifics) and centrality in the repertoire size of homogeneous and heterogeneous gestures. In these models, the repertoire size of homogeneous gestures and the repertoire size of heterogeneous gestures by modality were included as predictor variables in all models. We included both homogeneous and heterogeneous gestures in one model, including all four modalities of gestures. Chimpanzees that had a larger repertoire size of homogeneous visual gestures had a higher outdegree of proximity (*r*^2^ = 1, *β* = 4.795, *p* = 0.015), resting (*r*^2^ = 1, *β* = 5.612, *p* = 0.002), travel (*r*^2^ = 1, *β* = 4.213, *p* = 0.030), grooming received (*r*^2^ = 1, *β* = 4.614, *p* = 0.024), grooming mutual (*r*^2^ = 1, *β* = 5.330, *p* = 0.009), attention present (*r*^2^ = 1, *β* = 4.680, *p* = 0.019) and attention absent (*r*^2^ = 1, *β* = 4.779, *p* = 0.019). Chimpanzees that had a smaller repertoire size of homogeneous tactile gestures had a higher outdegree of proximity (*r*^2^ = 1, *β* = −3.032, *p* = 0.042), resting (*r*^2^ = 1, *β* = −3.726, *p* = 0.012), travel (*r*^2^ = 1, *β* = −3.013, *p* = 0.040) and attention absent (*r*^2^ = 1, *β* = −3.305, *p* = 0.036). Moreover, the larger repertoire size of homogeneous auditory long-range gestures was associated with the higher indegree of joint feeding (*r*^2^ = 1, *β* = 4.499, *p* = 0.032). By contrast, the smaller repertoire size of homogeneous auditory long-range gestures was associated with a higher outdegree of resting (*r*^2^ = 1, *β* = −3.331, *p* = 0.030), grooming received (*r*^2^ = 1, *β* = −3.196, *p* = 0.049) and grooming mutual (*r*^2^ = 1, *β* = −3.742, *p* = 0.027). Finally, focal chimpanzees that had a smaller repertoire size of heterogeneous auditory short-range gestures had a higher indegree of feeding (*r*^2^ = 1, *β* = −1.031, *p* = 0.014), grooming given (*r*^2^ = 1, *β* = −1.045, *p* = 0.010) and grooming mutual (*r*^2^ = 1, *β* = −0.793, *p* = 0.047). These results are summarized in table 9.

**Table 9.** Summary of results of node-level regressions predicting indegree (IN) and outdegree (OUT) from the repertoire size of homogeneous and heterogeneous gestures centrality for the four modalities combined in one model: visual (V), tactile (T), auditory short-range (ASR) and auditory long-range (ALR). Green squares indicate a positively significant relationship between the two variables; red squares indicate a negatively significant relationship. Blank squares indicate a relationship that was not statistically significant.

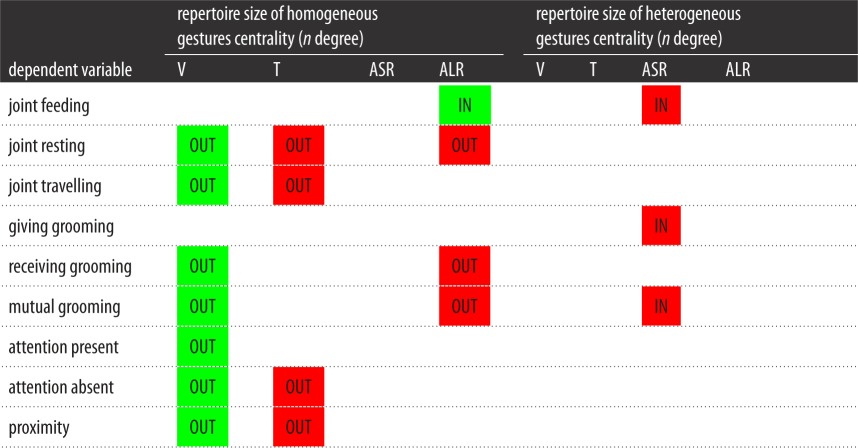

### 3.6. Rate of gesturing

#### 3.6.1. Homogeneity network and rate of gesturing network

For visual gestures, the rate of gesturing was positively related to dyadic homogeneity, as measured by Cohen's κ (*r*^2^ = 0.079, *β* = 0.301, *p* = 0.023). There was no significant relationship between the rate of gesturing and dyadic homogeneity for tactile, auditory short-range or auditory long-range gestures.

#### 3.6.2. Dyadic repertoire size of homogeneous network and rate of gesturing

The dyadic repertoire size of homogeneous gestures was positively related to the rate of gesturing for visual gestures (*r*^2^ = 0.122, *β* = 0.351, *p* = 0.002) and tactile gestures (*r*^2^ = 0.133, *β* = 0.352, *p* = 0.003). There were no significant relationships between the dyadic repertoire size of heterogeneous gestures and the rate of gesturing. These results are summarized in table 10.

**Table 10.** Summary of results MRQAP regression models predicting Cohen's κ, dyadic repertoire size of homogeneous gestures and dyadic repertoire size of heterogeneous gestures from the rate of gesturing between pairs of chimpanzees. Green squares indicate a positively significant relationship between the two variables. Blank squares indicate a relationship that was not statistically significant.

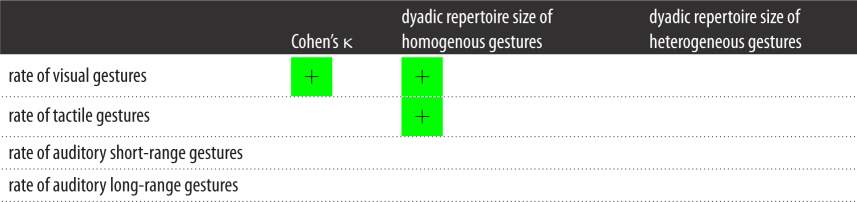

### 3.7. Audience size

In the next set of analyses, we used presence of homogeneous or heterogeneous gestures as a dependent variable and examined the influence of audience size on the likelihood of these gestures being present. The presence of a heterogeneous gesture was significantly more likely than the presence of a homogeneous gesture when the signaller gestured to a reproductively active partner (*β* = 2.613, s.e. = 0.916, *p* = 0.005), the audience size of same-age partners as the signaller was larger (*β* = −1.855, s.e. = 0.754, *p* = 0.015) and the audience size of different-age partners as the signaller was smaller (*β* = 0.447, s.e. = 0.137, *p* = 0.001). There was a higher likelihood of homogeneous gesture being used, when compared with a heterogeneous gesture, when the partner was reproductively inactive (*β* = 2.676, s.e. = 0.842, *p* = 0.002) and the audience size of opposite-sex partners relative to the signaller was larger (*β* = 1.106, s.e. = 0.393, *p* = 0.005). The audience size of same-sex partners did not influence the likelihood of a homogeneous or heterogeneous gesture being present (*β* = −0.443, s.e. = 0.378, *p* = 0.243).

### 3.8. Response to gestures

We examined the relationship between homogeneous and heterogeneous gestures and the responses by the recipient. Overall, gesture sequences in which homogeneity was present were more likely to elicit a response, when compared with sequences in which heterogeneous gestures were present (*β* = −0.823, s.e. = 0.332, *p* = 0.014). Further, this response to homogeneous gestures was more likely to be by means of an emotional display. By contrast, heterogeneous gestures were more likely to evoke a response by means of goal-directed action towards the recipient (*β* = −1.326, s.e. = 0.603, *p* = 0.029).

We then examined the relationship between repertoire size of homogeneous gestures by modality and the response by the recipient within sequences. The homogeneous repertoire size of visual gestures was positively related to a presence of response (*β* = 0.016, s.e. = 0.242, *p* = 0.012). However, the repertoire size of homogeneous auditory short-range gestures was negatively related to a presence of response (*β* = −1.816, s.e. = 0.823, *p* = 0.028). When comparing goal-directed and emotional response in relation to the repertoire size of homogeneous gestures within sequences, we found that the larger repertoire size of both homogeneous auditory short-range gestures (*β* = 1.053, s.e. = 0.409, *p* = 0.011) and homogeneous auditory long-range gestures (*β* = 2.923, s.e. = 0.537, *p* < 0.001) was positively associated with emotional responses to the gestures. Finally, we examined whether the emotional response to the gesture was by vocal response (presence or absence). We found that the larger repertoire size of homogeneous auditory longer-range gestures was associated with vocal response (*β* = 2.766, s.e. = 0.618, *p* < 0.001).

Finally, we analysed how the repertoire size of heterogeneous gestures by modality was associated with the response (presence or absence and type) by the recipient within sequences. Overall, a larger repertoire size of heterogeneous gestures by modality was not associated with presence or absence of a response. When examining by the response type (goal-directed or emotional display), we found that a larger repertoire size of auditory short-range heterogeneous gestures was positively associated with goal-directed response (*β* = −14.435, s.e. = 0.297, *p* < 0.001). The response present in the form of vocal display was more likely in relation to a larger repertoire size of heterogeneous auditory long-range gestures (*β* = 0.423, s.e. = 0.172, *p* = 0.014) and less likely in relation to a larger repertoire size of heterogeneous auditory short-range gestures (*β* = −12.486, s.e. = 0.308, *p* < 0.001).

## 4. Discussion

One key question in evolutionary anthropology is how, with increasing group size through hominin evolution, large groups of individuals cooperated with each other to coordinate their behaviour and maintain group cohesion [100]. Grooming fosters trust and cooperation between regular interaction partners, but time and cognitive demands on grooming limit the number of social relationships that can be maintained using grooming alone [101]. Language is an open-ended system of conventional signals that facilitates social interactions between unrelated individuals [102]. It has been suggested that the evolution of language enabled the growth of complex social systems during human evolution [8]. Gestural communication is frequently thought of being an important predecessor to language, but its role in managing social relationships has not been explored [19]. In particular, it is unclear whether language evolution involved intentional (goal-directed) or emotional gestural expressions. We used a dataset of gestures produced by wild adult chimpanzees towards other adult conspecifics to examine how the use of homogeneous (overlapping) and heterogeneous (not overlapping) gestural repertoires relates to sociality.

First, we examined the extent to which chimpanzees used heterogeneous gestures to manage social relationships with conspecifics. A larger repertoire of heterogeneous gestures (visual, auditory long range) was associated with a longer duration of time spent in social bonding behaviour with group members representing different-age, opposite sex classes to the signaller and similar reproductive status (male-oestrous female) but not with a larger number of these individuals (larger size of social networks). By contrast, a larger repertoire size of heterogeneous auditory short-range gestures was associated with a shorter duration of time spent in bonding behaviours and also a smaller social network. Thus, chimpanzees that used a larger repertoire of heterogeneous auditory short-range gestures had less established social networks with conspecifics from different age and opposite sex classes or of similar reproductive status. The advantage of heterogeneous gestures is that they increase the salience of the signaller to the recipient, making the interaction more effective in the face of competition with conspecifics. Chimpanzees used heterogeneous gestures more often with reproductive partners when there was a smaller audience of opposite-sex and different-age partners, but a larger audience of same-sex and same-age partners as the signaller in close proximity, suggesting that chimpanzees used goal-directed gestures to compete with similar others for the attention of the recipient. However, as heterogeneous gestures are not specifically directed at the recipient and have contextually inferred meaning, the perception of heterogeneous gestures may be cognitively demanding and involve areas of the brain typically associated with complex cognition [103]. Especially, lower intensity visual gestures are associated with lower specificity and directedness when compared with more intense tactile and auditory gestures [64] and therefore the constraints on perception of heterogeneous gestures may be exacerbated by the visual modality of gesturing [95]. Single, heterogeneous gestures are an optimal mode of communication between kin, individuals of same age and sex class, who spend long time periods engaged in social behaviours. In these circumstances, heterogeneous gestures are efficient as no directedness or specificity is employed during communication to coordinate behaviour such as foraging in close proximity. Moreover, these gestures are effective as familiarity enables recipient to infer that they are target of communication and mutual understanding of context enables recipient to infer meaning of the gesture [95]. However, when employed in interactions with dyad partners who are unrelated or represent different-age or opposite sex classes these gestures may be ineffective because the time spent in social behaviour with these individuals is shorter [95] and thus ability of the recipient to contextually interpret the heterogeneous gesture from the history of past interactions is hampered [64]. Homogeneity (overlap) in gestural repertoire can reduce these constraints on social relationships imposed by perception of heterogeneous gestures by increasing heterogeneous gestures' specificity and directedness. Heterogeneous gestures frequently co-occur with homogenous gestures within sequences when dyad partners are unrelated or represent different-age or opposite sex classes [95]. In contrast with heterogeneous gestures present within sequences, homogeneous gestures were associated with longer duration of time spent in social bonding behaviour. Moreover homogeneity of gestures was associated with larger social networks than heterogeneity of gestures. These results suggest that the inclusion of homogenous gestures within sequences of heterogeneous gestures plays a role of reducing constraints on contextual perception of heterogeneous communication with unrelated dyad partners or partners from different-age or opposite sex classes to enable more complex social relationships [64,95].

Second, we examined whether use of homogeneous gestures is associated with social bonding in chimpanzees. Chimpanzee dyads that were related through maternal kinship or were similar in age and sex characteristics spent a longer duration of time engaged in bonding behaviours such as grooming. However, when homogeneity in gestures was included in the model, the results demonstrated that the duration of time spent in bonding behaviours was also related to overlap in the repertoire of gestures between dyads irrespective of kinship and familiarity. Homogeneous gestures tended to elicit an emotional display in the recipient, which indicates that communicative similarity plays an important role in shaping social relationships, possibly through the automatic action of social neurohormones [104–106].

In particular, a large repertoire of homogeneous visual and tactile gestures was associated with a longer duration of time spent in social bonding behaviour with maternal kin and individuals of the same sex and age class. This suggests that homogeneity is driven through repeated instances of gestural interaction with regular social partners, rather than more passive acquisition based on the presence of a social relationship. These gestures may be better suited to maintaining social relationships over longer periods and this gives chimpanzees more opportunity for convergence in the repertoires of these gestures. Thus, pairs of chimpanzees preferentially affiliate with each other and the higher the rate of gesturing, the higher the degree of homogeneity and the larger the repertoire size of homogeneous visual and tactile gestures that a dyad can use in their interactions with each other.

It could be argued that dyads who were close in age displayed a higher degree of homogeneity in visual and tactile gestures because the size of the gestural repertoire is negatively related to age in great apes and this decline contributes to homogeneity in repertoire [51,52,107]. However, when controlling for the demographic differences within the dyads, there were still significant associations between repertoire homogeneity and bonding behaviours. The fact that chimpanzees direct homogeneous visual and tactile gestures at same-age and same-sex partners suggests that the patterns of homogeneity in these gestures are shaped by patterns of social interaction rather than by shaping of the repertoire in ontogeny. Further, the demographic characteristics of the individuals who are present in close proximity during interactions influence the patterns of homogeneity. For instance, when the number of same-age partners in the immediate audience is smaller but the number of different-age partners is larger, chimpanzees use homogeneous gestures. Detailed longitudinal data on gestural development should address the relative roles of ontogenetic ritualization [30,35], different types of social learning [108] and biological factors (e.g. age, sex) in repertoire homogeneity [51,109].

While grooming is a key behaviour primates use to maintain relationships with a small set of key social partners [43], homogeneity in visual gestural communication may be an alternative mechanism used to meet one of the key challenges of social living—closely coordinating behaviour with others in proximity [110]. Chimpanzees who shared a larger repertoire of homogeneous visual gestures had more complex social networks with conspecifics (i.e. they interacted with a larger number of chimpanzees for longer). By contrast, social networks were smaller when chimpanzees shared a larger repertoire of tactile gestures, suggesting these gestures were unsuitable for bonding with numerous individuals [45,64]. This may be particularly true in a fission–fusion species, where failure to coordinate behaviour can lead to the spatial separation of individuals [110]. The perception of homogeneous visual gestures could be automatic and involve the action of mirror neurons, therefore reducing the constraints on managing social relationships [103,111,112].

Moreover, homogeneity in auditory long-range gestures showed a different association with the duration of time spent in bonding behaviours. The duration of bonding behaviours was negatively associated with overlap in the repertoire of auditory long-range gestures, so pairs of chimpanzees that spent less time in these bonding behaviours had a greater overlap in auditory long-range gestural communication. Further, at the level of the sequence, chimpanzees were more likely to spend a longer duration of time with unrelated individuals in social bonding behaviours (joint feeding, travelling) when using homogeneous auditory long-range gestures. These findings suggest that homogeneity in auditory long-range gestures functions to prolong the duration of time spent in social bonding behaviour with unrelated individuals, with whom relationships are weaker. This interpretation is consistent with the suggestion that homogeneity in auditory long-range gestures can increase the similarity between unrelated individuals, facilitating social relationships.

Further, an association between centrality for social bonding behaviour and centrality for homogeneous auditory long-range gestures indicates the importance of these gestures in maintaining complex social networks. Chimpanzees who shared a large repertoire of homogeneous auditory long-range gestures displayed less complex grooming networks and more complex joint feeding networks, suggesting that these chimpanzees were able to feed in close proximity to a larger number of conspecifics over longer periods. Thus, in the absence of well-developed grooming networks, these gestures may function as a means to establish feeding tolerance with a larger number of unrelated individuals. In contrast, chimpanzees who shared a small repertoire of homogenous auditory long-range gestures had large grooming networks, suggesting these signals may act as a badge of status. These data clearly show that in addition to maintaining a small set of strong social relationships, chimpanzees living in larger social groups maintain a larger set of weaker social relationships, to enable the group to function as a cohesive whole [113,114]. This type of bonding seems to be important in species maintaining large social groups, as auditory gestures have not been observed in apes maintaining smaller groups such as orangutans [89,115].

In fission–fusion societies where sufficient encounters cannot occur naturally between geographically dispersed individuals [116], behavioural strategies to create social bonds operate at a distance [67,117,118]. Here, we provide evidence that it is specifically homogeneous auditory long-range gestures that could function at a distance to establish social bonds with weakly bonded dyad partners [45,64,67,68]. By definition auditory long-range gestures such as drumming have a high amplitude and can affect the receivers without direct visual contact. In this study, all instances of auditory long-range gestures involved the use of objects from the external environment, such as a branch of a tree. Object use in auditory long-range gestures can function as an external cue that approximates phenotypic similarity and promotes tolerance. Thus, by incorporating objects in the display, overlap in the repertoire of auditory long-range gestures is one mechanism that can bond individuals on a larger scale, without the need for frequent, direct one-to-one interaction. Given the high costs of familiarity and individual recognition, overlap in object use to make homogeneous loud auditory gestures can identify unrelated chimpanzees as members of the same group, enabling relationships of trust to develop between these individuals and disambiguate them from members of other groups (‘symbolic marking’) [9,119]. Chimpanzees can match the use of the object directly during joint display, or can adopt the use of the object on a larger scale without the need of being directly involved in social interaction with the signaller. Object use frequently involves use of rhythmic repetition in the context of high-intensity interaction, which could facilitate homogenization of these cues by the action of social neurohormones [104–106].

By contrast, for auditory short-range gestures, overlap in repertoire appears to be affected by social factors in a way predicted by the iterated learning experiments with humans [55]. There was a positive relationship between duration of time spent in social bonding behaviour and homogeneity in auditory short-range gestures. However, the repertoire size of homogeneous auditory short-range gestures was negatively associated with the duration of time spent in social bonding behaviour, suggesting that the chimpanzee dyads converged by reducing the repertoire of homogeneous gestures over time, to enable more efficient communication with dyad partners. Because a smaller repertoire of homogeneous auditory short-range gestures appears to be more efficient in eliciting a response from the recipient and also a smaller repertoire of these gestures is more likely to elicit a goal-directed response, these gestures may act as an efficient way to communicate with the regular interaction partners, with whom the focal chimpanzee coordinates behaviour in a goal-directed manner.

Managing a complex set of social relationships is a central challenge for all animals which live in large and stable groups [120]. Goal-directed (heterogeneous) and emotional (homogeneous) gestures can facilitate relationships with regular interaction partners. On the other hand, weak social relationships are cognitively complex and time-intensive to manage and this is particularly the case in fission–fusion chimpanzee groups, where individuals are often temporarily and spatially separated, but have to remember the identities and past histories with all members of the group [116]. This study provides the first evidence that homogeneous auditory long-range gestures are related to patterns of social behaviour in primates. Thus, increasing similarity through the use of external cues such as objects in the natural environment may help reduce the time and cognitive constraints associated with managing weak social relationships. In particular, homogeneity in a large repertoire of long-range auditory gestures may facilitate social interactions after long periods of absence. By experiencing high-intensity emotions simultaneously with other group members through a large repertoire of shared auditory long-range gestures, individuals can re-establish social bonds and this appears to play a key role in enabling the growth of a larger social system, by acting as a replacement for grooming [45]. Future studies will shed light on the important role repertoire homogeneity and heterogeneity plays both in shaping patterns of affiliation and identity, and in facilitating social bonding and cooperation, across large and dispersed social groups in non-human primates and hominins [9–15,105,108,112,121–123].

## Supplementary Material

Supplementary Information 1

## Supplementary Material

Supplementary Information 2

## Supplementary Material

Supplementary Information 3

## Supplementary Material

Supplementary Information 4

## Supplementary Material

Supplementary Information 5

## Supplementary Material

Supplementary Information 6

## Supplementary Material

Supplementary Information 7

## Supplementary Material

Supplementary Information 8

## Data accessibility

The data associated with this research are available in the electronic supplementary material, S2–S8.
