# Supplementary material for: Convergence and divergence in gesture repertoires as an adaptive mechanism for social bonding in primates
Source: R Soc Open Sci. 2017 Nov 29;4(11):170181. doi: 10.1098/rsos.170181 (PMC5717623; doi:10.1098/rsos.170181)
Supplement: Supplementary Information 1 [file rsos170181supp1.docx]

**Convergence and divergence in gesture repertoires as an adaptive mechanism for social bonding in primates**

**Royal Society Open Science**

Anna Ilona Roberts and Sam George Bradley Roberts

**Supplementary Information 1**

**Example of how behavioural measures for networks were calculated (see[1, 2] for full details)**

The dyadic association measure to 2 meters

The dyadic association measure (DA2) is the duration of time focal subject A spent in close proximity (within 2 meters) to non-focal subject B, when being its nearest neighbour per hour spent in the same party, or:

DA2_AB_ = {(P2_AB_*2)* 60)} /PSP_AB_*2

where P2_AB_ = the number of times A was in close proximity (within 2 m) to B when its nearest neighbour

PSP_AB_ = the number of times A was in the same party as B

2 = duration of instantaneous subsample interval in minutes

60 = the number of minutes in an hour

**Supplementary Table 1 Categorisation of attributes**

| Category of attribute | Subcategory of attribute | Description of subcategory |
| --- | --- | --- |
| Kinship similarity | Kin | mother-son  son-mother |
|  | Non-kin | unrelated dyad |
| Sex similarity | Same sex | male-male  female-female |
|  | Different sex | male-female  female-male |
| Oestrous similarity | Reproductively active | male-oestrous female |
|  | Reproductively incactive | unoestrous female- unoestrous female  unoestrous female-oestrous female  oestrous female-oestrus female  unoestrous female-male  male-male |
| Age proximity | Same age category | no more than 5 years age difference between individuals in the dyad |
|  | Different age category | more than 5 years age difference between individuals in the dyad |
| Sex | Male  Female |  |
| Age | Young adult | 16 years old or younger |
|  | Mature adult | More than 16 years of age |

**Supplementary Table 2. Classification of gesture types according to modality**

| Gesture modality | Gesture types* |
| --- | --- |
| Visual gesture | Arm beckon, Arm drop, Arm flap, Arm raise, Backward sweep, Bend shrub, Bob, Bow, Crouch, Crouch run, Crouch walk, Cupped extend, Dangle, Drag self, Drop by hand, Drop by mouth, Elbow raise, Fist flail, Forceful extend, Hand bend, Hand reach, Hand swing, Hang, Hold object, Jump, Limp extend, Linear sweep, Lower back, Lower head, Lunge, Nod, Offer by hand, Present arm, Present genitals, Present leg, Present mount, Present rump, Present torso, Retrieve, Rock, Roll object, Roll over, Rounded sweep, Run stiff, Sharp reach, Slap self, Sniff, Somersault, Stationary stiff, Stiff extend, Stretched extend, Stretched reach, Strip leaf, Swagger bipedal, Swagger quadrupedal, Swagger stationary, Thrust vacuum, Tip head, Touch self, Turn back, Turn head, Unilateral swing, Vertical extend, Walk stiff, Wipe, |
| Tactile gesture | Bite, Embrace, Grab, Hold hands, Kiss, Locomote tandem, Offer hand, Poke, Pull another, Push by hand, Push by rump, Rub, Scoop, Shake limb, Shove, Slap another, Slide, Squeeze, Stand tandem, Stroke by mouth, Stroke long, Stroke short, Tap another, Thrust genitals, Tickle, Touch backhand, Touch innerhand, Touch long, Touch with leg |
| Auditory short range gesture | Clip by hand, Clip by mouth, Inspect, Pull object, Smack lip, Tap foot, Tap object |
| Auditory long range gesture | Beat, Bounce, Break, Drag object, Drum, Hand clap, Hit object, Hit object to object, Kick, Knock, Pound, Shake mobile, Shake stationary, Shuffle, Slap object, Stamp quadrupedal, Stamp sitting, Sway, Swing, |

^*^Description and video footage of gesture types can be found in Roberts A.I., Roberts S.G.B., Vick S.-J. 2014 The repertoire and intentionality of gestural communication in wild chimpanzees. *Animal Cognition* **17**, 317 – 336 and Roberts A.I., Vick S.-J., Roberts S.G.B., Buchanan-Smith H.M., Zuberbühler K. 2012 A structure-based repertoire of manual gestures in wild chimpanzees: statistical analyses of a graded communication system. *Evolution and Human Behavior* **33**(5), 578-589.

**Repertoire size of homogeneous and heterogeneous gestures and social bonding at the level of the sequence type**

We first used GLMMs to examine how the repertoire size of homogenous and heterogeneous gestures within gesture sequences was related to bonding behaviours. For the overall repertoire size (including all modalities), the dependent variables was the repertoire size and the predictor variables were the bonding behaviour (e.g. duration of time pair of chimpanzees spent engaged in joint feeding, per hour spent in the same party) along with the control variables (sex difference, age difference, oestrous difference, maternal kinship, response present or absent). For the analysis by modality, because we considered all modalities in one model, the dependent variables were the bonding behaviour (e.g. joint feeding) and the predictor variables were the repertoire size for the different modalities (visual, tactile, auditory short-range, auditory long-range) and the control variables (sex difference, age difference, oestrous difference, maternal kinship, response present or absent).

The overall homogenous repertoire size, which includes all modalities together, was positively related to the oestrous difference (β = 0.563, SE = 0.125, *p* < 0.001) and the duration of a large number of bonding behaviours: joint feeding (β = 0.087, SE = 0.041, *p* = 0.036), joint resting (β = 0.235, SE = 0.062, *p* < 0.001), joint travel (β = 0.226, SE = 0.048, *p* < 0.001), give groom (β = 0.150, SE = 0.062, *p* = 0.016) and mutual groom (β = 0.113, SE = 0.043, *p* = 0.009). However, the overall homogenous repertoire size was negatively associated with attention present (β = -0.100, SE = 0.036, *p* = 0.005), attention absent (β = -0.205, SE = 0.063, *p* = 0.001) and maternal kinship (β = -0.742, SE = 0.233, *p* = 0.002).

The overall heterogeneous repertoire size was negatively related to joint feeding (β = -0.134, SE = 0.064, *p* = 0.037), joint resting (β = -0.135, SE = 0.063, *p* = 0.031), joint travel (β = -0.232, SE = 0.097, *p* = 0.018), give groom (β = -0.213, SE = 0.059, *p* < 0.001) and receive groom (β = -0.144, SE = 0.037, *p* < 0.001). However, for attention present (β = 0.076, SE = 0.027, *p* = 0.003) and attention absent (β = 0.173, SE = 0.084, *p* = 0.044), there was a positive association between the heterogeneous gesture repertoire size and duration of time spent in the activity.

We then considered the homogenous repertoire size for each modality separately. In terms of influence of demographic relationship between signaller and the recipient, there was a negative association between sex difference and the duration of time spent mutually grooming (β = - 1.555, SE = 0.639, *p* = 0.016), mutually visually attending (β = - 3.259, SE = 1.173, *p* = 0.006), visually non attending (β = - 1.639, SE = 0.329, *p* < 0.001) and proximity (β = - 4.966, SE = 1.273, *p* < 0.001). Moreover, there was a negative association between age difference and the duration of time spent in following behaviour: jointly resting (β = - 1.866, SE = 0.918, *p* = 0.043), giving grooming (β = - 2.490, SE = 0.799, *p* = 0.002), visually non attending (β = - 2.622, SE = 1.324, *p* = 0.049), proximity (β = - 10.581, SE = 4.901, *p* = 0.032). Further, oestrous difference showed a pattern of significant negative association with mutual grooming (β = - 1.699, SE = 0.626, *p* = 0.007), mutually visually attending (β = - 3.103, SE = 1.068, *p* = 0.004), proximity (β = - 2.981, SE = 1.425, *p* = 0.037). Finally, maternal kinship was significantly negatively associated with the duration of time spent in the following social bonding behaviours: joint feeding (β = - 1.856, SE = 0.244, *p* = 0.001), jointly resting (β = - 2.006, SE = 0.389, *p* = 0.001), giving grooming (β = - 3.653, SE = 0.494, *p* < 0.001), receiving grooming (β = - 1.686, SE = 0.553, *p* = 0.003), mutually grooming (β = - 3.761, SE = 0.880, *p* < 0.001), mutually visually attending (β = - 10.123, SE = 1.677, *p* < 0.001), mutually visually non attending (β = - 8.756, SE = 0.266, *p* < 0.001) and close proximity (β = - 17.806, SE = 1.511, *p* < 0.001).

For visual gestures, the homogenous repertoire size was positively associated with the duration of mutual grooming (β = 0.755, SE = 0.244, *p* = 0.002) and attention present (β = 1.136, SE = 0.370, *p* = 0.002) between the signaler and receiver. However, the homogenous repertoire size for visual gestures was negatively associated with the duration of receiving grooming (β = -0.327, SE = 0.118, *p* = 0.005) and attention absent (β = -0.563, SE = 0.270, *p* = 0.038).

For tactile gestures, homogenous repertoire size was positively associated with the duration of a large number of bonding behaviours: joint resting (β = 0.422, SE = 0.128, *p* = 0.001), joint travelling (β = 0.499, SE = 0.230, *p* = 0.031), giving grooming (β = 0.360, SE = 0.110, *p* = 0.001), mutual grooming (β = 1.490, SE = 0.596, *p* = 0.013), attention present (β = 2.248, SE = 0.943, *p* = 0.018), and proximity (β = 2.148, SE = 0.830, *p* = 0.010). The homogenous repertoire size for tactile gestures was negatively related to joint feeding (β = -0.597, SE = 0.249, *p* = 0.017) and receiving grooming (β = -0.339, SE = 0.101, *p* = 0.001).

Auditory short-range gestures were also positively associated with duration for a number of bonding behaviours: joint feeding (β = 0.855, SE = 0.386, *p* = 0.028), joint resting (β = 0.698, SE = 0.306, *p* = 0.023), giving grooming (β = 1.166, SE = 0.281, *p* < 0.001) and attention absent (β = 1.141, SE = 0.484, *p* = 0.019). In contrast, the homogenous repertoire size for auditory long-range gestures was negatively associated duration for four bonding behaviours: joint feeding (β = -0.596, SE = 0.136, *p* < 0.001), receiving grooming (β = -0.594, SE = 0.148, *p* < 0.001), attention absent (β = -0.800, SE = 0.164, *p* < 0.001) and proximity (β = -2.081, SE = 0.776, *p* < 0.008).

**Supplementary Table 3. Summary of results of influence of repertoire size of homogenous and heterogeneous gestures within sequences of gestures on the duration of bonding behaviours overall and across four modalities of gestures: visual (V), tactile (T), auditory short-range (ASR) and auditory long-rage (ALR). Green squares indicate a positively significant relationship between the two variables, red squares indicate a negatively significant relationship. Blank squares indicate a relationship that was not statistically significant.**

|  | **Repertoire size of homogenous gestures** | | | | | **Repertoire size of heterogonous gestures** | | | | |
| --- | --- | --- | --- | --- | --- | --- | --- | --- | --- | --- |
|  | Overall | V | T | ASR | ALR | Overall | V | T | ASR | ALR |
| Joint feeding | + |  | - | + | - | - |  |  |  |  |
| Joint resting | + |  | + | + |  | - |  |  |  | + |
| Joint travelling | + |  | + |  |  | - |  |  |  |  |
| Giving grooming | + |  | + | + |  | - |  |  |  |  |
| Receiving grooming |  | - | - |  | - | - |  | - |  | - |
| Mutual grooming | + | + | + |  |  |  | - |  |  |  |
| Attention present | - | + | + |  |  | + | - |  |  |  |
| Attention absent | - | - |  | + | - | + |  |  |  |  |
| Proximity |  |  | + |  | - |  | - |  |  |  |

**Association between duration of time spent in social bonding behavior and presence or absence of homogenous and heterogeneous gestures**

The individuals who produced homogenous visual gesture towards dyad partner, spent longer duration of time in mutual grooming (β = -0.621, SE = 0.192, *p* = 0.001), and shorter duration of time spent receiving grooming (β = 0.712, SE = 0.244, *p* = 0.004) and attention absent (β = 0.890, SE = 0.380, *p* = 0.020). Chimpanzees that made a homogenous tactile gesture, spent longer duration of time with the dyad partner, performing following behaviour: joint resting (β = -0.680, SE = 0.234, *p* = 0.004), giving grooming (β = -0.661, SE = 0.267, *p* = 0.014), however, production of homogenous tactile gesture was associated with shorter duration of time spent receiving grooming (β = 0.696, SE = 0.236, *p* = 0.003). Production of homogenous auditory short-range gestures was associated with a longer duration of time spent in social bonding behaviours such as giving grooming (β = -1.178, SE = 0.328, *p* < 0.001) and attention absent (β = -1.071, SE = 0.500, *p* = 0.033), but shorter duration of time spent travelling (β = 0.855, SE = 0.405, *p* = 0.036). The presence of homogenous auditory long-range gestures was associated with shorter duration of time spent in five bonding behaviours: joint feeding (β = 0.628, SE = 0.269, *p* = 0.020), receiving grooming (β = 0.922, SE = 0.311, *p* = 0.003), attention present (β = 2.537, SE = 0.989, *p* = 0.011), attention absent (β = 0.935, SE = 0.338, *p* = 0.006) and proximity (β = 3.842, SE = 1.353, *p* = 0.005). The chimpanzees who communicated by producing heterogeneous visual gesture, spent shorter duration of time with the dyad partner in giving grooming (β = 0.488, SE = 0.208, *p* = 0.020), mutually grooming (β = 1.098, SE = 0.521, *p* = 0.036), attention present (β = 2.239, SE = 0.646, *p* = 0.001) and proximity (β = 2.331, SE = 0.683, *p* = 0.001). Chimpanzees that made a heterogeneous tactile gesture spent shorter duration of time receiving grooming (β = 0.861, SE = 0.336, *p* = 0.011). The heterogeneous auditory short-range gesture presence was associated with a shorter duration of time spent in attention absent (β = 1.316, SE = 0.638, *p* = 0.040).

**Supplementary Table 4. Summary of results showing GLMM of the influence of presence and absence of homogenous and heterogeneous gestures within sequences of gestures on the duration of bonding behaviours across four modalities of gestures: visual (V), tactile (T), auditory short-range (ASR) and auditory long-rage (ALR). Green squares indicate a positive value of beta coefficient, red squares indicate a negative value of beta coefficient. Blank squares indicate a relationship that was not statistically significant.**

| Independent variables | Dependent variables: duration of time spent in behaviour when within 2 meters per hour spent in the same party | | | | | | | | |
| --- | --- | --- | --- | --- | --- | --- | --- | --- | --- |
|  | Joint feeding | Joint resting | Joint travel | Groom given | Groom  received | Groom  mutual | Attention present | Attention absent | Proximity |
| Homogenous gesture absent (0) or present (1) | | | | | | | | | |
| V |  |  |  |  | + | - |  | + |  |
| T |  | - |  | - | + |  |  |  |  |
| ASR |  |  | + | - |  |  |  | - |  |
| ALR | + |  |  |  | + |  | + | + | + |
| Heterogeneous gesture absent (0) or present (1) | | | | | | | | | |
| V |  |  |  | + |  | + | + |  | + |
| T |  |  |  |  | + |  |  |  |  |
| ASR |  |  |  |  |  |  |  | + |  |
| ALR |  |  |  |  |  |  |  |  |  |

If beta coefficient is positive, the category of zero in the predictor variable is associated with higher values of the dependent variable. If beta coefficient is negative, the category of zero in the predictor variable is associated with lower values of the dependent variable.

**Dyadic repertoire size of homogenous and heterogeneous gestures and bonding behaviour**

We included repertoire size of both homogenous and heterogeneous gestures across four modalities (visual, tactile, auditory short-range and auditory long-range) in one model to examine influence of these variables on bonding behavior.

When both homogenous and heterogeneous gestures were included in one model, chimpanzee dyads that had a larger repertoire size of homogenous tactile gestures, had a longer duration of time spent in attention present (r^2^ = 0.312, β = 0.316, *p* = 0.035) and grooming received (r^2^ = 0.178, β = 0.310, *p* = 0.026) behaviours. Chimpanzee dyads that had a larger repertoire size of heterogeneous auditory long range gestures spent longer duration of time in proximity (r^2^ = 0.260, β = 0.302, *p* = 0.032), attention present (r^2^ = 0.312, β = 0.256, *p* = 0.048) and grooming received (r^2^ = 0.178, β = 0.255, *p* = 0.034). In contrast, chimpanzee dyads that had a smaller repertoire size of homogenous auditory short range gestures spent a longer duration of time in proximity (r^2^ = 0.260, β = -0.279, *p* = 0.017), resting (r^2^ = 0.124, β = -0.284, *p* = 0.002) and attention absent (r^2^ = 0.154, β = -0.293, *p* = 0.005).

Moreover, chimpanzee dyads that had a smaller repertoire size of heterogeneous auditory short range gestures spent a longer duration of time in proximity (r^2^ = 0.260, β = -0.275, *p* = 0.011), feeding (r^2^ = 0.189, β = -0.232, *p* = 0.004), travelling (r^2^ = 0.211, β = -0.201, *p* = 0.021), attention present (r^2^ = 0.312, β = -0.320, *p* = 0.004), attention absent (r^2^ = 0.154, β = -0.154, *p* = 0.03), grooming given (r^2^ = 0.200, β = -0.191, *p* = 0.028) and grooming mutual (r^2^ = 0.216, β = -0.270, *p* = 0.006).

**Supplementary Table 5. Summary of results MRQAP Regression Models predicting dyadic repertoire size of homogenous gestures and dyadic repertoire size of heterogeneous gestures combined in one model from duration of time spent in social bonding behaviour between dyads across four modalities of gestures: visual (V), tactile (T), auditory short-range (ASR) and auditory long-rage (ALR). Green squares indicate a positively significant relationship between the two variables, red squares indicate a negatively significant relationship. Blank squares indicate a relationship that was not statistically significant.**

| Bonding behaviour | Dyadic repertoire size of homogenous gestures | | | | Dyadic repertoire size of heterogeneous gestures | | | |
| --- | --- | --- | --- | --- | --- | --- | --- | --- |
|  | V | T | ASR | ALR | V | T | ASR | ALR |
| Joint feeding |  |  |  |  |  |  | - |  |
| Joint resting |  |  | - |  |  |  |  |  |
| Joint travelling |  |  |  |  |  |  | - |  |
| Giving grooming |  |  |  |  |  |  | - |  |
| Receiving grooming |  | + |  |  |  |  |  | + |
| Mutual grooming |  |  |  |  |  |  | - |  |
| Attention present |  | + |  |  |  |  | - | + |
| Attention absent |  |  | - |  |  |  | - |  |
| Proximity |  |  | - |  |  |  | - | + |

**Network size**

The next set of analyses used node level regression to examine the relationship between bonding network centrality (variance in the extent to which individual chimpanzees received bonding behaviours off conspecifics) and homogeneity centrality (variance in extent to which individual chimpanzees varied in the overlap of gestures with conspecifics, varied in the repertoire size of homogenous gestures and varied in the repertoire size of heterogeneous gestures). In these models the bonding behaviours were the dependent variable (e.g. proximity indegree) and the predictor variables were the homogeneity centrality (based on Cohen’s Kappa), the repertoire size of homogenous gestures and the repertoire size of heterogeneous gestures. In all models the control variables were included relating to kinship, age, sex and duration of time spent within 10m of oestrous female.

Overall, centrality of homogeneity of gestures was positively related to centrality for a number of bonding behaviours: joint feeding indegree (r^2^ = 0.445, β = 0.932, *p* = 0.019), giving grooming outdegree (r^2^ = 0.891, β = 1.191, *p* = 0.005**),** attention towards outdegree (r^2^ **=** 0.432, β = 0.805, *p* = 0.047)., attention away indegree (r^2^ = 0.487, β = 0.998, *p* = 0.029) and outdegree (r^2^ = 0.487, β = 0.926, *p* = 0.036**),** and proximity outdegree (r^2^ = 0.473, β = 0.881, *p* = 0.032).

When each modality was considered separately, centrality of homogeneity for visual gestures was related to joint feeding indegree (r^2^ = 0.404, β = 0.798, *p* = 0.049), grooming given outdegree (r^2^ = 0.859, β = 1.051, *p* = 0.023), attention away outdegree (r^2^ = 0.548, β = 0.902, *p* = 0.044) and proximity outdegree (r^2^ = 0.479, β = 0.805, *p* = 0.048). Homogeneity centrality for tactile gestures was related to grooming given outdegree (r^2^ = 0.531, β = 1.213, *p* = 0.029). Homogeneity centrality for auditory short-range gestures was related to a large number of bonding behaviours: joint feeding indegree (r^2^ = 0.488, β = 1.064, *p* = 0.002), joint resting indegree (r^2^ = 0.695, β = 0.989, *p* = 0.018), joint travelling outdegree (r^2^ = 0.452, β = 0.990, *p* = 0.001), giving grooming indegree (r^2^ = 0.609, β = 1.170, *p* = 0.001), receiving grooming outdegree (r^2^ = 0.406, β = 0.929, *p* = 0.005), mutual grooming indegree (r^2^ = 0.640, β = 1.059, *p* = 0.002) and outdegree (r^2^ = 0.484, β = 1.003, *p* = 0.005), attention towards indegree (r^2^ = 0.464, β = 0.957, *p* = 0.013) and outdegree (r^2^ = 0.453, β = 0.903, *p* = 0.020), attention away indegree (r^2^ = 0.435, β = 1.017, *p* = 0.020) and outdegree (r^2^ = 0.593, β = 1.132, *p* = 0.004), and proximity indegree (r^2^ = 0.458, β = 1.044, *p* = 0.009) and outdegree (r^2^ = 0.527, β = 1.025, *p* = 0.013). Finally, homogeneity centrality for auditory long-range gestures was related to joint feeding indegree (r^2^ = 0.528, β = 1.096, *p* = 0.007), giving grooming outdegree (r^2^ = 0.901, β = 1.282, *p* = 0.002), attention away indegree (r^2^ = 0.565, β = 1.155, *p* = 0.011) and outdegree (r^2^ = 0.436, β = 0.921, *p* = 0.044), and proximity indegree (r^2^ = 0.382, β = 0.931, *p* = 0.037).

**Supplementary Table 6. Summary of results node level regressions predicting indegree (IN) and outdegree (OUT) from homogeneity centrality overall, for the four modalities separately: visual (V), tactile (T), auditory short-range (ASR) and auditory long-rage (ALR). Green squares indicate a positively significant relationship between the two variables, red squares indicate a negatively significant relationship. Blank squares indicate a relationship that was not statistically significant.**

|  | **Homogeneity centrality**  **(each modality considered separately)** | | | | |
| --- | --- | --- | --- | --- | --- |
|  | Overall | V | T | ASR | ALR |
| Joint feeding | IN | IN |  | IN | IN |
| Joint resting |  |  |  | IN |  |
| Joint travelling |  |  |  | OUT |  |
| Giving grooming | OUT | OUT | OUT | IN | OUT |
| Receiving grooming |  |  |  | OUT |  |
| Mutual grooming |  |  |  | IN/OUT |  |
| Attention towards | OUT |  |  | IN/OUT |  |
| Attention away | IN/OUT | OUT |  | IN/OUT | IN/OUT |
| Proximity | OUT | OUT |  | IN/OUT | IN |

We then examined all four modalities of homogenous gestures in one model in relation to social bonding variables in and outdegree The analyses showed that individuals who had higher centrality outdegree of mutual grooming shared larger repertoire of visual gestures with conspecifics: had higher visual gesture n degree (r^2^ = 0.592, β = 2.598, *p* = 0.025).

Then we examined association between all four modalities of heterogeneous gestures including them in one model with social bonding variables in and outdegree. The analyses showed that larger repertoire size of heterogeneous auditory short range gestures with conspecifics was associated with lower centrality of seven behaviours: joint travelling indegree (r^2^ = 0.904, β = -0.824, *p* = 0.010) and outdegree (r^2^ = 0.621, β = -0.705, *p* = 0.023), giving grooming indegree (r^2^ = 0.813, β = -0.895, *p* = 0.001), receiving grooming outdegree (r^2^ = 0.634, β = -0.727, *p* = 0.005), mutual grooming indegree (r^2^ = 0.912, β = -0.888, *p* = 0.001) and outdegree (r^2^ = 0.488, β = -0.653, *p* = 0.044), attention present indegree (r^2^ = 0.897, β = -0.808, *p* = 0.012), joint feeding indegree (r^2^ = 0.555, β = -0.703, *p* = 0.041) and proximity indegree (r^2^ = 0.800, β = -0.915, *p* = 0.010).

**Supplementary Table 7. Summary of results node level regressions predicting indegree (IN) and outdegree (OUT) from repertoire size of homogenous gestures and repertoire size of heterogeneous gestures centrality for the four modalities: visual (V), tactile (T), auditory short-range (ASR) and auditory long-rage (ALR). Homogenous and heterogeneous gestures are entered separately in the models. Green squares indicate a positively significant relationship between the two variables, red squares indicate a negatively significant relationship. Blank squares indicate a relationship that was not statistically significant.**

| Dependent variable | **Repertoire size of homogeneous gestures centrality (n degree)** | | | | **Repertoire size of heterogeneous gestures centrality (n degree)** | | | |
| --- | --- | --- | --- | --- | --- | --- | --- | --- |
|  | V | T | ASR | ALR | V | T | ASR | ALR |
| Joint feeding |  |  |  |  |  |  | IN |  |
| Joint resting |  |  |  |  |  |  |  |  |
| Joint travelling |  |  |  |  |  |  | IN & OUT |  |
| Giving grooming |  |  |  |  |  |  | IN |  |
| Receiving grooming |  |  |  |  |  |  | OUT |  |
| Mutual grooming | OUT |  |  |  |  |  | IN & OUT |  |
| Attention present |  |  |  |  |  |  | IN |  |
| Attention absent |  |  |  |  |  |  |  |  |
| Proximity |  |  |  |  |  |  | IN |  |

**Overall dyadic repertoire size of homogenous gestures and bonding behaviour**

The next set of analysis used MRQAP to examine the associations between the homogenous dyadic repertoire size (the number of gestures each dyad shares) and the bonding behaviours. For all modalities together, the overall dyadic repertoire size was positively related to the duration of grooming given (r^2^ = 0.151, β = 0.285, *p* = 0.014) and grooming received (r^2^ = 0.101, β = 0.290, *p* = 0.008).

**Demography, communication and social bonding networks**

We used Multiple Regression Quadratic Assignment Procedures (MRQAP) to examine the relationship between demography (sex difference, age difference, oestrous difference, maternal kinship) and networks for social bonding behavior (joint feeding, joint resting, joint travelling, grooming received, grooming given, grooming mutual, visually attending and not attending to the focal individual and being in close proximity within 2m, per hour spent within same party across 132 chimpanzee dyads. Age difference was positively associated with longer duration of time spent in close proximity (r^2^ = 0.093, β = 0.310, *p* = 0.002), resting jointly (r^2^ = 0.067, β = 0.264, *p* = 0.002), travelling jointly (r^2^ = 0.076, β = 0.250, *p* = 0.023), grooming given (r^2^ = 0.085, β = 0.236, *p* = 0.006), attention away (r^2^ = 0.080, β = 0.299, *p* = 0.001). Sex difference was positively associated with longer duration of time spent in close proximity (r^2^ = 0.109, β = 0.238, *p* = 0.012), grooming received (r^2^ = 0.033, β = 0.218, *p* = 0.016), mutual visual attention (r^2^ = 0.089, β = 0.227, *p* = 0.034). Moreover, oestrous difference was positively associated with longer duration of time spent jointly feeding (r^2^ = 0. 109, β = 0.214, *p* = 0.042). Finally, maternal kinship was associated with longer duration of time spent jointly feeding (r^2^ = 0. 109, β = 0.291, *p* = 0.016). However, sex difference was negatively associated with longer duration of time spent resting jointly (r^2^ = 0.067, β = - 0.213, *p* = 0.028).

Next, using MRQAP we examined the relationship between demography (sex difference, age difference, oestrous difference, maternal kinship) and communication networks (homogeneity as measured by Cohen’s Kappa coefficient, dyadic repertoire size of homogenous gestures, dyadic repertoire size of heterogeneous gestures) according to visual, tactile, auditory short-range and auditory long-range modality. Age difference was positively associated with homogeneity of auditory short-range gestures (r^2^ = 0.472, β = 0.362, *p* = 0.007), homogeneity of visual gestures (r^2^ = 0.440, β = 0.254, *p* = 0.030), dyadic repertoire size of homogenous visual gestures (r^2^ = 0.182, β = 0.296, *p* = 0.004), dyadic repertoire size of homogenous auditory short-range gestures (r^2^ = 0.236, β = 0.223, *p* = 0.020), dyadic repertoire size of homogenous auditory long-range gestures (r^2^ = 0.222, β = 0.241, *p* = 0.014). Sex difference was positively associated with homogeneity of visual gestures (r^2^ = 0.440, β = 0.470, *p* = 0.004), dyadic repertoire size of homogenous visual gestures (r^2^ = 0.182, β = 0.246, *p* = 0.048), dyadic repertoire size of homogenous auditory long-range gestures (r^2^ = 0.222, β = 0.327, *p* = 0.011). Further, sex difference was negatively associated with homogeneity of auditory short-range gestures (r^2^ = 0.472, β = - 0.424, *p* = 0.046), dyadic repertoire size of heterogonous auditory long-range gestures (r^2^ = 0.603, β = - 0.722, *p* = 0.001), dyadic repertoire size of heterogonous tactile gestures (r^2^ = 0.172, β = - 0.380, *p* = 0.008), dyadic repertoire size of heterogonous visual gestures (r^2^ = 0.435, β = - 0.640, *p* = 0.005). Finally, oestrous difference was negatively associated with homogeneity of auditory short-range gestures (r^2^ = 0.472, β = - 0.615, *p* = 0.002), dyadic repertoire size of homogenous auditory short-range gestures (r^2^ = 0.236, β = - 0.469, *p* = 0.021).

1. Roberts A.I., Roberts S.G.B. 2016 Social brain hypothesis, vocal and gesture networks of wild chimpanzees. *Frontiers in Psychology* **7**(1756). (doi:10.3389/fpsyg.2016.01756 ).

2. Roberts A.I., Roberts S.G.B. 2016 Wild chimpanzees modify modality of gestures according to the strength of social bonds and personal network size. *Scientific Reports* **6**(33864). (doi:10.1038/srep33864).
