# Supplementary material for: Convergence and divergence in gesture repertoires as an adaptive mechanism for social bonding in primates
Source: R Soc Open Sci. 2017 Nov 29;4(11):170181. doi: 10.1098/rsos.170181 (PMC5717623; doi:10.1098/rsos.170181)
Supplement: Supplementary Information 2 [file rsos170181supp2.pdf]

Convergence and divergence in gesture repertoires as an adaptive mechanism for social bonding in primates

Anna Ilona Roberts, Sam George Bradley Roberts

Royal Society Open Science

Supplementary Information 2

Social networks dataset

DEMOGRAPHY

Age difference matrix

ID	BB	HW	KT	KU	KW	ML	MS	NB	NK	RH	SQ	ZM
BB	0	0	0	0	0	0	1	0	1	0	1	0
HW	0	0	1	0	0	0	1	0	0	0	1	0
KT	0	1	0	0	0	0	1	0	0	0	1	0
KU	0	0	0	0	1	1	0	0	1	0	0	0
KW	0	0	0	1	0	0	0	0	1	0	0	0
ML	0	0	0	1	0	0	0	0	0	0	0	0
MS	1	1	1	0	0	0	0	0	0	0	1	0
NB	0	0	0	0	0	0	0	0	0	1	0	0
NK	1	0	0	1	1	0	0	0	0	0	0	0
RH	0	0	0	0	0	0	0	1	0	0	0	1
SQ	1	1	1	0	0	0	1	0	0	0	0	0
ZM	0	0	0	0	0	0	0	0	0	1	0	0

Sex difference matrix

ID	BB	HW	KT	KU	KW	ML	MS	NB	NK	RH	SQ	ZM
BB	1	1	1	0	0	0	1	0	1	0	1	0
HW	1	1	1	0	0	0	1	0	1	0	1	0
KT	1	1	1	0	0	0	1	0	1	0	1	0
KU	0	0	0	1	1	1	0	1	0	1	0	1
KW	0	0	0	1	1	1	0	1	0	1	0	1

ML	0	0	0	1	1	1	0	1	0	1	0	1
MS	1	1	1	0	0	0	1	0	1	0	1	0
NB	0	0	0	1	1	1	0	1	0	1	0	1
NK	1	1	1	0	0	0	1	0	1	0	1	0
RH	0	0	0	1	1	1	0	1	0	1	0	1
SQ	1	1	1	0	0	0	1	0	1	0	1	0
ZM	0	0	0	1	1	1	0	1	0	1	0	1

Oestrous difference matrix

ID	BB	HW	KT	KU	KW	ML	MS	NB	NK	RH	SQ	ZM
BB	0	0	0	0	0	1	0	1	0	0	0	1
HW	0	0	0	0	0	1	0	1	0	0	0	1
KT	0	0	0	0	0	1	0	1	0	0	0	1
KU	0	0	0	0	0	0	0	0	0	0	0	0
KW	0	0	0	0	0	0	0	0	0	0	0	0
ML	1	1	1	0	0	0	1	0	1	0	1	0
MS	0	0	0	0	0	1	0	1	0	0	0	1
NB	1	1	1	0	0	0	1	0	1	0	1	0
NK	0	0	0	0	0	1	0	1	0	0	0	1
RH	0	0	0	0	0	0	0	0	0	0	0	0
SQ	0	0	0	0	0	1	0	1	0	0	0	1
ZM	1	1	1	0	0	0	1	0	1	0	1	0

Maternal kinship matrix

ID	BB	HW	KT	KU	KW	ML	MS	NB	NK	RH	SQ	ZM
BB	0	0	0	0	0	0	0	0	0	0	0	0
HW	0	0	0	0	0	0	0	0	0	0	0	0
KT	0	0	0	1	0	0	0	0	0	0	0	0
KU	0	0	1	0	0	0	0	0	0	0	0	0
KW	0	0	0	0	0	0	0	0	0	0	0	0
ML	0	0	0	0	0	0	0	0	0	0	0	0
MS	0	0	0	0	0	0	0	1	0	0	0	0
NB	0	0	0	0	0	0	1	0	0	0	0	0

NK	0	0	0	0	0	0	0	0	0	1	0	0
RH	0	0	0	0	0	0	0	0	1	0	0	0
SQ	0	0	0	0	0	0	0	0	0	0	0	0
ZM	0	0	0	0	0	0	0	0	0	0	0	0

HOMOGENEITY (KAPPA VALUE)

Matrix of kappa values for all modalities of gestures combined

ID	BB	HW	KT	KU	KW	ML	MS	NB	NK	RH	SQ	ZM
BB	0	0.389	0.321	0.118	0.205	0.231	0.335	0.008	0.395	0.173	0.495	0.19
HW	0.389	0	0.329	0.048	0.162	0.227	0.321	0.054	0.442	0.161	0.416	0.106
KT	0.321	0.329	0	0.12	0.231	0.122	0.389	0.124	0.341	0.161	0.382	0.106
KU	0.118	0.048	0.12	0	0.204	0.248	0.159	0.09	0.143	0.239	0.067	0.373
KW	0.205	0.162	0.231	0.204	0	0.243	0.205	0.166	0.174	0.256	0.257	0.153
ML	0.231	0.227	0.122	0.248	0.243	0	0.077	0.115	0.201	0.178	0.107	0.194
MS	0.335	0.321	0.389	0.159	0.205	0.077	0	0.161	0.429	0.214	0.495	0.19
NB	0.008	0.054	0.124	0.09	0.166	0.115	0.161	0	0.022	0.094	0.12	0.045
NK	0.395	0.442	0.341	0.143	0.174	0.201	0.429	0.022	0	0.154	0.31	0.055
RH	0.173	0.161	0.161	0.239	0.256	0.178	0.214	0.094	0.154	0	0.329	0.279
SQ	0.495	0.416	0.382	0.067	0.257	0.107	0.495	0.12	0.31	0.329	0	0.16
ZM	0.19	0.106	0.106	0.373	0.153	0.194	0.19	0.045	0.055	0.279	0.16	0

Matrix of kappa values for auditory long range gestures

ID	BB	HW	KT	KW	ML	MS	NB	NK	RH	SQ
BB	0	0.191	0.424	0.336	0.174	0.311	-0.284	0.114	0.336	0.548
HW	0.191	0	0.329	0.158	0.078	0.671	0.05	0.89	0.158	0.394
KT	0.424	0.329	0	0.103	0.05	0.65	-0.009	0.184	0.103	0.424
KW	0.336	0.158	0.103	0	-0.075	0.128	-0.145	0.128	0.441	0.336
ML	0.174	0.078	0.05	-0.075	0	0.063	-0.086	0.063	-0.075	-0.101
MS	0.311	0.671	0.65	0.128	0.063	0	0.197	0.548	0.128	0.508
NB	-0.284	0.05	-0.009	-0.145	-0.086	0.197	0	0.019	-0.145	-0.027
NK	0.114	0.89	0.184	0.128	0.063	0.548	0.019	0	0.128	0.311
RH	0.336	0.158	0.103	0.441	-0.075	0.128	-0.145	0.128	0	0.336
SQ	0.548	0.394	0.424	0.336	-0.101	0.508	-0.027	0.311	0.336	0

Matrix of kappa values for auditory short range gestures

ID	BB	HW	KT	KW	ML	MS	NB	NK	RH	SQ
BB	0	0.222	0.364	0.589	-0.167	1	-0.167	1	0.588	0.588
HW	0.222	0	0.72	0.462	0.222	0.222	-0.296	0.222	-0.077	0.462
KT	0.364	0.72	0	0.696	0.364	0.364	-0.273	0.364	0.087	0.087
KW	0.588	0.462	0.696	0	-0.235	0.588	-0.235	0.588	0.3	0.3
ML	-0.167	0.222	0.364	-0.235	0	-0.167	-0.167	-0.167	-0.235	-0.235
MS	1	0.222	0.364	0.588	-0.167	0	-0.167	1	0.588	0.588
NB	-0.167	-0.296	-0.273	-0.235	-0.167	-0.167	0	-0.167	0.588	-0.235
NK	1	0.222	0.364	0.588	-0.167	1	-0.167	0	0.588	0.588
RH	0.588	-0.077	0.087	0.3	-0.235	0.588	0.588	0.588	0	0.3
SQ	0.588	0.462	0.087	0.3	-0.235	0.588	-0.235	0.588	0.3	0

Matrix of kappa values for visual gestures

ID	BB	HW	KT	KW	ML	MS	NB	NK	RH	SQ
BB	0	0.474	0.276	0.209	0.321	0.422	0.02	0.512	0.165	0.558
HW	0.474	0	0.359	0.257	0.375	0.379	0.097	0.395	0.191	0.51
KT	0.276	0.359	0	0.257	0.306	0.379	0.236	0.459	0.191	0.444
KW	0.209	0.257	0.257	0	0.423	0.186	0.258	0.101	0.226	0.186
ML	0.321	0.375	0.306	0.423	0	0.225	0.181	0.353	0.311	0.151
MS	0.422	0.379	0.379	0.186	0.225	0	0.151	0.353	0.232	0.529
NB	0.02	0.097	0.236	0.258	0.181	0.151	0	0.013	0.196	0.151
NK	0.512	0.395	0.459	0.101	0.353	0.353	0.013	0	0.179	0.288
RH	0.165	0.191	0.191	0.226	0.311	0.232	0.196	0.179	0	0.313
SQ	0.558	0.51	0.444	0.186	0.151	0.529	0.151	0.288	0.313	0

Matrix of kappa values for tactile gestures

ID	BB	HW	KT	KW	ML	MS	NB	NK	RH	SQ
BB	0	0.383	0.322	0.132	0.114	0.073	0.114	0.28	0.099	0.367
HW	0.383	0	0.1	-0.058	0.087	-0.043	0.087	0.303	0.147	0.228
KT	0.322	0.1	0	0.26	-0.166	0.151	0.126	0.074	0.167	0.271
KW	0.132	-0.058	0.26	0	-0.043	0.345	0.165	0.394	0.208	0.374
ML	0.114	0.087	-0.166	-0.043	0	-0.103	0.058	0.096	0.129	0.247
MS	0.073	-0.043	0.151	0.345	-0.103	0	0.212	0.329	0.216	0.37
NB	0.114	0.087	0.126	0.165	0.058	0.212	0	0.096	-0.12	0.247
NK	0.28	0.303	0.074	0.394	0.096	0.329	0.096	0	0.058	0.261
RH	0.099	0.147	0.167	0.208	0.129	0.216	-0.12	0.058	0	0.378
SQ	0.367	0.228	0.271	0.374	0.247	0.37	0.247	0.261	0.378	0

DYADIC REPERTOIRE SIZE OF HOMOGENENOUS GESTURE NETWORKS

Matrix of dyadic repertoire size of homogeneous visual gestures

ID	BB	HW	KT	KU	KW	ML	MS	NB	NK	RH	SQ	ZM
BB	0	16	13	5	9	9	14	5	17	4	16	6
HW	16	0	16	4	11	11	15	7	17	5	17	7
KT	13	16	0	6	11	10	15	9	18	5	16	5
KU	5	4	6	0	4	5	5	2	6	3	4	3
KW	9	11	11	4	0	9	9	7	9	4	9	4
ML	9	11	10	5	9	0	8	5	11	4	7	5
MS	14	15	15	5	9	8	0	7	15	5	16	6
NB	5	7	9	2	7	5	7	0	6	3	7	2
NK	17	17	18	6	9	11	15	6	0	5	14	5
RH	4	5	5	3	4	4	5	3	5	0	6	3
SQ	16	17	16	4	9	7	16	7	14	6	0	5
ZM	6	7	5	3	4	5	6	2	5	3	5	0

Matrix of dyadic repertoire size of homogeneous tactile gestures

ID	BB	HW	KT	KU	KW	ML	MS	NB	NK	RH	SQ	ZM
BB	0	11	10	1	4	5	7	5	8	2	7	1
HW	11	0	7	1	2	4	5	4	7	2	5	0
KT	10	7	0	1	4	2	6	4	5	2	5	1
KU	1	1	1	0	1	0	2	1	1	0	0	1
KW	4	2	4	1	0	1	4	2	4	1	3	1
ML	5	4	2	0	1	0	2	2	3	1	3	0
MS	7	5	6	2	4	2	0	4	6	2	5	2
NB	5	4	4	1	2	2	4	0	3	0	3	1
NK	8	7	5	1	4	3	6	3	0	1	4	0
RH	2	2	2	0	1	1	2	0	1	0	2	0
SQ	7	5	5	0	3	3	5	3	4	2	0	1
ZM	1	0	1	1	1	0	2	1	0	1	1	0

Matrix of dyadic repertoire size of homogeneous auditory short-range gestures

ID	BB	HW	KT	KU	KW	ML	MS	NB	NK	RH	SQ	ZM
BB	0	1	1	0	1	0	1	0	1	1	1	0
HW	1	0	1	0	1	1	1	0	1	1	2	0
KT	1	1	0	0	2	1	1	0	1	1	1	0
KU	0	0	0	0	0	0	0	0	0	0	0	0
KW	1	1	2	0	0	0	1	0	1	1	1	0
ML	0	1	1	0	0	0	0	0	0	0	0	0
MS	1	1	1	0	1	0	0	0	1	1	1	0
NB	0	0	0	0	0	0	0	0	0	1	0	0
NK	1	1	1	0	1	0	1	0	0	1	1	0
RH	1	1	1	0	1	0	1	1	1	0	1	0

SQ	1	2	1	0	1	0	1	0	1	1	0	0
ZM	0	0	0	0	0	0	0	0	0	0	0	0

Matrix of dyadic repertoire size of homogeneous auditory long-range gestures

ID	BB	HW	KT	KU	KW	ML	MS	NB	NK	RH	SQ	ZM
BB	0	5	7	0	2	1	6	0	5	2	5	2
HW	5	0	9	0	2	1	10	2	11	2	6	1
KT	7	9	0	0	2	1	11	2	9	2	7	2
KU	0	0	0	0	0	0	0	0	0	0	0	0
KW	2	2	2	0	0	0	2	0	2	1	2	0
ML	1	1	1	0	0	0	1	0	1	0	0	0
MS	6	10	11	0	2	1	0	3	10	2	7	0
NB	0	2	2	0	0	0	3	0	2	0	1	0
NK	5	11	9	0	2	1	10	2	0	2	6	1
RH	2	2	2	0	1	0	2	0	2	0	2	1
SQ	5	6	7	0	2	0	7	1	6	2	0	1
ZM	2	1	2	0	0	0	0	0	1	1	1	0

DYADIC REPERTOIRE SIZE OF HETEROGENEOUS GESTURE NETWORKS

Matrix of dyadic repertoire size of heterogeneous visual gestures

ID	BB	HW	KT	KU	KW	ML	MS	NB	NK	RH	SQ	ZM
BB	0	16	22	18	22	18	17	26	15	20	13	17
HW	16	0	20	24	22	18	19	26	19	22	15	19
KT	22	20	0	20	22	20	19	22	17	22	17	23
KU	18	24	20	0	16	10	19	16	21	6	21	7
KW	22	22	22	16	0	14	23	18	27	16	23	17
ML	18	18	20	10	14	0	21	18	19	12	23	11
MS	17	19	19	19	23	21	0	23	20	19	14	18
NB	26	26	22	16	18	18	23	0	29	14	23	17
NK	15	19	17	21	27	19	20	29	0	23	22	24
RH	20	22	22	6	16	12	19	14	23	0	17	7
SQ	13	15	17	21	23	23	14	23	22	17	0	20
ZM	17	19	23	7	17	11	18	17	24	7	20	0

Matrix of dyadic repertoire size of heterogeneous tactile gestures

ID	BB	HW	KT	KU	KW	ML	MS	NB	NK	RH	SQ	ZM
BB	0	9	10	17	14	14	14	14	11	15	10	17
HW	9	0	13	14	15	13	15	13	10	12	11	16
KT	10	13	0	13	10	16	12	12	13	11	10	13
KU	17	14	13	0	5	9	9	7	10	4	9	2
KW	14	15	10	5	0	4	8	8	7	5	6	5

ML	14	13	16	9	4	0	5	10	11	7	8	9
MS	14	15	12	9	8	5	0	10	9	9	8	9
NB	14	13	12	7	8	10	10	0	11	9	8	7
NK	11	10	13	10	7	11	9	11	0	10	9	12
RH	15	12	11	4	5	7	9	9	10	0	5	4
SQ	10	11	10	9	6	8	8	8	9	5	0	7
ZM	17	16	13	2	5	9	9	7	12	4	7	0

Matrix of dyadic repertoire size of heterogeneous auditory short-range gestures

ID	BB	HW	KT	KU	KW	ML	MS	NB	NK	RH	SQ	ZM
BB	0	3	2	1	1	2	0	1	0	1	1	1
HW	3	0	2	4	2	3	3	5	3	4	2	4
KT	2	2	0	3	1	2	2	4	2	3	3	3
KU	1	4	3	0	2	1	1	1	1	2	2	0
KW	1	2	1	2	0	3	1	3	1	2	2	2
ML	2	3	2	1	3	0	2	2	2	3	3	1
MS	0	3	2	1	1	2	0	2	0	1	2	1
NB	1	5	4	1	3	2	2	0	2	1	3	1
NK	0	3	2	1	1	2	0	2	0	1	1	1
RH	1	4	3	2	2	3	1	1	1	0	2	2
SQ	1	2	3	2	2	3	2	3	1	2	0	2
ZM	1	4	3	0	2	1	1	1	1	2	2	0

Matrix of dyadic repertoire size of heterogeneous auditory long-range gestures

ID	BB	HW	KT	KU	KW	ML	MS	NB	NK	RH	SQ	ZM
BB	0	8	6	7	5	6	7	10	9	5	4	5
HW	8	0	6	11	9	10	3	10	1	9	6	11
KT	6	6	0	13	11	12	3	12	7	11	6	11
KU	7	11	13	0	2	1	12	3	12	2	7	2
KW	5	9	11	2	0	3	9	3	10	2	5	2
ML	6	10	12	1	3	0	11	4	11	3	8	3
MS	7	3	3	12	9	11	0	9	2	10	5	11
NB	10	10	12	3	3	4	9	0	11	2	8	5
NK	9	1	7	12	10	11	2	11	0	10	7	12
RH	5	9	11	2	2	3	10	2	10	0	5	2
SQ	4	6	6	7	5	8	5	8	7	5	0	7
ZM	5	11	11	2	2	3	11	5	12	2	7	0

RATES OF GESTURES

Matrix of rates of visual gestures un-overlapping with other modalities

ID	BB	HW	KT	KU	KW	ML	MS	NB	NK	RH	SQ	ZM

BB	0	2.222222	0	0	6	0	2.608696	3	4.426229	0	10	0
HW	3.829787	0	0	0	0	7.317073	3.75	0	5.921052	0	1.071429	1.935484
KT	5.294117	0.361446	0	0	25	0	1.935484	0	3.050848	0	0	1.237113
KU	0	0	0	0	0	0	0	0	3.050848	0	0	0
KW	6.521739	0	0	0	0	7.5	0	0	2.727273	0	0	0
ML	2.608696	1.525424	1.142857	0	0	0	1.621622	0	2.394366	0	1.052632	0
MS	0.697674	0	1.714286	0	0	1.363636	0	0	3.69863	0	0	0
NB	0	0	0	0	0	0	0	0	1.818182	0	0	0
NK	42.5	0	0.681818	0	1.666667	7.5	4.029851	0	0	0	5.384615	0
RH	0	0	0	0	0	0.517241	1.875	0	1.304348	0	0	0
SQ	5	0	0	0	0	0.285714	0	0	3	0	0	0
ZM	0	1.111111	0	0	0	0	0	0	3.333333	0	0	0

Matrix of rates of tactile gestures un-overlapping with other modalities

ID	BB	HW	KT	KU	KW	ML	MS	NB	NK	RH	SQ	ZM
BB	0	0	0	0	0	0	0	2	0.983607	0	0	0
HW	1.276596	0	0	0	0	0	1.875	0	0	0	0	0
KT	21.17647	0	0	0	0	0	0	0	0.762712	0	0	0
KU	0	0	0	0	0	0	0	0	0	0	0	0
KW	0	0	0	0	0	0	0	0	0	0	0	0
ML	0.652174	0	0	0	0	0	0	0	0.140845	0	0	0
MS	0	0	0	0	0	0	0	0	2.876712	0	0	0
NB	0	0	0	0	0	0	0	0	2.727273	0	0	0
NK	10	0	0	0	0	0.288462	0	0	0	0	0	0
RH	0	0	0	0	0	0	0	0	0.217391	0	0	0
SQ	0	0	0	0	0	0	0	0	0.428571	0	0	0
ZM	0	0	0	0	0	0	0	0	0	0	0	0

Matrix of rates of auditory short-range gestures un-overlapping with other modalities

ID	BB	HW	KT	KU	KW	ML	MS	NB	NK	RH	SQ	ZM
BB	0	0	0	0	0	0	10.86957	0	0	0	0	0
HW	0	0	0	0	0	0.731707	23.125	0	0	0	0	0.483871
KT	3.529412	0	0	0	0	0	0.967742	0	0	0	0	0
KU	0	0	0	0	0	0	0	0	0	0	0	0
KW	0	0	0	0	0	0	0	0	0	0	0	0
ML	0	0	0	0	0	0	0	0	0	0	0	0
MS	6.27907	0	0	0	0	0	0	0	0.410959	0	0	0
NB	0	0	0	0	0	0	0	0	0	0	0	0
NK	5	0	0	0	0	0	0	0	0	0	0.769231	0
RH	0	0.447761	0	0	0	0	0	0	0.434783	0	0	0
SQ	0	0	0	0	0	0.285714	0	0	0.428571	0	0	0
ZM	0	0	0	0	0	0	0	0	0	0	0	0

Matrix of rates of auditory long-range gestures un-overlapping with other modalities

ID	BB	HW	KT	KU	KW	ML	MS	NB	NK	RH	SQ	ZM
BB	0	0	0	0	2	0	0	1	0.983607	0	10	0
HW	0.638298	0	0	0	0	1.463415	0.625	0	0	0	0	1.451613
KT	0	0	0	0	15	0	0.967742	0	0	0	0	1.237113
KU	0	0	0	0	0	0	0	0	0	0	0	0
KW	0	0	0	0	0	7.5	0	0	0	0	0	0
ML	0	0	0	0	0	0	0	0	0	0	0	0
MS	0	0	0.857143	0	0	1.363636	0	0	0	0	0	0
NB	0	0	0	0	0	0	0	0	0	0	0	0
NK	0	0	1.363636	0	1.666667	2.019231	0.447761	0	0	0	0.769231	0
RH	0	0	0	0	0	0	0	0	0	0	0	0
SQ	1.666667	0	0	0	0	0	0	0	0	0	0	0
ZM	0	0	0	0	0	0	0	0	0	0	0	0

SOCIAL BONDING NETWORKS

Matrix of joint feeding

ID	BB	HW	KT	KU	KW	ML	MS	NB	NK	RH	SQ	ZM
BB	0	0	0	0	2.727273	0	4.660194	0	0	0	0	0
HW	0.487805	0	0.674157	0	0	0.48	0.902256	0.731707	0	0	0	1.875
KT	0	1.565217	0	0	0	0	0.276498	0	0	0	0	0.659341
KU	0	0	1.607143	0	2.432432	0	0	0	1.978022	0	0	0
KW	0	0	0	0	0	0	0	0	0	0	0	0
ML	0	2.362205	1.218274	0	0	0	0.582524	1.132075	1.632653	0	0.262009	0
MS	7.384615	0	0	0	0	0	0	0	1.188119	0	0	0
NB	3	0	0	0	0	3.529412	8.25	0	0	0	0	1.714286
NK	0	0	0	0	0	2.238806	5.769231	0	0	0	2.068965	0
RH	0	0	0	0	0	3.846154	0	0	3	0	0	0
SQ	0	0.517241	1.121495	0	0	2.195122	0.84507	0	1.860465	0	0	0
ZM	0	0.779221	0.652174	1.463415	0	0	0	1.290323	1.47541	0	0	0

Matrix of joint resting

ID	BB	HW	KT	KU	KW	ML	MS	NB	NK	RH	SQ	ZM
BB	0	1.666667	0	0	2.727273	0	4.07767	1.578947	1.2	0	0	0
HW	2.439024	0	0.337079	0	0	0	4.511278	0	1.256544	0	0	0.46875
KT	0	1.304348	0	0	0	0	0.552995	0	1.565217	0	0	0
KU	0	0	0	0	0.810811	0	0	0.689655	0.659341	1.714286	0	0
KW	0	0	0	0	0	0	0	0	0	0	0	0
ML	1.804511	0.708661	0	4.285714	0	0	0	0	6.734694	0	0.524017	0
MS	0.923077	0	0	0	0	0	0	0	2.376238	0	0	0
NB	3	0	0	0	0	0	0	0	1.176471	0	0	0
NK	4	0	0	60	0	2.686567	0.576923	0	0	0	0	0
RH	0	0	0	0	0	3.076923	0	0.618557	3.3	0	0	0
SQ	0	4.655172	0	0	0	0.365854	0	0	0.465116	0	0	0

ZM	0	0	1.304348	0	0.9375	0	0	0	0	0	0	0

Matrix of joint travelling

ID	BB	HW	KT	KU	KW	ML	MS	NB	NK	RH	SQ	ZM
BB	0	1.666667	0	0	2.727273	0	0	0	3	0	0	0
HW	0	0	0	0	0	0	0.902256	0	0	0	0	1.40625
KT	0	0.26087	0	0	0	0	0	0	0	0	0	0
KU	0	0	0.535714	0	0	0	0	0	0	0	0	0
KW	0	0	0	0	0	0	0	0	0	0	0	0
ML	0	0	0	4.285714	0	0	0	0	0.816327	0	0	0
MS	1.846154	0	0.9375	0	0	0	0	0	0.594059	0	0	0
NB	0	0	0	0	0	0	0	0	0	0	0	0
NK	8	0	1.978022	0	1.304348	1.343284	0.576923	0	0	0	0	0
RH	0	0	0	0	0	0	0	0.618557	0	0	0	0
SQ	0	0	0	0	0	0	0	0	0	0	0	0
ZM	0	0	0	0	0	0	0	0	0	0	0	0

Matrix of giving grooming

ID	BB	HW	KT	KU	KW	ML	MS	NB	NK	RH	SQ	ZM
BB	0	0	0	0	0	0	2.330097	0.789474	0	0	0	0
HW	0.97561	0	0	0	0	0	6.766917	0	0	0	0	0.46875
KT	2.647059	0	0	0	0	0	0.829493	0	0.26087	0	0	0
KU	0	0	0	0	0	0	0	0	0	0	0	0
KW	0	0	0	0	0	0	0	0	0	0	0	0
ML	0	0.23622	0	0	0	0	1.165049	0	0.816327	0	0	0
MS	6.461538	0	0	0	0	0	0	0	2.970297	0	0	0
NB	0	0	0	0	0	0	0.75	0	0	0	0	0
NK	4	0	0	0	2.608696	2.238806	0	0	0	0	0	0
RH	0	0	0	0	0	0	0	0	4.2	0	0	0
SQ	0	0	0	0	0	0.731707	2.535211	0	2.325581	0	0	0

ZM	0	0	0	0	0	0	0	0	0	0	0	0

Matrix of mutual grooming

ID	BB	HW	KT	KU	KW	ML	MS	NB	NK	RH	SQ	ZM
BB	0	0	0	0	0	0	4.07767	0.789474	5.4	0	0	0
HW	0	0	0	0	0	0	0	0	0	0	0	0
KT	0	0	0	0	0	0	0	0	0	0	0	0
KU	0	0	0	0	0	0	0	0	0	0	0	0
KW	0	0	0	0	0	0	0	0	0	0	0	0
ML	2.706767	0	0	0	0	0	5.242718	0	0.204082	0	0.786026	0
MS	0.923077	0	0	0	0	0	0	0	1.782178	0	0	0
NB	0	0	0	0	0	0	0	0	0	0	0	0
NK	16	0	0	0	2.608696	0.447761	0	0	0	0	1.034483	0
RH	0	0	0	0	0	0	0	0	2.4	0	0	0
SQ	0	0	0	0	0	2.926829	0	0	2.325581	0	0	0
ZM	0	0	0	0	0	0	0	0	0	0	0	0

Matrix of receiving grooming

ID	BB	HW	KT	KU	KW	ML	MS	NB	NK	RH	SQ	ZM
BB	0	11.66667	0	0	0	0	1.747573	0	0	0	0	0
HW	0	0	0	0	0	0	0	0	0	0	0	0
KT	0	0	0	0	0	0	0	0	0	0	0	0
KU	0	0	0	0	0.405405	0	0	0	1.318681	0	0	0
KW	0	0	0	0	0	0	0	0	0	0	0	0
ML	0.902256	0.472441	0	0	0	0	0	0	0.816327	0	0	0
MS	1.846154	0	0	0	0	0	0	0	0	0	0	0
NB	0	0	0	0	0	0	0	0	0	0	0	0
NK	4	0	0	0	0	1.343284	6.346154	3.076923	0	0	7.241379	0
RH	0	0	0	0	0	0.769231	0	0	0.9	0	0	0
SQ	0	0	0	0	0	0	0	0	0	0	0	0
ZM	0	0	0	0	0	0	0	0	0.491803	0	0	0

Matrix of attention towards

ID	BB	HW	KT	KU	KW	ML	MS	NB	NK	RH	SQ	ZM
BB	0	3.333333	0	0	2.727273	0	14.56311	3.157895	6.6	0	0	0
HW	3.902439	0	1.685393	0	0	0.48	3.609023	0	1.256544	0	0	1.875
KT	1.764706	1.304348	0	0	0	0	0.552995	0	1.565217	0	0	0.659341
KU	0	0	0	0	2.432432	0	0	0.689655	0.659341	1.714286	0	0
KW	0	0	0	0	0	0	0	0	1.682243	0	0	0
ML	5.413534	1.653543	1.218274	0	0	0	5.242718	1.132075	5.510204	0	1.310044	0
MS	12.92308	0	0	0	0	0	0	4.090909	4.158416	0	0	0
NB	3	0	0	0	0	1.764706	7.5	0	2.352941	0	0	0
NK	32	0	0.659341	0	0	4.477612	6.346154	6.153846	0	0	5.172414	0
RH	0	0	0	0	0	7.692307	0	0	9.3	0	0	0
SQ	0	1.034483	1.121495	0	0	5.121951	3.380282	0	5.581395	0	0	0
ZM	0	0	0.652174	1.463415	0.9375	0	0	1.290323	2.459016	0	0	0

Matrix of attention away

ID	BB	HW	KT	KU	KW	ML	MS	NB	NK	RH	SQ	ZM
BB	0	11.66667	0	0	8.181818	0	5.825243	0	3.6	0	0	0
HW	2.439024	0	0.674157	0	0	0	10.37594	0.731707	0	0	0	2.34375
KT	1.764706	4.434783	0	0	0	0	2.764977	0	0.782609	0	0	0
KU	0	0	2.142857	0	2.027027	0	0	0	3.296703	0	0	0
KW	0	1.5	0	0	0	0	0	0	1.121495	0	0	0
ML	1.804511	2.362205	1.218274	8.571428	0	0	2.330097	0	6.32653	0	0.262009	0
MS	8.307693	0	0.9375	0	0	2.857143	0	0	5.940594	0	4.137931	0
NB	3	0	0	0	0	1.764706	3	0	0	0	0	1.714286
NK	8	0	1.978022	60	1.304348	6.268657	8.076923	0	0	0	5.172414	0
RH	0	0	0	0	0	5.384615	0	1.237113	10.2	0	1.714286	0

SQ	0	4.655172	0	0	0	1.097561	2.535211	0	1.395349	0	0	0
ZM	0	0.779221	1.304348	0	0	0	0	0	1.967213	0	0	0

Matrix of proximity

ID	BB	HW	KT	KU	KW	ML	MS	NB	NK	RH	SQ	ZM
BB	0	15	0	0	10.90909	0	20.38835	3.157895	10.2	0	0	0
HW	6.341464	0	2.35955	0	0	0.48	13.98496	0.731707	1.256544	0	0	4.21875
KT	3.529412	5.73913	0	0	0	0	3.317972	0	2.347826	0	0	0.659341
KU	0	0	2.142857	0	4.459459	0	0	0.689655	3.956044	1.714286	0	0
KW	0	1.5	0	0	0	0	0	0	2.803738	0	0	0
ML	7.218045	4.015748	2.436548	8.571428	0	0	7.572815	1.132075	11.83673	0	1.572052	0
MS	21.23077	0	0.9375	0	0	2.857143	0	4.090909	10.09901	0	4.137931	0
NB	6	0	0	0	0	3.529412	10.5	0	2.352941	0	0	1.714286
NK	40	0	2.637363	60	1.304348	10.74627	14.42308	6.153846	0	0	10.34483	0
RH	0	0	0	0	0	13.07692	0	1.237113	19.5	0	1.714286	0
SQ	0	5.689655	1.121495	0	0	6.219512	5.915493	0	6.976744	0	0	0
ZM	0	0.779221	1.956522	1.463415	0.9375	0	0	1.290323	4.426229	0	0	0

CENTRALITIES DEMOGRAPHY

ID	Duration focal subject is in oestrous within 10 meters of the dyad partner per hour spent in same party outdegree - excludes kin	Kinship duration outdegree	Age/sex combined
BB	0	0	2
HW	0	0	4
KT	0	0	4
KU	0	2.338	3
KW	0	0	3
ML	21.663	0	3
MS	0	2.479	2
NB	6.335	2.932	1
NK	0	2.182	2

RH	0	3.764	1
SQ	0	0	2
ZM	4.883	0	1
