# Supplementary material for: Convergence and divergence in gesture repertoires as an adaptive mechanism for social bonding in primates
Source: R Soc Open Sci. 2017 Nov 29;4(11):170181. doi: 10.1098/rsos.170181 (PMC5717623; doi:10.1098/rsos.170181)
Supplement: Supplementary Information 3 [file rsos170181supp3.pdf]

Convergence and divergence in gesture repertoires as an adaptive mechanism for social bonding in primates

Anna Ilona Roberts, Sam George Bradley Roberts

Royal Society Open Science

Supplementary Information 3

GLMM models dataset

matching number	Focal subject/ signaller	Recipient	Sex difference	Age difference	Oestrous difference	Maternal kinship
1.00	Zimba	nick	0.00	0.00	1.00	0.00
2.00	Hawa	nick	1.00	0.00	0.00	0.00
3.00	Nick	kalema	0.00	1.00	1.00	0.00
4.00	Hawa	musa	1.00	1.00	0.00	0.00
5.00	Hawa	zimba	0.00	0.00	1.00	0.00
6.00	Hawa	musa	1.00	1.00	0.00	0.00
7.00	Hawa	musa	1.00	1.00	0.00	0.00
8.00	Hawa	zefa	1.00	0.00	0.00	0.00
9.00	Kato	musa	1.00	1.00	0.00	0.00
10.00	Hawa	melissa	0.00	0.00	1.00	0.00
11.00	Hawa	musa	1.00	1.00	0.00	0.00
12.00	Melissa	nick	0.00	0.00	1.00	0.00
13.00	Hawa	nick	1.00	0.00	0.00	0.00
14.00	Kato	bwoba	1.00	0.00	0.00	0.00
15.00	Musa	nick	1.00	0.00	0.00	0.00
16.00	Hawa	musa	1.00	1.00	0.00	0.00
17.00	Hawa	zimba	0.00	0.00	0.00	0.00
18.00	Squibs	nick	1.00	0.00	0.00	0.00

19.00	Melissa	bwoba	0.00	0.00	1.00	0.00
20.00	Melissa	squibs	0.00	0.00	999.00	0.00
21.00	Kato	musa	1.00	1.00	0.00	0.00
22.00	Melissa	nick	0.00	0.00	999.00	0.00
23.00	Hawa	musa	1.00	1.00	0.00	0.00
24.00	Hawa	musa	1.00	1.00	0.00	0.00
25.00	Hawa	musa	1.00	1.00	0.00	0.00
26.00	Hawa	bwoba	1.00	0.00	0.00	0.00
27.00	Kutu	nick	0.00	1.00	999.00	0.00
28.00	Kato	nick	1.00	0.00	0.00	0.00
29.00	Ruhara	nick	0.00	0.00	0.00	1.00
30.00	Kwera	anna	1.00	0.00	0.00	0.00
31.00	Kato	zimba	0.00	0.00	1.00	0.00
32.00	Kato	zimba	0.00	0.00	1.00	0.00
33.00	Hawa	zefa	1.00	0.00	0.00	0.00
34.00	Musa	nick	1.00	0.00	0.00	0.00
35.00	Nick	bwoba	1.00	1.00	0.00	0.00
36.00	Musa	nick	1.00	0.00	0.00	0.00
37.00	Kato	nick	1.00	0.00	0.00	0.00
38.00	Kato	zefa	1.00	0.00	0.00	0.00
39.00	Musa	nick	1.00	0.00	0.00	0.00
40.00	Musa	nick	1.00	0.00	0.00	0.00
41.00	Musa	nick	1.00	0.00	0.00	0.00
42.00	Nick	kato	1.00	0.00	0.00	0.00
43.00	Bwoba	nick	1.00	1.00	0.00	0.00
44.00	Kato	hariette	0.00	0.00	1.00	0.00
45.00	Bwoba	sarine	0.00	0.00	0.00	0.00
46.00	Squibs	bwoba	1.00	1.00	0.00	0.00
47.00	Bwoba	squibs	1.00	1.00	0.00	0.00
48.00	Bwoba	sarine	0.00	0.00	0.00	0.00
49.00	Nick	bwoba	1.00	1.00	0.00	0.00
50.00	Kato	hariette	0.00	0.00	999.00	0.00

51.00	Nick	kato	1.00	0.00	0.00	0.00
52.00	Nick	tinka	1.00	0.00	0.00	0.00
53.00	Bwoba	kwera	0.00	0.00	0.00	0.00
54.00	Nick	kato	1.00	0.00	0.00	0.00
55.00	Bwoba	nick	1.00	1.00	0.00	0.00
56.00	Kato	zimba	0.00	0.00	0.00	0.00
57.00	Hawa	kato	1.00	1.00	0.00	0.00
58.00	Hawa	musa	1.00	1.00	0.00	0.00
59.00	Hawa	squibs	1.00	1.00	0.00	0.00
60.00	Hawa	nick	1.00	0.00	0.00	0.00
61.00	Hawa	zefa	1.00	0.00	0.00	0.00
62.00	Melissa	zefa	0.00	0.00	999.00	0.00
63.00	Hawa	bwoba	1.00	0.00	0.00	0.00
64.00	Hawa	nick	1.00	0.00	0.00	0.00
65.00	Nambi	nick	0.00	0.00	999.00	0.00
66.00	Kato	nick	1.00	0.00	0.00	0.00
67.00	Kwera	melissa	1.00	0.00	0.00	0.00
68.00	Zimba	tinka	0.00	0.00	0.00	0.00
69.00	Kato	nick	1.00	0.00	0.00	0.00
70.00	Kato	kwera	0.00	0.00	0.00	0.00
71.00	Hawa	nick	1.00	0.00	0.00	0.00
72.00	Hawa	nick	1.00	0.00	0.00	0.00
73.00	Hawa	bwoba	1.00	0.00	0.00	0.00
74.00	Hawa	bwoba	1.00	0.00	0.00	0.00
75.00	Bwoba	nick	1.00	1.00	0.00	0.00
76.00	Squibs	nick	1.00	0.00	0.00	0.00
77.00	Kato	nick	1.00	0.00	0.00	0.00
78.00	Kwera	nick	0.00	1.00	0.00	0.00
79.00	Nick	kwera	0.00	1.00	0.00	0.00
80.00	Hawa	tinka	1.00	0.00	0.00	0.00
81.00	Kato	nick	1.00	0.00	0.00	0.00
82.00	Kato	nick	1.00	0.00	0.00	0.00

83.00	Musa	tinka	1.00	0.00	0.00	0.00
84.00	Nambi	nick	0.00	0.00	1.00	0.00
85.00	Kato	musa	1.00	1.00	0.00	0.00
86.00	Kwera	bwoba	0.00	0.00	0.00	0.00
87.00	Kwera	bwoba	0.00	0.00	0.00	0.00
88.00	Kwera	bwoba	0.00	0.00	0.00	0.00
89.00	Kato	zefa	1.00	0.00	0.00	0.00
90.00	Melissa	nick	0.00	0.00	999.00	0.00
91.00	Hawa	bwoba	1.00	0.00	0.00	0.00
92.00	Hawa	musa	1.00	1.00	0.00	0.00
93.00	Melissa	nick	0.00	0.00	1.00	0.00
94.00	Hawa	nick	1.00	0.00	0.00	0.00
95.00	Ruhara	musa	0.00	0.00	0.00	0.00
96.00	Hawa	musa	1.00	1.00	0.00	0.00
97.00	Kato	nick	1.00	0.00	0.00	0.00
98.00	Kato	nambi	0.00	0.00	0.00	0.00
99.00	Kato	nick	1.00	0.00	0.00	0.00
100.00	Kato	bwoba	1.00	0.00	0.00	0.00
101.00	Kato	nick	1.00	0.00	0.00	0.00
102.00	Hawa	zefa	1.00	0.00	0.00	0.00
103.00	Hawa	zefa	1.00	0.00	0.00	0.00
104.00	Hawa	zefa	1.00	0.00	0.00	0.00
105.00	Nick	melissa	0.00	0.00	1.00	0.00
106.00	Squibs	kigere	0.00	0.00	1.00	0.00
107.00	Hawa	melissa	0.00	0.00	1.00	0.00
108.00	Nick	bwoba	1.00	1.00	0.00	0.00
109.00	Musa	zefa	1.00	0.00	0.00	0.00
110.00	Nick	melissa	0.00	0.00	0.00	0.00
111.00	Nick	melissa	0.00	0.00	1.00	0.00
112.00	Nick	melissa	0.00	0.00	0.00	0.00
113.00	Nick	melissa	0.00	0.00	0.00	0.00
114.00	Nick	bwoba	1.00	1.00	0.00	0.00

115.00	Nick	bwoba	1.00	1.00	0.00	0.00
116.00	Kato	tinka	1.00	0.00	0.00	0.00
117.00	Musa	melissa	0.00	0.00	1.00	0.00
118.00	Nick	squibs	1.00	0.00	0.00	0.00
119.00	Musa	kwera	0.00	0.00	0.00	0.00
120.00	Kato	tinka	1.00	0.00	0.00	0.00
121.00	Kato	tinka	1.00	0.00	0.00	0.00
122.00	Nick	bwoba	1.00	1.00	0.00	0.00
123.00	Nick	kalema	0.00	1.00	1.00	0.00
124.00	Nick	melissa	0.00	0.00	1.00	0.00
125.00	Nick	melissa	0.00	0.00	1.00	0.00
126.00	Nick	melissa	0.00	0.00	1.00	0.00
127.00	Nick	melissa	0.00	0.00	1.00	0.00
128.00	Nick	bwoba	1.00	1.00	0.00	0.00
129.00	Nick	bwoba	1.00	1.00	0.00	0.00
130.00	Nick	squibs	1.00	0.00	0.00	0.00
131.00	Nick	bwoba	1.00	1.00	0.00	0.00
132.00	Nick	melissa	0.00	0.00	0.00	0.00
133.00	Squibs	mukwano	0.00	0.00	0.00	0.00
134.00	Nick	zefa	1.00	1.00	0.00	0.00
135.00	Nick	kato	1.00	0.00	0.00	0.00
136.00	Kato	wilma	0.00	0.00	1.00	0.00
137.00	Hawa	melissa	0.00	0.00	1.00	0.00
138.00	Kato	wilma	0.00	0.00	1.00	0.00
139.00	Kato	kalema	0.00	0.00	999.00	0.00
140.00	Kato	kalema	0.00	0.00	999.00	0.00
141.00	Hawa	melissa	0.00	0.00	1.00	0.00
142.00	Kato	wilma	0.00	0.00	1.00	0.00
143.00	Kato	wilma	0.00	0.00	1.00	0.00
144.00	Kato	wilma	0.00	0.00	1.00	0.00
145.00	Kato	zimba	0.00	0.00	1.00	0.00
146.00	Hawa	melissa	0.00	0.00	1.00	0.00

147.00	Hawa	zimba	0.00	0.00	1.00	0.00
148.00	Hawa	zimba	0.00	0.00	1.00	0.00
149.00	Hawa	zimba	0.00	0.00	1.00	0.00
150.00	Nick	melissa	0.00	0.00	1.00	0.00
151.00	Kato	nick	1.00	0.00	0.00	0.00
152.00	Kutu	nick	0.00	1.00	999.00	0.00
153.00	Kutu	nick	0.00	1.00	0.00	0.00
154.00	Melissa	nick	0.00	0.00	1.00	0.00
155.00	Melissa	zefa	0.00	0.00	999.00	0.00
156.00	Melissa	nick	0.00	0.00	1.00	0.00
157.00	Kato	nick	1.00	0.00	0.00	0.00
158.00	Zimba	nick	0.00	0.00	0.00	0.00
159.00	Kutu	ruhara	1.00	0.00	0.00	0.00
160.00	Melissa	nick	0.00	0.00	0.00	0.00
161.00	Hawa	melissa	0.00	0.00	999.00	0.00
162.00	Hawa	melissa	0.00	0.00	999.00	0.00
163.00	Hawa	melissa	0.00	0.00	999.00	0.00
164.00	Hawa	zimba	0.00	0.00	1.00	0.00
165.00	Bwoba	hawa	1.00	0.00	0.00	0.00
166.00	Kato	zimba	0.00	0.00	1.00	0.00
167.00	Kato	zimba	0.00	0.00	1.00	0.00
168.00	Ruhara	hawa	0.00	0.00	0.00	0.00
169.00	Kwera	kewaya	1.00	1.00	0.00	0.00
170.00	Musa	maani	1.00	0.00	0.00	0.00
171.00	Musa	maani	1.00	0.00	0.00	0.00
172.00	Musa	maani	1.00	0.00	0.00	0.00
173.00	Musa	maani	1.00	0.00	0.00	0.00
174.00	Musa	maani	1.00	0.00	0.00	0.00
175.00	Kato	hariette	0.00	0.00	1.00	0.00
176.00	Kato	hariette	0.00	0.00	1.00	0.00
177.00	Bwoba	musa	1.00	1.00	0.00	0.00
178.00	Bwoba	musa	1.00	1.00	0.00	0.00

179.00	Musa	nick	1.00	0.00	0.00	0.00
180.00	Musa	nick	1.00	0.00	0.00	0.00
181.00	Musa	nick	1.00	0.00	0.00	0.00
182.00	Musa	nick	1.00	0.00	0.00	0.00
183.00	Musa	nick	1.00	0.00	0.00	0.00
184.00	Nick	tinka	1.00	0.00	0.00	0.00
185.00	Nick	tinka	1.00	0.00	0.00	0.00
186.00	Nick	kwera	0.00	1.00	999.00	0.00
187.00	Zimba	nick	0.00	0.00	1.00	0.00
188.00	Zimba	nick	0.00	0.00	1.00	0.00
189.00	Nick	musa	1.00	0.00	0.00	0.00
190.00	Nick	musa	1.00	0.00	0.00	0.00
191.00	Nick	musa	1.00	0.00	0.00	0.00
192.00	Nick	musa	1.00	0.00	0.00	0.00
193.00	Nick	kalema	0.00	1.00	1.00	0.00
194.00	Nick	kalema	0.00	1.00	1.00	0.00
195.00	Nick	melissa	0.00	0.00	999.00	0.00
196.00	Nick	bwoba	1.00	1.00	0.00	0.00
197.00	Nick	bwoba	1.00	1.00	0.00	0.00
198.00	Nick	bwoba	1.00	1.00	0.00	0.00
199.00	Nick	bwoba	1.00	1.00	0.00	0.00
200.00	Nick	bwoba	1.00	1.00	0.00	0.00
201.00	Nick	bwoba	1.00	1.00	0.00	0.00
202.00	Nick	bwoba	1.00	1.00	0.00	0.00
203.00	Musa	bwoba	1.00	1.00	0.00	0.00
204.00	Musa	bwoba	1.00	1.00	0.00	0.00
205.00	Musa	bwoba	1.00	1.00	0.00	0.00
206.00	Musa	bwoba	1.00	1.00	0.00	0.00
207.00	Musa	bwoba	1.00	1.00	0.00	0.00
208.00	Musa	bwoba	1.00	1.00	0.00	0.00
209.00	Musa	bwoba	1.00	1.00	0.00	0.00
210.00	Musa	bwoba	1.00	1.00	0.00	0.00

211.00	Musa	bwoba	1.00	1.00	0.00	0.00
212.00	Musa	bwoba	1.00	1.00	0.00	0.00
213.00	Musa	bwoba	1.00	1.00	0.00	0.00
214.00	Musa	bwoba	1.00	1.00	0.00	0.00
215.00	Bwoba	musa	1.00	1.00	0.00	0.00
216.00	Bwoba	musa	1.00	1.00	0.00	0.00
217.00	Bwoba	musa	1.00	1.00	0.00	0.00
218.00	Bwoba	musa	1.00	1.00	0.00	0.00
219.00	Bwoba	musa	1.00	1.00	0.00	0.00
220.00	Bwoba	musa	1.00	1.00	0.00	0.00
221.00	Bwoba	musa	1.00	1.00	0.00	0.00
222.00	Bwoba	musa	1.00	1.00	0.00	0.00
223.00	Bwoba	musa	1.00	1.00	0.00	0.00
224.00	Bwoba	musa	1.00	1.00	0.00	0.00
225.00	Bwoba	musa	1.00	1.00	0.00	0.00
226.00	Bwoba	musa	1.00	1.00	0.00	0.00
227.00	Bwoba	musa	1.00	1.00	0.00	0.00
228.00	Bwoba	musa	1.00	1.00	0.00	0.00
229.00	Bwoba	musa	1.00	1.00	0.00	0.00
230.00	Bwoba	musa	1.00	1.00	0.00	0.00
231.00	Bwoba	musa	1.00	1.00	0.00	0.00
232.00	Bwoba	musa	1.00	1.00	0.00	0.00
233.00	Bwoba	musa	1.00	1.00	0.00	0.00
234.00	Bwoba	musa	1.00	1.00	0.00	0.00
235.00	Bwoba	musa	1.00	1.00	0.00	0.00
236.00	Bwoba	musa	1.00	1.00	0.00	0.00
237.00	Bwoba	musa	1.00	1.00	0.00	0.00
238.00	Bwoba	musa	1.00	1.00	0.00	0.00
239.00	Bwoba	musa	1.00	1.00	0.00	0.00
240.00	Bwoba	musa	1.00	1.00	0.00	0.00
241.00	Bwoba	musa	1.00	1.00	0.00	0.00
242.00	Bwoba	musa	1.00	1.00	0.00	0.00

243.00	Musa	bwoba	1.00	1.00	0.00	0.00
244.00	Squibs	nick	1.00	0.00	0.00	0.00
245.00	Nick	melissa	0.00	0.00	1.00	0.00
246.00	Nick	squibs	1.00	0.00	0.00	0.00
247.00	Nick	squibs	1.00	0.00	0.00	0.00
248.00	Nick	squibs	1.00	0.00	0.00	0.00
249.00	Nick	squibs	1.00	0.00	0.00	0.00
250.00	Squibs	nick	1.00	0.00	0.00	0.00
251.00	Kato	musa	1.00	1.00	0.00	0.00
252.00	Nick	musa	1.00	0.00	0.00	0.00
253.00	Nick	musa	1.00	0.00	0.00	0.00
254.00	Nick	musa	1.00	0.00	0.00	0.00
255.00	Nick	musa	1.00	0.00	0.00	0.00
256.00	Nambi	nick	0.00	0.00	0.00	0.00
257.00	Musa	kato	1.00	1.00	0.00	0.00
258.00	Bwoba	mukwano	0.00	0.00	0.00	0.00
259.00	Bwoba	mukwano	0.00	0.00	0.00	0.00
260.00	Bwoba	mukwano	0.00	0.00	0.00	0.00
261.00	Bwoba	mukwano	0.00	0.00	0.00	0.00
262.00	Bwoba	mukwano	0.00	0.00	0.00	0.00
263.00	Bwoba	mukwano	0.00	0.00	0.00	0.00
264.00	Nick	melissa	0.00	0.00	0.00	0.00
265.00	Kutu	nick	0.00	1.00	0.00	0.00
266.00	Ruhara	nick	0.00	0.00	0.00	1.00
267.00	Hawa	musa	1.00	1.00	0.00	0.00
268.00	Hawa	musa	1.00	1.00	0.00	0.00
269.00	Hawa	musa	1.00	1.00	0.00	0.00
270.00	Hawa	musa	1.00	1.00	0.00	0.00
271.00	Hawa	musa	1.00	1.00	0.00	0.00
272.00	Hawa	musa	1.00	1.00	0.00	0.00
273.00	Hawa	musa	1.00	1.00	0.00	0.00
274.00	Hawa	musa	1.00	1.00	0.00	0.00

275.00	Hawa	musa	1.00	1.00	0.00	0.00
276.00	Hawa	musa	1.00	1.00	0.00	0.00
277.00	Hawa	musa	1.00	1.00	0.00	0.00
278.00	Squibs	nick	1.00	0.00	0.00	0.00
279.00	Squibs	nick	1.00	0.00	0.00	0.00
280.00	Nick	squibs	1.00	0.00	0.00	0.00
281.00	Kutu	kwera	1.00	1.00	999.00	0.00
282.00	Hawa	hariette	0.00	0.00	0.00	1.00
283.00	Hawa	hariette	0.00	0.00	999.00	1.00
284.00	Bwoba	nambi	0.00	0.00	0.00	0.00
285.00	Bwoba	nambi	0.00	0.00	0.00	0.00
286.00	Bwoba	nambi	0.00	0.00	0.00	0.00
287.00	Ruhara	nick	0.00	0.00	0.00	1.00
288.00	Melissa	simon	0.00	0.00	1.00	0.00
289.00	Melissa	simon	0.00	0.00	1.00	0.00
290.00	Melissa	simon	0.00	0.00	1.00	0.00
291.00	Melissa	simon	0.00	0.00	999.00	0.00
292.00	Melissa	zefa	0.00	0.00	1.00	0.00
293.00	Melissa	hawa	0.00	0.00	1.00	0.00
294.00	Melissa	zefa	0.00	0.00	999.00	0.00
295.00	Melissa	nick	0.00	0.00	999.00	0.00
296.00	Melissa	bwoba	0.00	0.00	1.00	0.00
297.00	Melissa	squibs	0.00	0.00	1.00	0.00
298.00	Melissa	squibs	0.00	0.00	1.00	0.00
299.00	Bwoba	nick	1.00	1.00	0.00	0.00
300.00	Melissa	kato	0.00	0.00	1.00	0.00
301.00	Melissa	hawa	0.00	0.00	1.00	0.00
302.00	Melissa	bwoba	0.00	0.00	1.00	0.00
303.00	Melissa	bwoba	0.00	0.00	1.00	0.00
304.00	Hawa	melissa	0.00	0.00	1.00	0.00
305.00	Melissa	hawa	0.00	0.00	999.00	0.00
306.00	Melissa	nick	0.00	0.00	1.00	0.00

307.00	Melissa	nick	0.00	0.00	1.00	0.00
308.00	Melissa	musa	0.00	0.00	1.00	0.00
309.00	Melissa	musa	0.00	0.00	1.00	0.00
310.00	Melissa	musa	0.00	0.00	999.00	0.00
311.00	Melissa	hawa	0.00	0.00	1.00	0.00
312.00	Kato	zefa	1.00	0.00	0.00	0.00
313.00	Kato	zefa	1.00	0.00	0.00	0.00
314.00	Hawa	bwoba	1.00	0.00	0.00	0.00
315.00	Hawa	musa	1.00	1.00	0.00	0.00
316.00	Hawa	musa	1.00	1.00	0.00	0.00
317.00	Hawa	musa	1.00	1.00	0.00	0.00
318.00	Hawa	musa	1.00	1.00	0.00	0.00
319.00	Hawa	musa	1.00	1.00	0.00	0.00
320.00	Hawa	musa	1.00	1.00	0.00	0.00
321.00	Hawa	musa	1.00	1.00	0.00	0.00
322.00	Hawa	musa	1.00	1.00	0.00	0.00
323.00	Hawa	musa	1.00	1.00	0.00	0.00
324.00	Hawa	musa	1.00	1.00	0.00	0.00
325.00	Hawa	musa	1.00	1.00	0.00	0.00
326.00	Hawa	musa	1.00	1.00	0.00	0.00
327.00	Hawa	musa	1.00	1.00	0.00	0.00
328.00	Hawa	musa	1.00	1.00	0.00	0.00
329.00	Hawa	musa	1.00	1.00	0.00	0.00
330.00	Hawa	musa	1.00	1.00	0.00	0.00
331.00	Hawa	musa	1.00	1.00	0.00	0.00
332.00	Hawa	musa	1.00	1.00	0.00	0.00
333.00	Hawa	musa	1.00	1.00	0.00	0.00
334.00	Hawa	musa	1.00	1.00	0.00	0.00
335.00	Hawa	musa	1.00	1.00	0.00	0.00
336.00	Kato	bwoba	1.00	0.00	0.00	0.00
337.00	Kato	bwoba	1.00	0.00	0.00	0.00
338.00	Kato	bwoba	1.00	0.00	0.00	0.00

339.00	Kato	bwoba	1.00	0.00	0.00	0.00
340.00	Kato	bwoba	1.00	0.00	0.00	0.00
341.00	Kato	bwoba	1.00	0.00	0.00	0.00
342.00	Kato	bwoba	1.00	0.00	0.00	0.00
343.00	Kato	bwoba	1.00	0.00	0.00	0.00
344.00	Kato	bwoba	1.00	0.00	0.00	0.00
345.00	Kato	bwoba	1.00	0.00	0.00	0.00
346.00	Kato	bwoba	1.00	0.00	0.00	0.00
347.00	Kato	bwoba	1.00	0.00	0.00	0.00
348.00	Kato	bwoba	1.00	0.00	0.00	0.00
349.00	Kato	bwoba	1.00	0.00	0.00	0.00
350.00	Kato	bwoba	1.00	0.00	0.00	0.00
351.00	Kato	bwoba	1.00	0.00	0.00	0.00
352.00	Kato	bwoba	1.00	0.00	0.00	0.00
353.00	Melissa	nick	0.00	0.00	1.00	0.00
354.00	Melissa	nick	0.00	0.00	1.00	0.00
355.00	Melissa	nick	0.00	0.00	1.00	0.00
356.00	Melissa	nick	0.00	0.00	999.00	0.00
357.00	Squibs	hawa	1.00	1.00	0.00	0.00
358.00	Bwoba	hawa	1.00	0.00	0.00	0.00
359.00	Bwoba	hawa	1.00	0.00	0.00	0.00
360.00	Bwoba	maani	1.00	0.00	0.00	0.00
361.00	Ruhara	melissa	1.00	0.00	0.00	0.00
362.00	Ruhara	nick	0.00	0.00	999.00	1.00
363.00	Hawa	zefa	1.00	0.00	0.00	0.00
364.00	Ruhara	zefa	0.00	0.00	0.00	0.00
365.00	Kato	musa	1.00	1.00	0.00	0.00
366.00	Kato	musa	1.00	1.00	0.00	0.00
367.00	Kato	musa	1.00	1.00	0.00	0.00
368.00	Nambi	nick	0.00	0.00	0.00	0.00
369.00	Hawa	nick	1.00	0.00	0.00	0.00
370.00	Kato	hawa	1.00	1.00	0.00	0.00

371.00	Squibs	melissa	0.00	0.00	0.00	0.00
372.00	Squibs	melissa	0.00	0.00	0.00	0.00
373.00	Hawa	hariette	0.00	0.00	0.00	1.00
374.00	Hawa	anna	0.00	1.00	1.00	0.00
375.00	Ruhara	kewaya	1.00	0.00	0.00	0.00
376.00	Ruhara	nick	0.00	0.00	0.00	1.00
377.00	Ruhara	nick	0.00	0.00	0.00	1.00
378.00	Zimba	simon	0.00	0.00	0.00	0.00
379.00	Ruhara	nick	0.00	0.00	0.00	1.00
380.00	Ruhara	nick	0.00	0.00	0.00	1.00
381.00	Ruhara	nick	0.00	0.00	999.00	1.00
382.00	Ruhara	kwera	1.00	0.00	0.00	0.00
383.00	Kwera	zimba	1.00	0.00	0.00	0.00
384.00	Kato	anna	0.00	1.00	1.00	0.00
385.00	Kato	anna	0.00	1.00	1.00	0.00
386.00	Kato	anna	0.00	1.00	0.00	0.00
387.00	Kato	anna	0.00	1.00	0.00	0.00
388.00	Kato	anna	0.00	1.00	0.00	0.00
389.00	Kato	zimba	0.00	0.00	1.00	0.00
390.00	Kato	zimba	0.00	0.00	1.00	0.00
391.00	Hawa	musa	1.00	1.00	0.00	0.00
392.00	Hawa	zefa	1.00	0.00	0.00	0.00
393.00	Hawa	zefa	1.00	0.00	0.00	0.00
394.00	Hawa	zefa	1.00	0.00	0.00	0.00
395.00	Hawa	zefa	1.00	0.00	0.00	0.00
396.00	Hawa	zefa	1.00	0.00	0.00	0.00
397.00	Hawa	zefa	1.00	0.00	0.00	0.00
398.00	Hawa	zefa	1.00	0.00	0.00	0.00
399.00	Hawa	zefa	1.00	0.00	0.00	0.00
400.00	Hawa	zefa	1.00	0.00	0.00	0.00
401.00	Hawa	zefa	1.00	0.00	0.00	0.00
402.00	Hawa	zefa	1.00	0.00	0.00	0.00

403.00	Hawa	zefa	1.00	0.00	0.00	0.00
404.00	Hawa	zefa	1.00	0.00	0.00	0.00
405.00	Hawa	zefa	1.00	0.00	0.00	0.00
406.00	Hawa	zefa	1.00	0.00	0.00	0.00
407.00	Hawa	zefa	1.00	0.00	0.00	0.00
408.00	Hawa	zefa	1.00	0.00	0.00	0.00
409.00	Hawa	zefa	1.00	0.00	0.00	0.00
410.00	Hawa	zefa	1.00	0.00	0.00	0.00
411.00	Hawa	zefa	1.00	0.00	0.00	0.00
412.00	Hawa	zefa	1.00	0.00	0.00	0.00
413.00	Hawa	zefa	1.00	0.00	0.00	0.00
414.00	Hawa	zefa	1.00	0.00	0.00	0.00
415.00	Hawa	zefa	1.00	0.00	0.00	0.00
416.00	Hawa	zefa	1.00	0.00	0.00	0.00
417.00	Hawa	zefa	1.00	0.00	0.00	0.00
418.00	Hawa	zefa	1.00	0.00	0.00	0.00
419.00	Hawa	zefa	1.00	0.00	0.00	0.00
420.00	Hawa	zefa	1.00	0.00	0.00	0.00
421.00	Hawa	zefa	1.00	0.00	0.00	0.00
422.00	Hawa	zefa	1.00	0.00	0.00	0.00
423.00	Hawa	zefa	1.00	0.00	0.00	0.00
424.00	Hawa	zefa	1.00	0.00	0.00	0.00
425.00	Hawa	zefa	1.00	0.00	0.00	0.00
426.00	Hawa	zefa	1.00	0.00	0.00	0.00
427.00	Hawa	zefa	1.00	0.00	0.00	0.00
428.00	Hawa	zefa	1.00	0.00	0.00	0.00
429.00	Hawa	zefa	1.00	0.00	0.00	0.00
430.00	Hawa	zefa	1.00	0.00	0.00	0.00
431.00	Hawa	zefa	1.00	0.00	0.00	0.00
432.00	Hawa	zefa	1.00	0.00	0.00	0.00
433.00	Hawa	anna	0.00	1.00	1.00	0.00
434.00	Hawa	janie	0.00	0.00	999.00	0.00

435.00	Hawa	zimba	0.00	0.00	1.00	0.00
436.00	Hawa	zimba	0.00	0.00	1.00	0.00
437.00	Kato	sarine	0.00	0.00	0.00	0.00
438.00	Hawa	zimba	0.00	0.00	1.00	0.00
439.00	Hawa	zimba	0.00	0.00	1.00	0.00
440.00	Hawa	zefa	1.00	0.00	0.00	0.00
441.00	Nick	melissa	0.00	0.00	1.00	0.00
442.00	Hawa	melissa	0.00	0.00	1.00	0.00
443.00	Nick	bwoba	1.00	1.00	0.00	0.00
444.00	Nick	nambi	0.00	0.00	0.00	0.00
445.00	Nick	musa	1.00	0.00	0.00	0.00
446.00	Nick	bwoba	1.00	1.00	0.00	0.00
447.00	Nick	bwoba	1.00	1.00	0.00	0.00
448.00	Nick	bwoba	1.00	1.00	0.00	0.00
449.00	Nick	melissa	0.00	0.00	1.00	0.00
450.00	Nick	melissa	0.00	0.00	1.00	0.00
451.00	Nick	melissa	0.00	0.00	1.00	0.00
452.00	Nick	melissa	0.00	0.00	1.00	0.00
453.00	Nick	melissa	0.00	0.00	1.00	0.00
454.00	Nick	melissa	0.00	0.00	1.00	0.00
455.00	Nick	melissa	0.00	0.00	0.00	0.00
456.00	Nick	melissa	0.00	0.00	0.00	0.00
457.00	Bwoba	nambi	0.00	0.00	0.00	0.00
458.00	Bwoba	squibs	1.00	1.00	0.00	0.00
459.00	Bwoba	nick	1.00	1.00	0.00	0.00
460.00	Bwoba	nick	1.00	1.00	0.00	0.00
461.00	Nick	kato	1.00	0.00	0.00	0.00
462.00	Musa	maani	1.00	0.00	0.00	0.00
463.00	Bwoba	musa	1.00	1.00	0.00	0.00
464.00	Kato	anna	0.00	1.00	1.00	0.00
465.00	Kato	musa	1.00	1.00	0.00	0.00
466.00	Bwoba	nambi	0.00	0.00	1.00	0.00

467.00	Bwoba	nambi	0.00	0.00	1.00	0.00
468.00	Kato	zimba	0.00	0.00	1.00	0.00
469.00	Kato	zimba	0.00	0.00	1.00	0.00
470.00	Bwoba	kalema	0.00	0.00	1.00	0.00
471.00	Bwoba	kalema	0.00	0.00	1.00	0.00
472.00	Kato	kalema	0.00	0.00	999.00	0.00
473.00	Kato	kalema	0.00	0.00	999.00	0.00
474.00	Kato	wilma	0.00	0.00	1.00	0.00
475.00	Kato	wilma	0.00	0.00	1.00	0.00
476.00	Kato	wilma	0.00	0.00	1.00	0.00
477.00	Kato	wilma	0.00	0.00	1.00	0.00
478.00	Kato	wilma	0.00	0.00	1.00	0.00
479.00	Kato	wilma	0.00	0.00	1.00	0.00
480.00	Kato	wilma	0.00	0.00	1.00	0.00
481.00	Kato	wilma	0.00	0.00	1.00	0.00
482.00	Kato	wilma	0.00	0.00	1.00	0.00
483.00	Kato	wilma	0.00	0.00	1.00	0.00
484.00	Nick	melissa	0.00	0.00	1.00	0.00
485.00	Nick	melissa	0.00	0.00	1.00	0.00
486.00	Squibs	anna	0.00	1.00	1.00	0.00
487.00	Squibs	anna	0.00	1.00	1.00	
488.00	Hawa	melissa	0.00	0.00	1.00	0.00
489.00	Hawa	melissa	0.00	0.00	1.00	0.00
490.00	Hawa	melissa	0.00	0.00	1.00	0.00
491.00	Hawa	melissa	0.00	0.00	1.00	0.00
492.00	Hawa	melissa	0.00	0.00	1.00	0.00
493.00	Hawa	melissa	0.00	0.00	1.00	0.00
494.00	Hawa	nick	1.00	0.00	0.00	0.00
495.00	Hawa	zimba	0.00	0.00	1.00	0.00
496.00	Kato	zimba	0.00	0.00	1.00	0.00
497.00	Hawa	zimba	0.00	0.00	1.00	0.00
498.00	Hawa	zimba	0.00	0.00	1.00	0.00

499.00	Kato	zimba	0.00	0.00	1.00	0.00
500.00	Bwoba	nick	1.00	1.00	0.00	0.00
501.00	Nambi	nick	0.00	0.00	0.00	0.00
502.00	Kutu	nick	0.00	1.00	0.00	0.00
503.00	Zimba	hawa	0.00	0.00	0.00	0.00
504.00	Zimba	musa	0.00	0.00	999.00	0.00
505.00	Zimba	nick	0.00	0.00	0.00	0.00
506.00	Kato	nick	1.00	0.00	0.00	0.00
507.00	Squibs	bwoba	1.00	1.00	0.00	0.00
508.00	Zimba	nick	0.00	0.00	1.00	0.00
509.00	Squibs	nick	1.00	0.00	0.00	0.00
510.00	Musa	kato	1.00	1.00	0.00	0.00
511.00	Musa	kato	1.00	1.00	0.00	0.00
512.00	Nick	musa	1.00	0.00	0.00	0.00
513.00	Kato	nick	1.00	0.00	0.00	0.00
514.00	Zimba	kato	0.00	0.00	999.00	0.00
515.00	Kutu	Beatrice	1.00	1.00	0.00	0.00
516.00	Hawa	bwoba	1.00	0.00	0.00	0.00
517.00	Musa	nick	1.00	0.00	0.00	0.00
518.00	Melissa	simon	0.00	0.00	1.00	0.00
519.00	Melissa	nick	0.00	0.00	1.00	0.00
520.00	Melissa	nick	0.00	0.00	999.00	0.00
521.00	Melissa	nick	0.00	0.00	1.00	0.00
522.00	Kato	nick	1.00	0.00	0.00	0.00
523.00	Hawa	musa	1.00	1.00	0.00	0.00
524.00	Melissa	nick	0.00	0.00	1.00	0.00
525.00	Ruhara	nick	0.00	0.00	0.00	1.00
526.00	Kato	musa	1.00	1.00	0.00	0.00
527.00	Nambi	nick	0.00	0.00	0.00	0.00
528.00	Hawa	musa	1.00	1.00	0.00	0.00
529.00	Hawa	nick	1.00	0.00	0.00	0.00
530.00	Hawa	nick	1.00	0.00	0.00	0.00

531.00	Melissa	nick	0.00	0.00	0.00	0.00
532.00	Melissa	nick	0.00	0.00	0.00	0.00
533.00	Zimba	nick	0.00	0.00	0.00	0.00
534.00	Zimba	nick	0.00	0.00	0.00	0.00
535.00	Ruhara	nick	0.00	0.00	0.00	1.00
536.00	Kwera	nick	0.00	1.00	0.00	0.00
537.00	Kwera	nick	0.00	1.00	0.00	0.00
538.00	Kato	nambi	0.00	0.00	0.00	0.00
539.00	Kato	nick	1.00	0.00	0.00	0.00
540.00	Kato	nick	1.00	0.00	0.00	0.00
541.00	Kato	nick	1.00	0.00	0.00	0.00
542.00	Kutu	nick	0.00	1.00	0.00	0.00
543.00	Kutu	nick	0.00	1.00	0.00	0.00
544.00	Hawa	nick	1.00	0.00	0.00	0.00
545.00	Hawa	zefa	1.00	0.00	0.00	0.00
