# Supplementary material for: Convergence and divergence in gesture repertoires as an adaptive mechanism for social bonding in primates
Source: R Soc Open Sci. 2017 Nov 29;4(11):170181. doi: 10.1098/rsos.170181 (PMC5717623; doi:10.1098/rsos.170181)
Supplement: Supplementary Information 4 [file rsos170181supp4.pdf]

Convergence and divergence in gesture repertoires as an adaptive mechanism for social bonding in primates

Anna Ilona Roberts, Sam George Bradley Roberts

Royal Society Open Science

Supplementary Information 4

GLMM models dataset

matching number	Joint feeding	Joint resting	Joint travel	Grooming given	Grooming received	Grooming mutual	Attention present	Attention absent	Proximity
1.00	1.48	0.00	0.00	0.00	0.49	0.00	2.46	1.97	4.43
2.00	0.00	1.26	0.00	0.00	0.00	0.00	1.26	0.00	1.26
3.00	0.00	0.00	0.00	0.00	0.00	0.00	0.00	0.00	0.00
4.00	0.90	4.51	0.90	6.77	0.00	0.00	3.61	10.38	13.98
5.00	1.88	0.47	1.41	0.47	0.00	0.00	1.88	2.34	4.22
6.00	0.90	4.51	0.90	6.77	0.00	0.00	3.61	10.38	13.98
7.00	0.90	4.51	0.90	6.77	0.00	0.00	3.61	10.38	13.98
8.00	0.00	0.54	0.54	3.75	0.00	0.00	2.68	2.14	4.82
9.00	0.28	0.55	0.00	0.83	0.00	0.00	0.55	2.76	3.32
10.00	0.48	0.00	0.00	0.00	0.00	0.00	0.48	0.00	0.48
11.00	0.90	4.51	0.90	6.77	0.00	0.00	3.61	10.38	13.98
12.00	1.63	6.73	0.82	0.82	0.82	0.20	5.51	6.33	11.84
13.00	0.00	1.26	0.00	0.00	0.00	0.00	1.26	0.00	1.26
14.00	0.00	0.00	0.00	0.00	0.00	0.00	1.76	1.76	3.53
15.00	1.19	2.38	0.59	2.97	0.00	1.78	4.16	5.94	10.10
16.00	0.90	4.51	0.90	6.77	0.00	0.00	3.61	10.38	13.98
17.00	1.88	0.47	1.41	0.47	0.00	0.00	1.88	2.34	4.22
18.00	1.86	0.47	0.00	2.33	0.00	2.33	5.58	1.40	6.98

19.00	0.00	1.80	0.00	0.00	0.90	2.71	5.41	1.80	7.22
20.00	0.26	0.52	0.00	0.00	0.00	0.52	1.31	0.26	1.57
21.00	0.28	0.55	0.00	0.83	0.00	0.00	0.55	2.76	3.32
22.00	1.63	6.73	0.82	0.82	0.82	0.20	5.51	6.33	11.84
23.00	0.90	4.51	0.90	6.77	0.00	0.00	3.61	10.38	13.98
24.00	0.90	4.51	0.90	6.77	0.00	0.00	3.61	10.38	13.98
25.00	0.90	4.51	0.90	6.77	0.00	0.00	3.61	10.38	13.98
26.00	0.49	2.44	0.00	2.44	0.00	0.49	3.90	2.44	6.34
27.00	1.98	0.66	0.00	0.00	1.32	0.00	0.66	3.30	3.96
28.00	0.00	1.57	0.00	0.26	0.00	0.00	1.57	0.78	2.35
29.00	3.00	3.30	0.00	4.20	0.90	2.40	9.30	10.20	19.50
30.00	3.53	0.00	0.00	0.00	0.00	0.00	3.53	0.00	3.53
31.00	0.66	0.00	0.00	0.00	0.00	0.00	0.66	0.00	0.66
32.00	0.66	0.00	0.00	0.00	0.00	0.00	0.66	0.00	0.66
33.00	0.00	0.54	0.54	3.75	0.00	0.00	2.68	2.14	4.82
34.00	1.19	2.38	0.59	2.97	0.00	1.78	4.16	5.94	10.10
35.00	0.00	4.00	8.00	4.00	4.00	16.00	32.00	8.00	40.00
36.00	1.19	2.38	0.59	2.97	0.00	1.78	4.16	5.94	10.10
37.00	0.00	1.57	0.00	0.26	0.00	0.00	1.57	0.78	2.35
38.00	0.00	0.00	0.00	0.71	0.00	0.00	0.71	0.71	1.43
39.00	1.19	2.38	0.59	2.97	0.00	1.78	4.16	5.94	10.10
40.00	1.19	2.38	0.59	2.97	0.00	1.78	4.16	5.94	10.10
41.00	1.19	2.38	0.59	2.97	0.00	1.78	4.16	5.94	10.10
42.00	0.00	0.00	1.98	0.00	0.00	0.00	0.00	1.98	2.64
43.00	0.00	1.20	3.00	0.00	0.00	5.40	6.60	3.60	10.20
44.00	4.29	0.00	0.00	0.00	0.00	0.00	4.29	0.00	4.29
45.00	0.00	0.00	0.00	0.00	0.00	0.00	0.00	0.00	0.00
46.00	0.00	0.00	0.00	0.00	0.00	0.00	0.00	0.00	0.00
47.00	0.00	0.00	0.00	0.00	0.00	0.00	0.00	0.00	0.00
48.00	0.00	0.00	0.00	0.00	0.00	0.00	0.00	0.00	0.00
49.00	0.00	4.00	8.00	4.00	4.00	16.00	32.00	8.00	40.00
50.00	4.29	0.00	0.00	0.00	0.00	0.00	4.29	0.00	4.29

51.00	0.00	0.00	1.98	0.00	0.00	0.00	0.66	1.98	2.64
52.00	5.19	2.96	0.74	0.74	2.96	0.00	5.93	8.89	14.81
53.00	2.73	2.73	2.73	0.00	0.00	0.00	2.73	8.18	10.91
54.00	0.00	0.00	1.98	0.00	0.00	0.00	0.66	1.98	2.64
55.00	0.00	1.20	3.00	0.00	0.00	5.40	6.60	3.60	10.20
56.00	0.66	0.00	0.00	0.00	0.00	0.00	0.66	0.00	0.66
57.00	0.67	0.34	0.00	0.00	0.00	0.00	1.69	0.67	2.36
58.00	0.90	4.51	0.90	6.77	0.00	0.00	3.61	10.38	13.98
59.00	0.00	0.00	0.00	0.00	0.00	0.00	0.00	0.00	0.00
60.00	0.00	1.26	0.00	0.00	0.00	0.00	1.26	0.00	1.26
61.00	0.00	0.54	0.54	3.75	0.00	0.00	2.68	2.14	4.82
62.00	0.00	0.51	0.00	0.00	0.00	0.00	0.51	1.02	1.53
63.00	0.49	2.44	0.00	2.44	0.00	0.49	3.90	2.44	6.34
64.00	0.00	1.26	0.00	0.00	0.00	0.00	1.26	0.00	1.26
65.00	0.00	1.18	0.00	0.00	0.00	0.00	8.24	0.00	2.35
66.00	0.00	1.57	0.00	0.26	0.00	0.00	1.57	0.78	2.35
67.00	0.00	0.00	0.00	0.00	0.00	0.00	0.00	0.00	0.00
68.00	0.39	1.57	1.18	0.00	15.29	0.00	14.12	4.71	18.82
69.00	0.00	1.57	0.00	0.26	0.00	0.00	1.57	0.78	2.35
70.00	0.00	0.00	0.00	0.00	0.00	0.00	0.00	0.00	0.00
71.00	0.00	1.26	0.00	0.00	0.00	0.00	1.26	0.00	1.26
72.00	0.00	1.26	0.00	0.00	0.00	0.00	1.26	0.00	1.26
73.00	0.49	2.44	0.00	2.44	0.00	0.49	3.90	2.44	6.34
74.00	0.49	2.44	0.00	2.44	0.00	0.49	3.90	2.44	6.34
75.00	0.00	1.20	3.00	0.00	0.00	5.40	6.60	3.60	10.20
76.00	1.86	0.47	0.00	2.33	0.00	2.33	5.58	1.40	6.98
77.00	0.00	1.57	0.00	0.26	0.00	0.00	1.57	0.78	2.35
78.00	0.00	0.00	0.00	0.00	0.00	0.00	1.68	1.12	2.80
79.00	0.00	0.00	1.30	2.61	0.00	2.61	0.00	1.30	1.30
80.00	0.77	0.00	0.77	0.00	0.00	0.00	0.77	1.54	2.31
81.00	0.00	1.57	0.00	0.26	0.00	0.00	1.57	0.78	2.35
82.00	0.00	1.57	0.00	0.26	0.00	0.00	1.57	0.78	2.35

83.00	0.00	0.00	0.00	0.00	0.00	0.00	0.00	0.00	0.00
84.00	0.00	1.18	0.00	0.00	0.00	0.00	8.24	0.00	2.35
85.00	0.28	0.55	0.00	0.83	0.00	0.00	0.55	2.76	3.32
86.00	0.00	0.00	0.00	0.00	0.00	0.00	0.00	0.00	0.00
87.00	0.00	0.00	0.00	0.00	0.00	0.00	0.00	0.00	0.00
88.00	0.00	0.00	0.00	0.00	0.00	0.00	0.00	0.00	0.00
89.00	0.00	0.00	0.00	0.71	0.00	0.00	0.71	0.71	1.43
90.00	1.63	6.73	0.82	0.82	0.82	0.20	5.51	6.33	11.84
91.00	0.49	2.44	0.00	2.44	0.00	0.49	3.90	2.44	6.34
92.00	0.90	4.51	0.90	6.77	0.00	0.00	3.61	10.38	13.98
93.00	1.63	6.73	0.82	0.82	0.82	0.20	5.51	6.33	11.84
94.00	0.00	1.26	0.00	0.00	0.00	0.00	1.26	0.00	1.26
95.00	0.00	0.00	0.00	0.00	0.00	0.00	0.00	0.00	0.00
96.00	0.90	4.51	0.90	6.77	0.00	0.00	3.61	10.38	13.98
97.00	0.00	1.57	0.00	0.26	0.00	0.00	1.57	0.78	2.35
98.00	0.00	0.00	0.00	0.00	0.00	0.00	0.00	0.00	0.00
99.00	0.00	1.57	0.00	0.26	0.00	0.00	1.57	0.78	2.35
100.00	0.00	0.00	0.00	0.00	0.00	0.00	1.76	1.76	3.53
101.00	0.00	1.57	0.00	0.26	0.00	0.00	1.57	0.78	2.35
102.00	0.00	0.54	0.54	3.75	0.00	0.00	2.68	2.14	4.82
103.00	0.00	0.54	0.54	3.75	0.00	0.00	2.68	2.14	4.82
104.00	0.00	0.54	0.54	3.75	0.00	0.00	2.68	2.14	4.82
105.00	2.24	2.69	1.34	2.24	1.34	0.45	4.48	6.27	10.75
106.00	0.00	0.00	0.00	0.00	0.00	0.00	0.00	0.00	0.00
107.00	0.48	0.00	0.00	0.00	0.00	0.00	0.48	0.00	0.48
108.00	0.00	4.00	8.00	4.00	4.00	16.00	32.00	8.00	40.00
109.00	0.00	25.71	0.00	0.00	0.00	0.00	17.14	8.57	25.71
110.00	2.24	2.69	1.34	2.24	1.34	0.45	4.48	6.27	10.75
111.00	2.24	2.69	1.34	2.24	1.34	0.45	4.48	6.27	10.75
112.00	2.24	2.69	1.34	2.24	1.34	0.45	4.48	6.27	10.75
113.00	2.24	2.69	1.34	2.24	1.34	0.45	4.48	6.27	10.75
114.00	0.00	4.00	8.00	4.00	4.00	16.00	32.00	8.00	40.00

115.00	0.00	4.00	8.00	4.00	4.00	16.00	32.00	8.00	40.00
116.00	0.00	0.61	0.00	0.61	0.00	0.00	0.61	0.61	1.22
117.00	0.00	0.00	0.00	0.00	0.00	0.00	0.00	2.86	2.86
118.00	2.07	0.00	3.10	0.00	7.24	1.03	5.17	5.17	10.34
119.00	0.00	0.00	0.00	0.00	0.00	0.00	0.00	0.00	0.00
120.00	0.00	0.61	0.00	0.61	0.00	0.00	0.61	0.61	1.22
121.00	0.00	0.61	0.00	0.61	0.00	0.00	0.61	0.61	1.22
122.00	0.00	4.00	8.00	4.00	4.00	16.00	32.00	8.00	40.00
123.00	0.00	0.00	0.00	0.00	0.00	0.00	0.00	0.00	0.00
124.00	2.24	2.69	1.34	2.24	1.34	0.45	4.48	6.27	10.75
125.00	2.24	2.69	1.34	2.24	1.34	0.45	4.48	6.27	10.75
126.00	2.24	2.69	1.34	2.24	1.34	0.45	4.48	6.27	10.75
127.00	2.24	2.69	1.34	2.24	1.34	0.45	4.48	6.27	10.75
128.00	0.00	4.00	8.00	4.00	4.00	16.00	32.00	8.00	40.00
129.00	0.00	4.00	8.00	4.00	4.00	16.00	32.00	8.00	40.00
130.00	2.07	0.00	3.10	0.00	7.24	1.03	5.17	5.17	0.00
131.00	0.00	4.00	8.00	4.00	4.00	16.00	32.00	8.00	40.00
132.00	2.24	2.69	1.34	2.24	1.34	0.45	4.48	6.27	10.75
133.00	0.00	0.00	0.00	0.00	0.00	0.00	0.00	0.00	0.00
134.00	0.00	0.00	0.00	0.00	0.00	0.00	0.00	0.00	0.00
135.00	0.00	0.00	1.98	0.00	0.00	0.00	0.66	1.98	2.64
136.00	0.00	6.00	0.00	6.00	6.00	6.00	24.00	0.00	24.00
137.00	0.48	0.00	0.00	0.00	0.00	0.00	0.48	0.00	0.48
138.00	0.00	6.00	0.00	6.00	6.00	6.00	24.00	0.00	24.00
139.00	5.00	0.00	1.25	0.00	0.00	0.00	0.00	6.25	6.25
140.00	5.00	0.00	1.25	0.00	0.00	0.00	0.00	6.25	6.25
141.00	0.48	0.00	0.00	0.00	0.00	0.00	0.48	0.00	0.48
142.00	0.00	6.00	0.00	6.00	6.00	6.00	24.00	0.00	24.00
143.00	0.00	6.00	0.00	6.00	6.00	6.00	24.00	0.00	24.00
144.00	0.00	6.00	0.00	6.00	6.00	6.00	24.00	0.00	24.00
145.00	0.66	0.00	0.00	0.00	0.00	0.00	0.66	0.00	0.66
146.00	0.48	0.00	0.00	0.00	0.00	0.00	0.48	0.00	0.48

147.00	1.88	0.47	1.41	0.47	0.00	0.00	1.88	2.34	4.22
148.00	1.88	0.47	1.41	0.47	0.00	0.00	1.88	2.34	4.22
149.00	1.88	0.47	1.41	0.47	0.00	0.00	1.88	2.34	4.22
150.00	2.24	2.69	1.34	2.24	1.34	0.45	4.48	6.27	10.75
151.00	0.00	1.57	0.00	0.26	0.00	0.00	1.57	0.78	2.35
152.00	1.98	0.66	0.00	0.00	1.32	0.00	0.66	3.30	3.96
153.00	1.98	0.66	0.00	0.00	1.32	0.00	0.66	3.30	3.96
154.00	1.63	6.73	0.82	0.82	0.82	0.20	5.51	6.33	11.84
155.00	0.00	0.51	0.00	0.00	0.00	0.00	0.51	1.02	1.53
156.00	1.63	6.73	0.82	0.82	0.82	0.20	5.51	6.33	11.84
157.00	0.00	1.57	0.00	0.26	0.00	0.00	1.57	0.78	2.35
158.00	1.48	0.00	0.00	0.00	0.49	0.00	2.46	1.97	4.43
159.00	0.00	1.71	0.00	0.00	0.00	0.00	1.71	0.00	1.71
160.00	1.63	6.73	0.82	0.82	0.82	0.20	5.51	6.33	11.84
161.00	0.48	0.00	0.00	0.00	0.00	0.00	0.48	0.00	0.48
162.00	0.48	0.00	0.00	0.00	0.00	0.00	0.48	0.00	0.48
163.00	0.48	0.00	0.00	0.00	0.00	0.00	0.48	0.00	0.48
164.00	1.88	0.47	1.41	0.47	0.00	0.00	1.88	2.34	4.22
165.00	0.00	1.67	1.67	0.00	11.67	0.00	3.33	11.67	15.00
166.00	0.66	0.00	0.00	0.00	0.00	0.00	0.66	0.00	0.66
167.00	0.66	0.00	0.00	0.00	0.00	0.00	0.66	0.00	0.66
168.00	0.00	0.00	0.00	0.00	0.00	0.00	0.00	0.00	0.00
169.00	0.00	0.00	0.00	0.00	0.00	0.00	0.00	0.00	3.16
170.00	0.00	0.00	0.00	11.43	2.86	2.86	14.29	0.00	14.29
171.00	0.00	0.00	0.00	11.43	2.86	2.86	14.29	0.00	14.29
172.00	0.00	0.00	0.00	11.43	2.86	2.86	14.29	0.00	14.29
173.00	0.00	0.00	0.00	11.43	2.86	2.86	14.29	0.00	14.29
174.00	0.00	0.00	0.00	11.43	2.86	2.86	14.29	0.00	14.29
175.00	4.29	0.00	0.00	0.00	0.00	0.00	4.29	0.00	4.29
176.00	4.29	0.00	0.00	0.00	0.00	0.00	4.29	0.00	4.29
177.00	4.66	4.08	0.00	2.33	1.75	4.08	14.56	5.83	20.39
178.00	4.66	4.08	0.00	2.33	1.75	4.08	14.56	5.83	20.39

179.00	1.19	2.38	0.59	2.97	0.00	1.78	4.16	5.94	10.10
180.00	1.19	2.38	0.59	2.97	0.00	1.78	4.16	5.94	10.10
181.00	1.19	2.38	0.59	2.97	0.00	1.78	4.16	5.94	10.10
182.00	1.19	2.38	0.59	2.97	0.00	1.78	4.16	5.94	10.10
183.00	1.19	2.38	0.59	2.97	0.00	1.78	4.16	5.94	10.10
184.00	5.19	2.96	0.74	0.74	2.96	0.00	5.93	8.89	14.81
185.00	5.19	2.96	0.74	0.74	2.96	0.00	5.93	8.89	14.81
186.00	0.00	0.00	1.30	2.61	0.00	2.61	0.00	1.30	1.30
187.00	1.48	0.00	0.00	0.00	0.49	0.00	2.46	1.97	4.43
188.00	1.48	0.00	0.00	0.00	0.49	0.00	2.46	1.97	4.43
189.00	5.77	0.58	1.73	0.00	6.35	0.00	6.35	8.08	14.42
190.00	5.77	0.58	1.73	0.00	6.35	0.00	6.35	8.08	14.42
191.00	5.77	0.58	1.73	0.00	6.35	0.00	6.35	8.08	14.42
192.00	5.77	0.58	1.73	0.00	6.35	0.00	6.35	8.08	14.42
193.00	0.00	0.00	0.00	0.00	0.00	0.00	0.00	0.00	0.00
194.00	0.00	0.00	0.00	0.00	0.00	0.00	0.00	0.00	0.00
195.00	2.24	2.69	1.34	2.24	1.34	0.45	4.48	6.27	10.75
196.00	0.00	4.00	8.00	4.00	4.00	16.00	32.00	8.00	40.00
197.00	0.00	4.00	8.00	4.00	4.00	16.00	32.00	8.00	40.00
198.00	0.00	4.00	8.00	4.00	4.00	16.00	32.00	8.00	40.00
199.00	0.00	4.00	8.00	4.00	4.00	16.00	32.00	8.00	40.00
200.00	0.00	4.00	8.00	4.00	4.00	16.00	32.00	8.00	40.00
201.00	0.00	4.00	8.00	4.00	4.00	16.00	32.00	8.00	40.00
202.00	0.00	4.00	8.00	4.00	4.00	16.00	32.00	8.00	40.00
203.00	7.38	0.92	1.85	6.46	1.85	0.92	12.92	8.31	21.23
204.00	7.38	0.92	1.85	6.46	1.85	0.92	12.92	8.31	21.23
205.00	7.38	0.92	1.85	6.46	1.85	0.92	12.92	8.31	21.23
206.00	7.38	0.92	1.85	6.46	1.85	0.92	12.92	8.31	21.23
207.00	7.38	0.92	1.85	6.46	1.85	0.92	12.92	8.31	21.23
208.00	7.38	0.92	1.85	6.46	1.85	0.92	12.92	8.31	21.23
209.00	7.38	0.92	1.85	6.46	1.85	0.92	12.92	8.31	21.23
210.00	7.38	0.92	1.85	6.46	1.85	0.92	12.92	8.31	21.23

211.00	7.38	0.92	1.85	6.46	1.85	0.92	12.92	8.31	21.23
212.00	7.38	0.92	1.85	6.46	1.85	0.92	12.92	8.31	21.23
213.00	7.38	0.92	1.85	6.46	1.85	0.92	12.92	8.31	21.23
214.00	7.38	0.92	1.85	6.46	1.85	0.92	12.92	8.31	21.23
215.00	4.66	4.08	0.00	2.33	1.75	4.08	14.56	5.83	20.39
216.00	4.66	4.08	0.00	2.33	1.75	4.08	14.56	5.83	20.39
217.00	4.66	4.08	0.00	2.33	1.75	4.08	14.56	5.83	20.39
218.00	4.66	4.08	0.00	2.33	1.75	4.08	14.56	5.83	20.39
219.00	4.66	4.08	0.00	2.33	1.75	4.08	14.56	5.83	20.39
220.00	4.66	4.08	0.00	2.33	1.75	4.08	14.56	5.83	20.39
221.00	4.66	4.08	0.00	2.33	1.75	4.08	14.56	5.83	20.39
222.00	4.66	4.08	0.00	2.33	1.75	4.08	14.56	5.83	20.39
223.00	4.66	4.08	0.00	2.33	1.75	4.08	14.56	5.83	20.39
224.00	4.66	4.08	0.00	2.33	1.75	4.08	14.56	5.83	20.39
225.00	4.66	4.08	0.00	2.33	1.75	4.08	14.56	5.83	20.39
226.00	4.66	4.08	0.00	2.33	1.75	4.08	14.56	5.83	20.39
227.00	4.66	4.08	0.00	2.33	1.75	4.08	14.56	5.83	20.39
228.00	4.66	4.08	0.00	2.33	1.75	4.08	14.56	5.83	20.39
229.00	4.66	4.08	0.00	2.33	1.75	4.08	14.56	5.83	20.39
230.00	4.66	4.08	0.00	2.33	1.75	4.08	14.56	5.83	20.39
231.00	4.66	4.08	0.00	2.33	1.75	4.08	14.56	5.83	20.39
232.00	4.66	4.08	0.00	2.33	1.75	4.08	14.56	5.83	20.39
233.00	4.66	4.08	0.00	2.33	1.75	4.08	14.56	5.83	20.39
234.00	4.66	4.08	0.00	2.33	1.75	4.08	14.56	5.83	20.39
235.00	4.66	4.08	0.00	2.33	1.75	4.08	14.56	5.83	20.39
236.00	4.66	4.08	0.00	2.33	1.75	4.08	14.56	5.83	20.39
237.00	4.66	4.08	0.00	2.33	1.75	4.08	14.56	5.83	20.39
238.00	4.66	4.08	0.00	2.33	1.75	4.08	14.56	5.83	20.39
239.00	4.66	4.08	0.00	2.33	1.75	4.08	14.56	5.83	20.39
240.00	4.66	4.08	0.00	2.33	1.75	4.08	14.56	5.83	20.39
241.00	4.66	4.08	0.00	2.33	1.75	4.08	14.56	5.83	20.39
242.00	4.66	4.08	0.00	2.33	1.75	4.08	14.56	5.83	20.39

243.00	7.38	0.92	1.85	6.46	1.85	0.92	12.92	8.31	21.23
244.00	1.86	0.47	0.00	2.33	0.00	2.33	5.58	1.40	6.98
245.00	2.24	2.69	1.34	2.24	1.34	0.45	4.48	6.27	10.75
246.00	2.07	0.00	3.10	0.00	7.24	1.03	5.17	5.17	10.34
247.00	2.07	0.00	3.10	0.00	7.24	1.03	5.17	5.17	10.34
248.00	2.07	0.00	3.10	0.00	7.24	1.03	5.17	5.17	10.34
249.00	2.07	0.00	3.10	0.00	7.24	1.03	5.17	5.17	10.34
250.00	1.86	0.47	0.00	2.33	0.00	2.33	5.58	1.40	6.98
251.00	0.28	0.55	0.00	0.83	0.00	0.00	0.55	2.76	3.32
252.00	5.77	0.58	1.73	0.00	6.35	0.00	6.35	8.08	14.42
253.00	5.77	0.58	1.73	0.00	6.35	0.00	6.35	8.08	14.42
254.00	5.77	0.58	1.73	0.00	6.35	0.00	6.35	8.08	14.42
255.00	5.77	0.58	1.73	0.00	6.35	0.00	6.35	8.08	14.42
256.00	0.00	1.18	0.00	0.00	0.00	0.00	8.24	0.00	2.35
257.00	0.00	0.00	0.94	0.00	0.00	0.00	0.00	0.94	0.94
258.00	0.00	0.00	0.00	12.86	4.29	17.14	34.29	4.29	38.57
259.00	0.00	0.00	0.00	12.86	4.29	17.14	34.29	4.29	38.57
260.00	0.00	0.00	0.00	12.86	4.29	17.14	34.29	4.29	38.57
261.00	0.00	0.00	0.00	12.86	4.29	17.14	34.29	4.29	38.57
262.00	0.00	0.00	0.00	12.86	4.29	17.14	34.29	4.29	38.57
263.00	0.00	0.00	0.00	12.86	4.29	17.14	34.29	4.29	38.57
264.00	2.24	2.69	1.34	2.24	1.34	0.45	4.48	6.27	10.75
265.00	1.98	0.66	0.00	0.00	1.32	0.00	0.66	3.30	3.96
266.00	3.00	3.30	0.00	4.20	0.90	2.40	9.30	10.20	19.50
267.00	0.90	4.51	0.90	6.77	0.00	0.00	3.61	10.38	13.98
268.00	0.90	4.51	0.90	6.77	0.00	0.00	3.61	10.38	13.98
269.00	0.90	4.51	0.90	6.77	0.00	0.00	3.61	10.38	13.98
270.00	0.90	4.51	0.90	6.77	0.00	0.00	3.61	10.38	13.98
271.00	0.90	4.51	0.90	6.77	0.00	0.00	3.61	10.38	13.98
272.00	0.90	4.51	0.90	6.77	0.00	0.00	3.61	10.38	13.98
273.00	0.90	4.51	0.90	6.77	0.00	0.00	3.61	10.38	13.98
274.00	0.90	4.51	0.90	6.77	0.00	0.00	3.61	10.38	13.98

275.00	0.90	4.51	0.90	6.77	0.00	0.00	3.61	10.38	13.98
276.00	0.90	4.51	0.90	6.77	0.00	0.00	3.61	10.38	13.98
277.00	0.90	4.51	0.90	6.77	0.00	0.00	3.61	10.38	13.98
278.00	1.86	0.47	0.00	2.33	0.00	2.33	5.58	1.40	6.98
279.00	1.86	0.47	0.00	2.33	0.00	2.33	5.58	1.40	6.98
280.00	2.07	0.00	3.10	0.00	7.24	1.03	5.17	5.17	10.34
281.00	2.43	0.81	0.00	0.00	0.41	0.00	2.43	2.03	4.46
282.00	8.57	0.00	0.00	0.00	2.45	8.57	14.69	4.90	19.59
283.00	8.57	0.00	0.00	0.00	2.45	8.57	14.69	4.90	19.59
284.00	0.00	1.58	0.00	0.79	0.00	0.79	3.16	0.00	3.16
285.00	0.00	1.58	0.00	0.79	0.00	0.79	3.16	0.00	3.16
286.00	0.00	1.58	0.00	0.79	0.00	0.79	3.16	0.00	3.16
287.00	3.00	3.30	0.00	4.20	0.90	2.40	9.30	10.20	19.50
288.00	0.00	5.00	0.00	0.00	1.67	0.00	10.00	3.33	13.33
289.00	0.00	5.00	0.00	0.00	1.67	0.00	10.00	3.33	13.33
290.00	0.00	5.00	0.00	0.00	1.67	0.00	10.00	3.33	13.33
291.00	0.00	5.00	0.00	0.00	1.67	0.00	10.00	3.33	13.33
292.00	0.00	0.51	0.00	0.00	0.00	0.00	0.51	1.02	1.53
293.00	2.36	0.71	0.00	0.24	0.47	0.00	1.65	2.36	4.02
294.00	0.00	0.51	0.00	0.00	0.00	0.00	0.51	1.02	1.53
295.00	1.63	6.73	0.82	0.82	0.82	0.20	5.51	6.33	11.84
296.00	0.00	1.80	0.00	0.00	0.90	2.71	5.41	1.80	7.22
297.00	0.26	0.52	0.00	0.00	0.00	0.52	1.31	0.26	1.57
298.00	0.26	0.52	0.00	0.00	0.00	0.52	1.31	0.26	1.57
299.00	0.00	1.20	3.00	0.00	0.00	5.40	6.60	3.60	10.20
300.00	1.22	0.00	0.00	0.00	0.00	0.00	1.22	1.22	2.44
301.00	2.36	0.71	0.00	0.24	0.47	0.00	1.65	2.36	4.02
302.00	0.00	1.80	0.00	0.00	0.90	2.71	5.41	1.80	7.22
303.00	0.00	1.80	0.00	0.00	0.90	2.71	5.41	1.80	7.22
304.00	0.48	0.00	0.00	0.00	0.00	0.00	0.48	0.00	0.48
305.00	2.36	0.71	0.00	0.24	0.47	0.00	1.65	2.36	4.02
306.00	1.63	6.73	0.82	0.82	0.82	0.20	5.51	6.33	11.84

307.00	1.63	6.73	0.82	0.82	0.82	0.20	5.51	6.33	11.84
308.00	0.58	0.00	0.00	1.17	0.00	5.24	5.24	2.33	7.57
309.00	0.58	0.00	0.00	1.17	0.00	5.24	5.24	2.33	7.57
310.00	0.58	0.00	0.00	1.17	0.00	5.24	5.24	2.33	7.57
311.00	2.36	0.71	0.00	0.24	0.47	0.00	1.65	2.36	4.02
312.00	0.00	0.00	0.00	0.71	0.00	0.00	0.71	0.71	1.43
313.00	0.00	0.00	0.00	0.71	0.00	0.00	0.71	0.71	1.43
314.00	0.49	2.44	0.00	2.44	0.00	0.49	3.90	2.44	6.34
315.00	0.90	4.51	0.90	6.77	0.00	0.00	3.61	10.38	13.98
316.00	0.90	4.51	0.90	6.77	0.00	0.00	3.61	10.38	13.98
317.00	0.90	4.51	0.90	6.77	0.00	0.00	3.61	10.38	13.98
318.00	0.90	4.51	0.90	6.77	0.00	0.00	3.61	10.38	13.98
319.00	0.90	4.51	0.90	6.77	0.00	0.00	3.61	10.38	13.98
320.00	0.90	4.51	0.90	6.77	0.00	0.00	3.61	10.38	13.98
321.00	0.90	4.51	0.90	6.77	0.00	0.00	3.61	10.38	13.98
322.00	0.90	4.51	0.90	6.77	0.00	0.00	3.61	10.38	13.98
323.00	0.90	4.51	0.90	6.77	0.00	0.00	3.61	10.38	13.98
324.00	0.90	4.51	0.90	6.77	0.00	0.00	3.61	10.38	13.98
325.00	0.90	4.51	0.90	6.77	0.00	0.00	3.61	10.38	13.98
326.00	0.90	4.51	0.90	6.77	0.00	0.00	3.61	10.38	13.98
327.00	0.90	4.51	0.90	6.77	0.00	0.00	3.61	10.38	13.98
328.00	0.90	4.51	0.90	6.77	0.00	0.00	3.61	10.38	13.98
329.00	0.90	4.51	0.90	6.77	0.00	0.00	3.61	10.38	13.98
330.00	0.90	4.51	0.90	6.77	0.00	0.00	3.61	10.38	13.98
331.00	0.90	4.51	0.90	6.77	0.00	0.00	3.61	10.38	13.98
332.00	0.90	4.51	0.90	6.77	0.00	0.00	3.61	10.38	13.98
333.00	0.90	4.51	0.90	6.77	0.00	0.00	3.61	10.38	13.98
334.00	0.90	4.51	0.90	6.77	0.00	0.00	3.61	10.38	13.98
335.00	0.90	4.51	0.90	6.77	0.00	0.00	3.61	10.38	13.98
336.00	0.00	0.00	0.00	0.00	0.00	0.00	1.76	1.76	3.53
337.00	0.00	0.00	0.00	0.00	0.00	0.00	1.76	1.76	3.53
338.00	0.00	0.00	0.00	0.00	0.00	0.00	1.76	1.76	3.53

339.00	0.00	0.00	0.00	0.00	0.00	0.00	1.76	1.76	3.53
340.00	0.00	0.00	0.00	0.00	0.00	0.00	1.76	1.76	3.53
341.00	0.00	0.00	0.00	0.00	0.00	0.00	1.76	1.76	3.53
342.00	0.00	0.00	0.00	0.00	0.00	0.00	1.76	1.76	3.53
343.00	0.00	0.00	0.00	0.00	0.00	0.00	1.76	1.76	3.53
344.00	0.00	0.00	0.00	0.00	0.00	0.00	1.76	1.76	3.53
345.00	0.00	0.00	0.00	0.00	0.00	0.00	1.76	1.76	3.53
346.00	0.00	0.00	0.00	0.00	0.00	0.00	1.76	1.76	3.53
347.00	0.00	0.00	0.00	0.00	0.00	0.00	1.76	1.76	3.53
348.00	0.00	0.00	0.00	0.00	0.00	0.00	1.76	1.76	3.53
349.00	0.00	0.00	0.00	0.00	0.00	0.00	1.76	1.76	3.53
350.00	0.00	0.00	0.00	0.00	0.00	0.00	1.76	1.76	3.53
351.00	0.00	0.00	0.00	0.00	0.00	0.00	1.76	1.76	3.53
352.00	0.00	0.00	0.00	0.00	0.00	0.00	1.76	1.76	3.53
353.00	1.63	6.73	0.82	0.82	0.82	0.20	5.51	6.33	11.84
354.00	1.63	6.73	0.82	0.82	0.82	0.20	5.51	6.33	11.84
355.00	1.63	6.73	0.82	0.82	0.82	0.20	5.51	6.33	11.84
356.00	1.63	6.73	0.82	0.82	0.82	0.20	5.51	6.33	11.84
357.00	0.52	4.66	0.00	0.00	0.00	0.00	1.03	4.66	5.69
358.00	0.00	1.67	1.67	0.00	11.67	0.00	3.33	11.67	15.00
359.00	0.00	1.67	1.67	0.00	11.67	0.00	3.33	11.67	15.00
360.00	0.00	0.00	0.00	11.43	0.00	5.71	2.86	5.71	8.57
361.00	3.85	3.08	0.00	0.00	0.77	0.00	3.08	5.38	13.08
362.00	3.00	3.30	0.00	4.20	0.90	2.40	9.30	10.20	19.50
363.00	0.00	0.54	0.54	3.75	0.00	0.00	2.68	2.14	4.82
364.00	0.00	0.00	0.00	0.00	0.00	0.00	0.00	0.00	0.00
365.00	0.28	0.55	0.00	0.83	0.00	0.00	0.55	2.76	3.32
366.00	0.28	0.55	0.00	0.83	0.00	0.00	0.55	2.76	3.32
367.00	0.28	0.55	0.00	0.83	0.00	0.00	0.55	2.76	3.32
368.00	0.00	1.18	0.00	0.00	0.00	0.00	8.24	0.00	2.35
369.00	0.00	1.26	0.00	0.00	0.00	0.00	1.26	0.00	1.26
370.00	1.57	1.30	0.26	0.00	0.00	0.00	1.30	4.43	5.74

371.00	2.20	0.37	0.00	0.73	0.00	2.93	5.12	1.10	6.22
372.00	2.20	0.37	0.00	0.73	0.00	2.93	5.12	1.10	6.22
373.00	8.57	0.00	0.00	0.00	2.45	8.57	14.69	4.90	19.59
374.00	0.00	2.93	0.00	0.00	0.00	0.00	1.46	1.46	2.93
375.00	0.00	0.00	0.00	0.00	0.00	0.00	0.00	0.00	0.00
376.00	3.00	3.30	0.00	4.20	0.90	2.40	9.30	10.20	19.50
377.00	3.00	3.30	0.00	4.20	0.90	2.40	9.30	10.20	19.50
378.00	0.00	0.00	0.00	0.00	0.00	0.00	0.00	0.00	0.00
379.00	3.00	3.30	0.00	4.20	0.90	2.40	9.30	10.20	19.50
380.00	3.00	3.30	0.00	4.20	0.90	2.40	9.30	10.20	19.50
381.00	3.00	3.30	0.00	4.20	0.90	2.40	9.30	10.20	19.50
382.00	0.00	0.00	0.00	0.00	0.00	0.00	0.00	0.00	0.00
383.00	0.00	0.00	0.00	0.00	0.00	0.00	0.00	0.00	0.00
384.00	0.00	0.00	0.00	0.00	0.00	0.00	0.00	0.00	0.00
385.00	0.00	0.00	0.00	0.00	0.00	0.00	0.00	0.00	0.00
386.00	0.00	0.00	0.00	0.00	0.00	0.00	0.00	0.00	0.00
387.00	0.00	0.00	0.00	0.00	0.00	0.00	0.00	0.00	0.00
388.00	0.00	0.00	0.00	0.00	0.00	0.00	0.00	0.00	0.00
389.00	0.66	0.00	0.00	0.00	0.00	0.00	0.66	0.00	0.66
390.00	0.66	0.00	0.00	0.00	0.00	0.00	0.66	0.00	0.66
391.00	0.90	4.51	0.90	6.77	0.00	0.00	3.61	10.38	13.98
392.00	0.00	0.54	0.54	3.75	0.00	0.00	2.68	2.14	4.82
393.00	0.00	0.54	0.54	3.75	0.00	0.00	2.68	2.14	4.82
394.00	0.00	0.54	0.54	3.75	0.00	0.00	2.68	2.14	4.82
395.00	0.00	0.54	0.54	3.75	0.00	0.00	2.68	2.14	4.82
396.00	0.00	0.54	0.54	3.75	0.00	0.00	2.68	2.14	4.82
397.00	0.00	0.54	0.54	3.75	0.00	0.00	2.68	2.14	4.82
398.00	0.00	0.54	0.54	3.75	0.00	0.00	2.68	2.14	4.82
399.00	0.00	0.54	0.54	3.75	0.00	0.00	2.68	2.14	4.82
400.00	0.00	0.54	0.54	3.75	0.00	0.00	2.68	2.14	4.82
401.00	0.00	0.54	0.54	3.75	0.00	0.00	2.68	2.14	4.82
402.00	0.00	0.54	0.54	3.75	0.00	0.00	2.68	2.14	4.82

403.00	0.00	0.54	0.54	3.75	0.00	0.00	2.68	2.14	4.82
404.00	0.00	0.54	0.54	3.75	0.00	0.00	2.68	2.14	4.82
405.00	0.00	0.54	0.54	3.75	0.00	0.00	2.68	2.14	4.82
406.00	0.00	0.54	0.54	3.75	0.00	0.00	2.68	2.14	4.82
407.00	0.00	0.54	0.54	3.75	0.00	0.00	2.68	2.14	4.82
408.00	0.00	0.54	0.54	3.75	0.00	0.00	2.68	2.14	4.82
409.00	0.00	0.54	0.54	3.75	0.00	0.00	2.68	2.14	4.82
410.00	0.00	0.54	0.54	3.75	0.00	0.00	2.68	2.14	4.82
411.00	0.00	0.54	0.54	3.75	0.00	0.00	2.68	2.14	4.82
412.00	0.00	0.54	0.54	3.75	0.00	0.00	2.68	2.14	4.82
413.00	0.00	0.54	0.54	3.75	0.00	0.00	2.68	2.14	4.82
414.00	0.00	0.54	0.54	3.75	0.00	0.00	2.68	2.14	4.82
415.00	0.00	0.54	0.54	3.75	0.00	0.00	2.68	2.14	4.82
416.00	0.00	0.54	0.54	3.75	0.00	0.00	2.68	2.14	4.82
417.00	0.00	0.54	0.54	3.75	0.00	0.00	2.68	2.14	4.82
418.00	0.00	0.54	0.54	3.75	0.00	0.00	2.68	2.14	4.82
419.00	0.00	0.54	0.54	3.75	0.00	0.00	2.68	2.14	4.82
420.00	0.00	0.54	0.54	3.75	0.00	0.00	2.68	2.14	4.82
421.00	0.00	0.54	0.54	3.75	0.00	0.00	2.68	2.14	4.82
422.00	0.00	0.54	0.54	3.75	0.00	0.00	2.68	2.14	4.82
423.00	0.00	0.54	0.54	3.75	0.00	0.00	2.68	2.14	4.82
424.00	0.00	0.54	0.54	3.75	0.00	0.00	2.68	2.14	4.82
425.00	0.00	0.54	0.54	3.75	0.00	0.00	2.68	2.14	4.82
426.00	0.00	0.54	0.54	3.75	0.00	0.00	2.68	2.14	4.82
427.00	0.00	0.54	0.54	3.75	0.00	0.00	2.68	2.14	4.82
428.00	0.00	0.54	0.54	3.75	0.00	0.00	2.68	2.14	4.82
429.00	0.00	0.54	0.54	3.75	0.00	0.00	2.68	2.14	4.82
430.00	0.00	0.54	0.54	3.75	0.00	0.00	2.68	2.14	4.82
431.00	0.00	0.54	0.54	3.75	0.00	0.00	2.68	2.14	4.82
432.00	0.00	0.54	0.54	3.75	0.00	0.00	2.68	2.14	4.82
433.00	0.00	2.93	0.00	0.00	0.00	0.00	1.46	1.46	2.93
434.00	0.00	0.00	0.00	0.00	0.00	0.00	0.00	0.00	0.00

435.00	1.88	0.47	1.41	0.47	0.00	0.00	1.88	2.34	4.22
436.00	1.88	0.47	1.41	0.47	0.00	0.00	1.88	2.34	4.22
437.00	0.00	6.67	0.00	3.33	0.00	0.00	3.33	6.67	10.00
438.00	1.88	0.47	1.41	0.47	0.00	0.00	1.88	2.34	4.22
439.00	1.88	0.47	1.41	0.47	0.00	0.00	1.88	2.34	4.22
440.00	0.00	0.54	0.54	3.75	0.00	0.00	2.68	2.14	4.82
441.00	2.24	2.69	1.34	2.24	1.34	0.45	4.48	6.27	10.75
442.00	0.48	0.00	0.00	0.00	0.00	0.00	0.48	0.00	0.48
443.00	0.00	4.00	8.00	4.00	4.00	16.00	32.00	8.00	40.00
444.00	0.00	0.00	4.62	0.00	3.08	0.00	6.15	0.00	6.15
445.00	5.77	0.58	1.73	0.00	6.35	0.00	0.00	8.08	14.42
446.00	0.00	4.00	8.00	4.00	4.00	16.00	32.00	8.00	40.00
447.00	0.00	4.00	8.00	4.00	4.00	16.00	32.00	8.00	40.00
448.00	0.00	4.00	8.00	4.00	4.00	16.00	32.00	8.00	40.00
449.00	2.24	2.69	1.34	2.24	1.34	0.45	4.48	6.27	10.75
450.00	2.24	2.69	1.34	2.24	1.34	0.45	4.48	6.27	10.75
451.00	2.24	2.69	1.34	2.24	1.34	0.45	4.48	6.27	10.75
452.00	2.24	2.69	1.34	2.24	1.34	0.45	4.48	6.27	10.75
453.00	2.24	2.69	1.34	2.24	1.34	0.45	4.48	6.27	10.75
454.00	2.24	2.69	1.34	2.24	1.34	0.45	4.48	6.27	10.75
455.00	2.24	2.69	1.34	2.24	1.34	0.45	4.48	6.27	10.75
456.00	2.24	2.69	1.34	2.24	1.34	0.45	4.48	6.27	10.75
457.00	0.00	1.58	0.00	0.79	0.00	0.79	3.16	0.00	3.16
458.00	0.00	0.00	0.00	0.00	0.00	0.00	0.00	0.00	0.00
459.00	0.00	1.20	3.00	0.00	0.00	5.40	6.60	3.60	10.20
460.00	0.00	1.20	3.00	0.00	0.00	5.40	6.60	3.60	10.20
461.00	0.00	0.00	1.98	0.00	0.00	0.00	0.66	1.98	2.64
462.00	0.00	0.00	0.00	11.43	2.86	2.86	14.29	0.00	14.29
463.00	4.66	4.08	0.00	2.33	1.75	4.08	14.56	5.83	20.39
464.00	0.00	0.00	0.00	0.00	0.00	0.00	0.00	0.00	0.00
465.00	0.28	0.55	0.00	0.83	0.00	0.00	0.55	2.76	3.32
466.00	0.00	1.58	0.00	0.79	0.00	0.79	3.16	0.00	3.16

467.00	0.00	1.58	0.00	0.79	0.00	0.79	3.16	0.00	3.16
468.00	0.66	0.00	0.00	0.00	0.00	0.00	0.66	0.00	0.66
469.00	0.66	0.00	0.00	0.00	0.00	0.00	0.66	0.00	0.66
470.00	0.00	0.00	0.00	0.00	0.00	0.00	0.00	0.00	0.00
471.00	0.00	0.00	0.00	0.00	0.00	0.00	0.00	0.00	0.00
472.00	5.00	0.00	1.25	0.00	0.00	0.00	0.00	6.25	6.25
473.00	5.00	0.00	1.25	0.00	0.00	0.00	0.00	6.25	6.25
474.00	0.00	6.00	0.00	6.00	6.00	6.00	24.00	0.00	24.00
475.00	0.00	6.00	0.00	6.00	6.00	6.00	24.00	0.00	24.00
476.00	0.00	6.00	0.00	6.00	6.00	6.00	24.00	0.00	24.00
477.00	0.00	6.00	0.00	6.00	6.00	6.00	24.00	0.00	24.00
478.00	0.00	6.00	0.00	6.00	6.00	6.00	24.00	0.00	24.00
479.00	0.00	6.00	0.00	6.00	6.00	6.00	24.00	0.00	24.00
480.00	0.00	6.00	0.00	6.00	6.00	6.00	24.00	0.00	24.00
481.00	0.00	6.00	0.00	6.00	6.00	6.00	24.00	0.00	24.00
482.00	0.00	6.00	0.00	6.00	6.00	6.00	24.00	0.00	24.00
483.00	0.00	6.00	0.00	6.00	6.00	6.00	24.00	0.00	24.00
484.00	2.24	2.69	1.34	2.24	1.34	0.45	4.48	6.27	10.75
485.00	2.24	2.69	1.34	2.24	1.34	0.45	4.48	6.27	10.75
486.00	0.00	3.33	0.00	0.00	0.00	0.00	0.00	3.33	3.33
487.00	0.00	3.33	0.00	0.00	0.00	0.00	0.00	3.33	3.33
488.00	0.48	0.00	0.00	0.00	0.00	0.00	0.48	0.00	0.48
489.00	0.48	0.00	0.00	0.00	0.00	0.00	0.48	0.00	0.48
490.00	0.48	0.00	0.00	0.00	0.00	0.00	0.48	0.00	0.48
491.00	0.48	0.00	0.00	0.00	0.00	0.00	0.48	0.00	0.48
492.00	0.48	0.00	0.00	0.00	0.00	0.00	0.48	0.00	0.48
493.00	0.48	0.00	0.00	0.00	0.00	0.00	0.48	0.00	0.48
494.00	0.00	1.26	0.00	0.00	0.00	0.00	1.26	0.00	1.26
495.00	1.88	0.47	1.41	0.47	0.00	0.00	1.88	2.34	4.22
496.00	0.66	0.00	0.00	0.00	0.00	0.00	0.66	0.00	0.66
497.00	1.88	0.47	1.41	0.47	0.00	0.00	1.88	2.34	4.22
498.00	1.88	0.47	1.41	0.47	0.00	0.00	1.88	2.34	4.22

499.00	0.66	0.00	0.00	0.00	0.00	0.00	0.66	0.00	0.66
500.00	0.00	1.20	3.00	0.00	0.00	5.40	6.60	3.60	10.20
501.00	0.00	1.18	0.00	0.00	0.00	0.00	8.24	0.00	2.35
502.00	1.98	0.66	0.00	0.00	1.32	0.00	0.66	3.30	3.96
503.00	0.77	0.00	0.00	0.00	0.00	0.00	0.00	0.77	0.77
504.00	0.00	0.00	0.00	0.00	0.00	0.00	0.00	0.00	0.00
505.00	1.48	0.00	0.00	0.00	0.49	0.00	2.46	1.97	4.43
506.00	0.00	1.57	0.00	0.26	0.00	0.00	1.57	0.78	2.35
507.00	0.00	0.00	0.00	0.00	0.00	0.00	0.00	0.00	0.00
508.00	1.48	0.00	0.00	0.00	0.49	0.00	2.46	1.97	4.43
509.00	1.86	0.47	0.00	2.33	0.00	2.33	5.58	1.40	6.98
510.00	0.00	0.00	0.94	0.00	0.00	0.00	0.00	0.94	0.94
511.00	0.00	0.00	0.94	0.00	0.00	0.00	0.00	0.94	0.94
512.00	5.77	0.58	1.73	0.00	6.35	0.00	6.35	8.08	14.42
513.00	0.00	1.57	0.00	0.26	0.00	0.00	1.57	0.78	2.35
514.00	0.65	1.30	0.00	0.00	0.00	0.00	0.65	1.30	1.96
515.00	3.33	3.33	0.00	0.00	0.00	0.00	3.33	3.33	6.67
516.00	0.49	2.44	0.00	2.44	0.00	0.49	3.90	2.44	6.34
517.00	1.19	2.38	0.59	2.97	0.00	1.78	4.16	5.94	10.10
518.00	0.00	5.00	0.00	0.00	1.67	0.00	10.00	3.33	13.33
519.00	1.63	6.73	0.82	0.82	0.82	0.20	5.51	6.33	11.84
520.00	1.63	6.73	0.82	0.82	0.82	0.20	5.51	6.33	11.84
521.00	1.63	6.73	0.82	0.82	0.82	0.20	5.51	6.33	11.84
522.00	0.00	1.57	0.00	0.26	0.00	0.00	1.57	0.78	2.35
523.00	0.90	4.51	0.90	6.77	0.00	0.00	3.61	10.38	13.98
524.00	1.63	6.73	0.82	0.82	0.82	0.20	5.51	6.33	11.84
525.00	3.00	3.30	0.00	4.20	0.90	2.40	9.30	10.20	19.50
526.00	0.28	0.55	0.00	0.83	0.00	0.00	0.55	2.76	3.32
527.00	0.00	1.18	0.00	0.00	0.00	0.00	8.24	0.00	2.35
528.00	0.90	4.51	0.90	6.77	0.00	0.00	3.61	10.38	13.98
529.00	0.00	1.26	0.00	0.00	0.00	0.00	1.26	0.00	1.26
530.00	0.00	1.26	0.00	0.00	0.00	0.00	1.26	0.00	1.26

531.00	1.63	6.73	0.82	0.82	0.82	0.20	5.51	6.33	11.84
532.00	1.63	6.73	0.82	0.82	0.82	0.20	5.51	6.33	11.84
533.00	1.48	0.00	0.00	0.00	0.49	0.00	2.46	1.97	4.43
534.00	1.48	0.00	0.00	0.00	0.49	0.00	2.46	1.97	4.43
535.00	3.00	3.30	0.00	4.20	0.90	2.40	9.30	10.20	19.50
536.00	0.00	0.00	0.00	0.00	0.00	0.00	1.68	1.12	2.80
537.00	0.00	0.00	0.00	0.00	0.00	0.00	1.68	1.12	2.80
538.00	0.00	0.00	0.00	0.00	0.00	0.00	0.00	0.00	0.00
539.00	0.00	1.57	0.00	0.26	0.00	0.00	1.57	0.78	2.35
540.00	0.00	1.57	0.00	0.26	0.00	0.00	1.57	0.78	2.35
541.00	0.00	1.57	0.00	0.26	0.00	0.00	1.57	0.78	2.35
542.00	1.98	0.66	0.00	0.00	1.32	0.00	0.66	3.30	3.96
543.00	1.98	0.66	0.00	0.00	1.32	0.00	0.66	3.30	3.96
544.00	0.00	1.26	0.00	0.00	0.00	0.00	1.26	0.00	1.26
545.00	0.00	0.54	0.54	3.75	0.00	0.00	2.68	2.14	4.82
