# Supplementary material for: Convergence and divergence in gesture repertoires as an adaptive mechanism for social bonding in primates
Source: R Soc Open Sci. 2017 Nov 29;4(11):170181. doi: 10.1098/rsos.170181 (PMC5717623; doi:10.1098/rsos.170181)
Supplement: Supplementary Information 6 [file rsos170181supp6.pdf]

Convergence and divergence in gesture repertoires as an adaptive mechanism for social bonding in primates

Anna Ilona Roberts, Sam George Bradley Roberts

Royal Society Open Science

Supplementary Information 6

GLMM models dataset

matching number	Repertoire size homogeneous gesture	Repertoire size heterogeneous gesture	Repertoire size homogeneous visual gesture	Repertoire size homogeneous tactile gesture	Repertoire size homogeneous auditory short range gesture	Repertoire size homogeneous auditory long range gesture	Repertoire size heterogeneous visual gesture	Repertoire size heterogeneous tactile gesture	Repertoire size heterogeneous auditory short range gesture	Repertoire size heterogeneous auditory long range gesture
1.00	1.00	2.00	1.00	0.00	0.00	0.00	1.00	1.00	0.00	0.00
2.00	1.00	1.00	1.00	0.00	0.00	0.00	1.00	0.00	0.00	0.00
3.00	999.00	999.00	999.00	999.00	999.00	999.00	999.00	999.00	999.00	999.00
4.00	1.00	0.00	0.00	0.00	1.00	0.00	0.00	0.00	0.00	0.00
5.00	0.00	2.00	0.00	0.00	0.00	0.00	1.00	0.00	1.00	0.00
6.00	1.00	0.00	0.00	0.00	1.00	0.00	0.00	0.00	0.00	0.00
7.00	1.00	0.00	0.00	0.00	1.00	0.00	0.00	0.00	0.00	0.00
8.00	999.00	999.00	999.00	999.00	999.00	999.00	999.00	999.00	999.00	999.00
9.00	3.00	1.00	1.00	0.00	0.00	2.00	1.00	0.00	0.00	0.00
10.00	1.00	3.00	1.00	0.00	0.00	0.00	1.00	0.00	0.00	2.00
11.00	1.00	0.00	0.00	0.00	1.00	0.00	0.00	0.00	0.00	0.00
12.00	2.00	0.00	2.00	0.00	0.00	0.00	0.00	0.00	0.00	0.00

13.00	1.00	2.00	1.00	0.00	0.00	0.00	2.00	0.00	0.00	0.00
14.00	3.00	0.00	3.00	0.00	0.00	0.00	0.00	0.00	0.00	0.00
15.00	1.00	0.00	0.00	1.00	0.00	0.00	0.00	0.00	0.00	0.00
16.00	1.00	0.00	0.00	1.00	0.00	0.00	0.00	0.00	0.00	0.00
17.00	0.00	2.00	0.00	0.00	0.00	0.00	0.00	2.00	0.00	0.00
18.00	2.00	0.00	1.00	1.00	0.00	0.00	0.00	0.00	0.00	0.00
19.00	1.00	1.00	1.00	0.00	0.00	0.00	1.00	0.00	0.00	0.00
20.00	1.00	1.00	1.00	0.00	0.00	0.00	1.00	0.00	0.00	0.00
21.00	1.00	0.00	1.00	0.00	0.00	0.00	0.00	0.00	0.00	0.00
22.00	3.00	0.00	2.00	0.00	0.00	1.00	0.00	0.00	0.00	0.00
23.00	1.00	0.00	0.00	0.00	1.00	0.00	0.00	0.00	0.00	0.00
24.00	1.00	0.00	0.00	0.00	1.00	0.00	0.00	0.00	0.00	0.00
25.00	2.00	0.00	1.00	1.00	0.00	0.00	0.00	0.00	0.00	0.00
26.00	2.00	0.00	1.00	0.00	0.00	1.00	0.00	0.00	0.00	0.00
27.00	1.00	1.00	1.00	0.00	0.00	0.00	0.00	1.00	0.00	0.00
28.00	2.00	0.00	0.00	2.00	0.00	0.00	0.00	0.00	0.00	0.00
29.00	1.00	1.00	1.00	0.00	0.00	0.00	1.00	0.00	0.00	0.00
30.00	999.00	999.00	999.00	999.00	999.00	999.00	999.00	999.00	999.00	999.00
31.00	2.00	0.00	0.00	0.00	1.00	1.00	0.00	0.00	0.00	0.00
32.00	1.00	1.00	0.00	0.00	0.00	1.00	1.00	0.00	0.00	0.00
33.00	999.00	999.00	999.00	999.00	999.00	999.00	999.00	999.00	999.00	999.00
34.00	2.00	1.00	2.00	0.00	0.00	0.00	0.00	1.00	0.00	0.00
35.00	3.00	1.00	1.00	2.00	0.00	0.00	1.00	0.00	0.00	0.00
36.00	1.00	2.00	0.00	1.00	0.00	0.00	0.00	2.00	0.00	0.00
37.00	1.00	1.00	1.00	0.00	0.00	0.00	1.00	0.00	0.00	0.00
38.00	999.00	999.00	999.00	999.00	999.00	999.00	999.00	999.00	999.00	999.00
39.00	2.00	0.00	0.00	2.00	0.00	0.00	0.00	0.00	0.00	0.00
40.00	1.00	1.00	1.00	0.00	0.00	0.00	1.00	0.00	0.00	0.00
41.00	1.00	1.00	1.00	0.00	0.00	0.00	1.00	0.00	0.00	0.00
42.00	3.00	0.00	1.00	0.00	0.00	2.00	0.00	0.00	0.00	0.00
43.00	3.00	0.00	2.00	0.00	0.00	1.00	0.00	0.00	0.00	0.00
44.00	999.00	999.00	999.00	999.00	999.00	999.00	999.00	999.00	999.00	999.00

45.00	999.00	999.00	999.00	999.00	999.00	999.00	999.00	999.00	999.00	999.00
46.00	2.00	1.00	1.00	0.00	0.00	1.00	1.00	0.00	0.00	0.00
47.00	3.00	0.00	2.00	0.00	0.00	1.00	0.00	0.00	0.00	0.00
48.00	999.00	999.00	999.00	999.00	999.00	999.00	999.00	999.00	999.00	999.00
49.00	4.00	0.00	1.00	3.00	0.00	0.00	0.00	0.00	0.00	0.00
50.00	999.00	999.00	999.00	999.00	999.00	999.00	999.00	999.00	999.00	999.00
51.00	2.00	0.00	1.00	0.00	1.00	0.00	0.00	0.00	0.00	0.00
52.00	999.00	999.00	999.00	999.00	999.00	999.00	999.00	999.00	999.00	999.00
53.00	1.00	1.00	0.00	0.00	0.00	1.00	1.00	0.00	0.00	0.00
54.00	2.00	0.00	1.00	0.00	0.00	1.00	0.00	0.00	0.00	0.00
55.00	3.00	0.00	2.00	0.00	0.00	1.00	0.00	0.00	0.00	0.00
56.00	2.00	0.00	1.00	0.00	0.00	1.00	0.00	0.00	0.00	0.00
57.00	2.00	0.00	1.00	0.00	0.00	1.00	0.00	0.00	0.00	0.00
58.00	2.00	0.00	1.00	0.00	0.00	1.00	0.00	0.00	0.00	0.00
59.00	3.00	0.00	2.00	0.00	0.00	1.00	0.00	0.00	0.00	0.00
60.00	2.00	0.00	1.00	0.00	0.00	1.00	0.00	0.00	0.00	0.00
61.00	999.00	999.00	999.00	999.00	999.00	999.00	999.00	999.00	999.00	999.00
62.00	999.00	999.00	999.00	999.00	999.00	999.00	999.00	999.00	999.00	999.00
63.00	6.00	1.00	4.00	2.00	0.00	0.00	0.00	1.00	0.00	0.00
64.00	3.00	0.00	3.00	0.00	0.00	0.00	0.00	0.00	0.00	0.00
65.00	0.00	4.00	0.00	0.00	0.00	0.00	4.00	0.00	0.00	0.00
66.00	1.00	1.00	1.00	0.00	0.00	0.00	0.00	1.00	0.00	0.00
67.00	2.00	2.00	2.00	0.00	0.00	0.00	0.00	0.00	0.00	2.00
68.00	999.00	999.00	999.00	999.00	999.00	999.00	999.00	999.00	999.00	999.00
69.00	2.00	1.00	2.00	0.00	0.00	0.00	1.00	0.00	0.00	0.00
70.00	2.00	1.00	1.00	0.00	1.00	0.00	0.00	0.00	0.00	1.00
71.00	1.00	2.00	1.00	0.00	0.00	0.00	2.00	0.00	0.00	0.00
72.00	2.00	1.00	2.00	0.00	0.00	0.00	1.00	0.00	0.00	0.00
73.00	3.00	0.00	3.00	0.00	0.00	0.00	0.00	0.00	0.00	0.00
74.00	1.00	1.00	1.00	0.00	0.00	0.00	1.00	0.00	0.00	0.00
75.00	3.00	2.00	1.00	2.00	0.00	0.00	0.00	2.00	0.00	0.00
76.00	1.00	2.00	1.00	0.00	0.00	0.00	2.00	0.00	0.00	0.00

77.00	2.00	0.00	2.00	0.00	0.00	0.00	0.00	0.00	0.00	0.00
78.00	1.00	1.00	1.00	0.00	0.00	0.00	1.00	0.00	0.00	0.00
79.00	2.00	0.00	1.00	0.00	0.00	1.00	0.00	0.00	0.00	0.00
80.00	999.00	999.00	999.00	999.00	999.00	999.00	999.00	999.00	999.00	999.00
81.00	1.00	1.00	1.00	0.00	0.00	0.00	1.00	0.00	0.00	0.00
82.00	2.00	0.00	2.00	0.00	0.00	0.00	0.00	0.00	0.00	0.00
83.00	999.00	999.00	999.00	999.00	999.00	999.00	999.00	999.00	999.00	999.00
84.00	0.00	3.00	0.00	0.00	0.00	0.00	3.00	0.00	0.00	0.00
85.00	1.00	1.00	1.00	0.00	0.00	0.00	1.00	0.00	0.00	0.00
86.00	2.00	0.00	2.00	0.00	0.00	0.00	0.00	0.00	0.00	0.00
87.00	2.00	0.00	1.00	1.00	0.00	0.00	0.00	0.00	0.00	0.00
88.00	1.00	0.00	1.00	0.00	0.00	0.00	0.00	0.00	0.00	0.00
89.00	999.00	999.00	999.00	999.00	999.00	999.00	999.00	999.00	999.00	999.00
90.00	2.00	0.00	1.00	1.00	0.00	0.00	0.00	0.00	0.00	0.00
91.00	2.00	0.00	2.00	0.00	0.00	0.00	0.00	0.00	0.00	0.00
92.00	2.00	0.00	1.00	1.00	0.00	0.00	0.00	0.00	0.00	0.00
93.00	2.00	0.00	2.00	0.00	0.00	0.00	0.00	0.00	0.00	0.00
94.00	1.00	1.00	0.00	0.00	0.00	1.00	1.00	0.00	0.00	0.00
95.00	2.00	0.00	2.00	0.00	0.00	0.00	0.00	0.00	0.00	0.00
96.00	1.00	0.00	1.00	0.00	0.00	0.00	0.00	0.00	0.00	0.00
97.00	2.00	0.00	1.00	1.00	0.00	0.00	0.00	0.00	0.00	0.00
98.00	1.00	1.00	1.00	0.00	0.00	0.00	1.00	0.00	0.00	0.00
99.00	2.00	0.00	2.00	0.00	0.00	0.00	0.00	0.00	0.00	0.00
100.00	2.00	0.00	2.00	0.00	0.00	0.00	0.00	0.00	0.00	0.00
101.00	2.00	1.00	1.00	1.00	0.00	0.00	1.00	0.00	0.00	0.00
102.00	999.00	999.00	999.00	999.00	999.00	999.00	999.00	999.00	999.00	999.00
103.00	999.00	999.00	999.00	999.00	999.00	999.00	999.00	999.00	999.00	999.00
104.00	999.00	999.00	999.00	999.00	999.00	999.00	999.00	999.00	999.00	999.00
105.00	1.00	2.00	1.00	0.00	0.00	0.00	2.00	0.00	0.00	0.00
106.00	999.00	999.00	999.00	999.00	999.00	999.00	999.00	999.00	999.00	999.00
107.00	1.00	2.00	1.00	0.00	0.00	0.00	2.00	0.00	0.00	0.00
108.00	3.00	0.00	3.00	0.00	0.00	0.00	0.00	0.00	0.00	0.00

109.00	999.00	999.00	999.00	999.00	999.00	999.00	999.00	999.00	999.00	999.00
110.00	2.00	6.00	1.00	0.00	0.00	1.00	2.00	0.00	0.00	4.00
111.00	3.00	4.00	2.00	0.00	0.00	1.00	2.00	0.00	0.00	2.00
112.00	0.00	9.00	0.00	0.00	0.00	0.00	4.00	0.00	0.00	5.00
113.00	3.00	4.00	1.00	0.00	1.00	1.00	2.00	0.00	0.00	2.00
114.00	5.00	0.00	4.00	0.00	0.00	1.00	0.00	0.00	0.00	0.00
115.00	5.00	0.00	2.00	0.00	0.00	3.00	0.00	0.00	0.00	0.00
116.00	999.00	999.00	999.00	999.00	999.00	999.00	999.00	999.00	999.00	999.00
117.00	0.00	5.00	0.00	0.00	0.00	0.00	3.00	0.00	0.00	2.00
118.00	3.00	0.00	2.00	0.00	0.00	1.00	0.00	0.00	0.00	0.00
119.00	2.00	4.00	2.00	0.00	0.00	0.00	1.00	0.00	0.00	3.00
120.00	999.00	999.00	999.00	999.00	999.00	999.00	999.00	999.00	999.00	999.00
121.00	999.00	999.00	999.00	999.00	999.00	999.00	999.00	999.00	999.00	999.00
122.00	3.00	0.00	1.00	0.00	0.00	2.00	0.00	0.00	0.00	0.00
123.00	999.00	999.00	999.00	999.00	999.00	999.00	999.00	999.00	999.00	999.00
124.00	0.00	2.00	0.00	0.00	0.00	0.00	2.00	0.00	0.00	0.00
125.00	0.00	2.00	0.00	0.00	0.00	0.00	2.00	0.00	0.00	0.00
126.00	0.00	2.00	0.00	0.00	0.00	0.00	1.00	1.00	0.00	0.00
127.00	0.00	4.00	0.00	0.00	0.00	0.00	2.00	0.00	0.00	2.00
128.00	2.00	0.00	0.00	0.00	0.00	2.00	0.00	0.00	0.00	0.00
129.00	3.00	0.00	2.00	0.00	0.00	1.00	0.00	0.00	0.00	0.00
130.00	2.00	0.00	0.00	0.00	0.00	2.00	0.00	0.00	0.00	0.00
131.00	3.00	0.00	1.00	2.00	0.00	0.00	0.00	0.00	0.00	0.00
132.00	1.00	2.00	0.00	0.00	0.00	1.00	0.00	0.00	0.00	2.00
133.00	999.00	999.00	999.00	999.00	999.00	999.00	999.00	999.00	999.00	999.00
134.00	999.00	999.00	999.00	999.00	999.00	999.00	999.00	999.00	999.00	999.00
135.00	3.00	0.00	1.00	0.00	0.00	2.00	0.00	0.00	0.00	0.00
136.00	999.00	999.00	999.00	999.00	999.00	999.00	999.00	999.00	999.00	999.00
137.00	1.00	0.00	0.00	0.00	1.00	0.00	0.00	0.00	0.00	0.00
138.00	999.00	999.00	999.00	999.00	999.00	999.00	999.00	999.00	999.00	999.00
139.00	999.00	999.00	999.00	999.00	999.00	999.00	999.00	999.00	999.00	999.00
140.00	999.00	999.00	999.00	999.00	999.00	999.00	999.00	999.00	999.00	999.00

141.00	0.00	3.00	0.00	0.00	0.00	0.00	2.00	0.00	1.00	0.00
142.00	999.00	999.00	999.00	999.00	999.00	999.00	999.00	999.00	999.00	999.00
143.00	999.00	999.00	999.00	999.00	999.00	999.00	999.00	999.00	999.00	999.00
144.00	999.00	999.00	999.00	999.00	999.00	999.00	999.00	999.00	999.00	999.00
145.00	0.00	2.00	0.00	0.00	0.00	0.00	1.00	0.00	0.00	1.00
146.00	0.00	3.00	0.00	0.00	0.00	0.00	1.00	1.00	0.00	1.00
147.00	1.00	1.00	0.00	0.00	0.00	1.00	0.00	0.00	1.00	0.00
148.00	1.00	2.00	0.00	0.00	1.00	0.00	2.00	0.00	0.00	0.00
149.00	0.00	2.00	0.00	0.00	0.00	0.00	1.00	0.00	0.00	1.00
150.00	1.00	2.00	1.00	0.00	0.00	0.00	2.00	0.00	0.00	0.00
151.00	2.00	0.00	1.00	1.00	0.00	0.00	0.00	0.00	0.00	0.00
152.00	2.00	0.00	2.00	0.00	0.00	0.00	0.00	0.00	0.00	0.00
153.00	2.00	0.00	2.00	0.00	0.00	0.00	0.00	0.00	0.00	0.00
154.00	2.00	0.00	2.00	0.00	0.00	0.00	0.00	0.00	0.00	0.00
155.00	999.00	999.00	999.00	999.00	999.00	999.00	999.00	999.00	999.00	999.00
156.00	2.00	0.00	2.00	0.00	0.00	0.00	0.00	0.00	0.00	0.00
157.00	2.00	0.00	2.00	0.00	0.00	0.00	0.00	0.00	0.00	0.00
158.00	2.00	0.00	2.00	0.00	0.00	0.00	0.00	0.00	0.00	0.00
159.00	2.00	0.00	2.00	0.00	0.00	0.00	0.00	0.00	0.00	0.00
160.00	2.00	0.00	1.00	1.00	0.00	0.00	0.00	0.00	0.00	0.00
161.00	1.00	0.00	0.00	0.00	0.00	1.00	0.00	0.00	0.00	0.00
162.00	1.00	0.00	0.00	0.00	0.00	1.00	0.00	0.00	0.00	0.00
163.00	1.00	0.00	0.00	0.00	1.00	0.00	0.00	0.00	0.00	0.00
164.00	0.00	1.00	0.00	0.00	0.00	0.00	0.00	0.00	1.00	0.00
165.00	1.00	0.00	1.00	0.00	0.00	0.00	0.00	0.00	0.00	0.00
166.00	1.00	0.00	0.00	0.00	0.00	1.00	0.00	0.00	0.00	0.00
167.00	1.00	0.00	0.00	0.00	0.00	1.00	0.00	0.00	0.00	0.00
168.00	0.00	1.00	0.00	0.00	0.00	0.00	0.00	0.00	1.00	0.00
169.00	999.00	999.00	999.00	999.00	999.00	999.00	999.00	999.00	999.00	999.00
170.00	999.00	999.00	999.00	999.00	999.00	999.00	999.00	999.00	999.00	999.00
171.00	999.00	999.00	999.00	999.00	999.00	999.00	999.00	999.00	999.00	999.00
172.00	999.00	999.00	999.00	999.00	999.00	999.00	999.00	999.00	999.00	999.00

173.00	999.00	999.00	999.00	999.00	999.00	999.00	999.00	999.00	999.00	999.00
174.00	999.00	999.00	999.00	999.00	999.00	999.00	999.00	999.00	999.00	999.00
175.00	999.00	999.00	999.00	999.00	999.00	999.00	999.00	999.00	999.00	999.00
176.00	999.00	999.00	999.00	999.00	999.00	999.00	999.00	999.00	999.00	999.00
177.00	1.00	0.00	1.00	0.00	0.00	0.00	0.00	0.00	0.00	0.00
178.00	1.00	0.00	1.00	0.00	0.00	0.00	0.00	0.00	0.00	0.00
179.00	1.00	0.00	1.00	0.00	0.00	0.00	0.00	0.00	0.00	0.00
180.00	1.00	0.00	0.00	0.00	1.00	0.00	0.00	0.00	0.00	0.00
181.00	1.00	0.00	1.00	0.00	0.00	0.00	0.00	0.00	0.00	0.00
182.00	1.00	0.00	0.00	0.00	1.00	0.00	0.00	0.00	0.00	0.00
183.00	1.00	0.00	1.00	0.00	0.00	0.00	0.00	0.00	0.00	0.00
184.00	999.00	999.00	999.00	999.00	999.00	999.00	999.00	999.00	999.00	999.00
185.00	999.00	999.00	999.00	999.00	999.00	999.00	999.00	999.00	999.00	999.00
186.00	1.00	0.00	0.00	0.00	1.00	0.00	0.00	0.00	0.00	0.00
187.00	1.00	0.00	1.00	0.00	0.00	0.00	0.00	0.00	0.00	0.00
188.00	1.00	0.00	1.00	0.00	0.00	0.00	0.00	0.00	0.00	0.00
189.00	0.00	1.00	0.00	0.00	0.00	0.00	1.00	0.00	0.00	0.00
190.00	0.00	1.00	0.00	0.00	0.00	0.00	1.00	0.00	0.00	0.00
191.00	0.00	1.00	0.00	0.00	0.00	0.00	1.00	0.00	0.00	0.00
192.00	0.00	1.00	0.00	0.00	0.00	0.00	1.00	0.00	0.00	0.00
193.00	999.00	999.00	999.00	999.00	999.00	999.00	999.00	999.00	999.00	999.00
194.00	999.00	999.00	999.00	999.00	999.00	999.00	999.00	999.00	999.00	999.00
195.00	1.00	0.00	1.00	0.00	0.00	0.00	0.00	0.00	0.00	0.00
196.00	1.00	0.00	1.00	0.00	0.00	0.00	0.00	0.00	0.00	0.00
197.00	1.00	0.00	0.00	0.00	1.00	0.00	0.00	0.00	0.00	0.00
198.00	1.00	0.00	0.00	0.00	1.00	0.00	0.00	0.00	0.00	0.00
199.00	1.00	0.00	1.00	0.00	0.00	0.00	0.00	0.00	0.00	0.00
200.00	1.00	0.00	1.00	0.00	0.00	0.00	0.00	0.00	0.00	0.00
201.00	1.00	0.00	1.00	0.00	0.00	0.00	0.00	0.00	0.00	0.00
202.00	1.00	0.00	1.00	0.00	0.00	0.00	0.00	0.00	0.00	0.00
203.00	1.00	0.00	0.00	0.00	1.00	0.00	0.00	0.00	0.00	0.00
204.00	1.00	0.00	0.00	0.00	1.00	0.00	0.00	0.00	0.00	0.00

205.00	1.00	0.00	0.00	0.00	1.00	0.00	0.00	0.00	0.00	0.00
206.00	1.00	0.00	0.00	0.00	1.00	0.00	0.00	0.00	0.00	0.00
207.00	1.00	0.00	0.00	0.00	1.00	0.00	0.00	0.00	0.00	0.00
208.00	1.00	0.00	0.00	0.00	1.00	0.00	0.00	0.00	0.00	0.00
209.00	1.00	0.00	0.00	0.00	1.00	0.00	0.00	0.00	0.00	0.00
210.00	1.00	0.00	0.00	0.00	1.00	0.00	0.00	0.00	0.00	0.00
211.00	1.00	0.00	0.00	0.00	1.00	0.00	0.00	0.00	0.00	0.00
212.00	1.00	0.00	0.00	0.00	1.00	0.00	0.00	0.00	0.00	0.00
213.00	1.00	0.00	0.00	0.00	1.00	0.00	0.00	0.00	0.00	0.00
214.00	1.00	0.00	0.00	0.00	1.00	0.00	0.00	0.00	0.00	0.00
215.00	1.00	0.00	1.00	0.00	0.00	0.00	0.00	0.00	0.00	0.00
216.00	1.00	0.00	0.00	0.00	1.00	0.00	0.00	0.00	0.00	0.00
217.00	1.00	0.00	0.00	0.00	1.00	0.00	0.00	0.00	0.00	0.00
218.00	1.00	0.00	0.00	0.00	1.00	0.00	0.00	0.00	0.00	0.00
219.00	1.00	0.00	0.00	0.00	1.00	0.00	0.00	0.00	0.00	0.00
220.00	1.00	0.00	0.00	0.00	1.00	0.00	0.00	0.00	0.00	0.00
221.00	1.00	0.00	0.00	0.00	1.00	0.00	0.00	0.00	0.00	0.00
222.00	1.00	0.00	0.00	0.00	1.00	0.00	0.00	0.00	0.00	0.00
223.00	1.00	0.00	0.00	0.00	1.00	0.00	0.00	0.00	0.00	0.00
224.00	1.00	0.00	0.00	0.00	1.00	0.00	0.00	0.00	0.00	0.00
225.00	1.00	0.00	0.00	0.00	1.00	0.00	0.00	0.00	0.00	0.00
226.00	1.00	0.00	0.00	0.00	1.00	0.00	0.00	0.00	0.00	0.00
227.00	1.00	0.00	0.00	0.00	1.00	0.00	0.00	0.00	0.00	0.00
228.00	1.00	0.00	0.00	0.00	1.00	0.00	0.00	0.00	0.00	0.00
229.00	1.00	0.00	0.00	0.00	1.00	0.00	0.00	0.00	0.00	0.00
230.00	1.00	0.00	0.00	0.00	1.00	0.00	0.00	0.00	0.00	0.00
231.00	1.00	0.00	0.00	0.00	1.00	0.00	0.00	0.00	0.00	0.00
232.00	1.00	0.00	0.00	0.00	1.00	0.00	0.00	0.00	0.00	0.00
233.00	1.00	0.00	0.00	0.00	1.00	0.00	0.00	0.00	0.00	0.00
234.00	1.00	0.00	0.00	0.00	1.00	0.00	0.00	0.00	0.00	0.00
235.00	1.00	0.00	0.00	0.00	1.00	0.00	0.00	0.00	0.00	0.00
236.00	1.00	0.00	0.00	0.00	1.00	0.00	0.00	0.00	0.00	0.00

237.00	1.00	0.00	0.00	0.00	1.00	0.00	0.00	0.00	0.00	0.00
238.00	1.00	0.00	0.00	0.00	1.00	0.00	0.00	0.00	0.00	0.00
239.00	1.00	0.00	1.00	0.00	0.00	0.00	0.00	0.00	0.00	0.00
240.00	1.00	0.00	0.00	0.00	1.00	0.00	0.00	0.00	0.00	0.00
241.00	1.00	0.00	0.00	0.00	1.00	0.00	0.00	0.00	0.00	0.00
242.00	1.00	0.00	1.00	0.00	0.00	0.00	0.00	0.00	0.00	0.00
243.00	1.00	0.00	1.00	0.00	0.00	0.00	0.00	0.00	0.00	0.00
244.00	1.00	1.00	1.00	0.00	0.00	0.00	1.00	0.00	0.00	0.00
245.00	1.00	0.00	1.00	0.00	0.00	0.00	0.00	0.00	0.00	0.00
246.00	1.00	0.00	0.00	0.00	1.00	0.00	0.00	0.00	0.00	0.00
247.00	1.00	0.00	1.00	0.00	0.00	0.00	0.00	0.00	0.00	0.00
248.00	1.00	0.00	1.00	0.00	0.00	0.00	0.00	0.00	0.00	0.00
249.00	1.00	0.00	1.00	0.00	0.00	0.00	0.00	0.00	0.00	0.00
250.00	1.00	0.00	0.00	0.00	1.00	0.00	0.00	0.00	0.00	0.00
251.00	1.00	0.00	0.00	0.00	1.00	0.00	0.00	0.00	0.00	0.00
252.00	0.00	1.00	0.00	0.00	0.00	0.00	1.00	0.00	0.00	0.00
253.00	0.00	1.00	0.00	0.00	0.00	0.00	1.00	0.00	0.00	0.00
254.00	0.00	1.00	0.00	0.00	0.00	0.00	1.00	0.00	0.00	0.00
255.00	1.00	0.00	1.00	0.00	0.00	0.00	0.00	0.00	0.00	0.00
256.00	1.00	0.00	1.00	0.00	0.00	0.00	0.00	0.00	0.00	0.00
257.00	0.00	1.00	0.00	0.00	0.00	0.00	1.00	0.00	0.00	0.00
258.00	999.00	999.00	999.00	999.00	999.00	999.00	999.00	999.00	999.00	999.00
259.00	999.00	999.00	999.00	999.00	999.00	999.00	999.00	999.00	999.00	999.00
260.00	999.00	999.00	999.00	999.00	999.00	999.00	999.00	999.00	999.00	999.00
261.00	999.00	999.00	999.00	999.00	999.00	999.00	999.00	999.00	999.00	999.00
262.00	999.00	999.00	999.00	999.00	999.00	999.00	999.00	999.00	999.00	999.00
263.00	999.00	999.00	999.00	999.00	999.00	999.00	999.00	999.00	999.00	999.00
264.00	1.00	0.00	1.00	0.00	0.00	0.00	0.00	0.00	0.00	0.00
265.00	1.00	0.00	1.00	0.00	0.00	0.00	0.00	0.00	0.00	0.00
266.00	1.00	0.00	1.00	0.00	0.00	0.00	0.00	0.00	0.00	0.00
267.00	1.00	0.00	0.00	0.00	1.00	0.00	0.00	0.00	0.00	0.00
268.00	1.00	0.00	0.00	0.00	1.00	0.00	0.00	0.00	0.00	0.00

269.00	1.00	0.00	0.00	0.00	1.00	0.00	0.00	0.00	0.00	0.00
270.00	1.00	0.00	0.00	0.00	1.00	0.00	0.00	0.00	0.00	0.00
271.00	1.00	0.00	0.00	0.00	1.00	0.00	0.00	0.00	0.00	0.00
272.00	1.00	0.00	0.00	0.00	1.00	0.00	0.00	0.00	0.00	0.00
273.00	1.00	0.00	0.00	0.00	1.00	0.00	0.00	0.00	0.00	0.00
274.00	1.00	0.00	0.00	0.00	1.00	0.00	0.00	0.00	0.00	0.00
275.00	1.00	0.00	0.00	0.00	1.00	0.00	0.00	0.00	0.00	0.00
276.00	1.00	0.00	0.00	0.00	1.00	0.00	0.00	0.00	0.00	0.00
277.00	1.00	0.00	0.00	0.00	1.00	0.00	0.00	0.00	0.00	0.00
278.00	1.00	0.00	1.00	0.00	0.00	0.00	0.00	0.00	0.00	0.00
279.00	1.00	0.00	1.00	0.00	0.00	0.00	0.00	0.00	0.00	0.00
280.00	1.00	0.00	1.00	0.00	0.00	0.00	0.00	0.00	0.00	0.00
281.00	1.00	0.00	0.00	1.00	0.00	0.00	0.00	0.00	0.00	0.00
282.00	999.00	999.00	999.00	999.00	999.00	999.00	999.00	999.00	999.00	999.00
283.00	999.00	999.00	999.00	999.00	999.00	999.00	999.00	999.00	999.00	999.00
284.00	0.00	1.00	0.00	0.00	0.00	0.00	0.00	1.00	0.00	0.00
285.00	1.00	0.00	0.00	1.00	0.00	0.00	0.00	0.00	0.00	0.00
286.00	1.00	0.00	0.00	1.00	0.00	0.00	0.00	0.00	0.00	0.00
287.00	1.00	0.00	1.00	0.00	0.00	0.00	0.00	0.00	0.00	0.00
288.00	999.00	999.00	999.00	999.00	999.00	999.00	999.00	999.00	999.00	999.00
289.00	999.00	999.00	999.00	999.00	999.00	999.00	999.00	999.00	999.00	999.00
290.00	999.00	999.00	999.00	999.00	999.00	999.00	999.00	999.00	999.00	999.00
291.00	999.00	999.00	999.00	999.00	999.00	999.00	999.00	999.00	999.00	999.00
292.00	999.00	999.00	999.00	999.00	999.00	999.00	999.00	999.00	999.00	999.00
293.00	0.00	1.00	0.00	0.00	0.00	0.00	1.00	0.00	0.00	0.00
294.00	999.00	999.00	999.00	999.00	999.00	999.00	999.00	999.00	999.00	999.00
295.00	1.00	0.00	1.00	0.00	0.00	0.00	0.00	0.00	0.00	0.00
296.00	1.00	0.00	0.00	1.00	0.00	0.00	0.00	0.00	0.00	0.00
297.00	1.00	0.00	1.00	0.00	0.00	0.00	0.00	0.00	0.00	0.00
298.00	1.00	0.00	1.00	0.00	0.00	0.00	0.00	0.00	0.00	0.00
299.00	1.00	0.00	1.00	0.00	0.00	0.00	0.00	0.00	0.00	0.00
300.00	1.00	0.00	1.00	0.00	0.00	0.00	0.00	0.00	0.00	0.00

301.00	0.00	1.00	0.00	0.00	0.00	0.00	1.00	0.00	0.00	0.00
302.00	0.00	1.00	0.00	0.00	0.00	0.00	1.00	0.00	0.00	0.00
303.00	1.00	0.00	1.00	0.00	0.00	0.00	0.00	0.00	0.00	0.00
304.00	0.00	1.00	0.00	0.00	0.00	0.00	1.00	0.00	0.00	0.00
305.00	0.00	1.00	0.00	0.00	0.00	0.00	1.00	0.00	0.00	0.00
306.00	0.00	1.00	0.00	0.00	0.00	0.00	1.00	0.00	0.00	0.00
307.00	1.00	0.00	1.00	0.00	0.00	0.00	0.00	0.00	0.00	0.00
308.00	1.00	0.00	1.00	0.00	0.00	0.00	0.00	0.00	0.00	0.00
309.00	1.00	0.00	1.00	0.00	0.00	0.00	0.00	0.00	0.00	0.00
310.00	1.00	0.00	0.00	1.00	0.00	0.00	0.00	0.00	0.00	0.00
311.00	0.00	1.00	0.00	0.00	0.00	0.00	1.00	0.00	0.00	0.00
312.00	999.00	999.00	999.00	999.00	999.00	999.00	999.00	999.00	999.00	999.00
313.00	999.00	999.00	999.00	999.00	999.00	999.00	999.00	999.00	999.00	999.00
314.00	1.00	0.00	0.00	1.00	0.00	0.00	0.00	0.00	0.00	0.00
315.00	1.00	0.00	0.00	0.00	1.00	0.00	0.00	0.00	0.00	0.00
316.00	1.00	0.00	0.00	0.00	1.00	0.00	0.00	0.00	0.00	0.00
317.00	1.00	0.00	0.00	0.00	1.00	0.00	0.00	0.00	0.00	0.00
318.00	1.00	0.00	0.00	0.00	1.00	0.00	0.00	0.00	0.00	0.00
319.00	1.00	0.00	0.00	0.00	1.00	0.00	0.00	0.00	0.00	0.00
320.00	1.00	0.00	0.00	0.00	1.00	0.00	0.00	0.00	0.00	0.00
321.00	1.00	0.00	0.00	0.00	1.00	0.00	0.00	0.00	0.00	0.00
322.00	1.00	0.00	0.00	0.00	1.00	0.00	0.00	0.00	0.00	0.00
323.00	1.00	0.00	0.00	0.00	1.00	0.00	0.00	0.00	0.00	0.00
324.00	1.00	0.00	0.00	0.00	1.00	0.00	0.00	0.00	0.00	0.00
325.00	1.00	0.00	0.00	0.00	1.00	0.00	0.00	0.00	0.00	0.00
326.00	1.00	0.00	0.00	0.00	1.00	0.00	0.00	0.00	0.00	0.00
327.00	1.00	0.00	0.00	0.00	1.00	0.00	0.00	0.00	0.00	0.00
328.00	1.00	0.00	0.00	0.00	1.00	0.00	0.00	0.00	0.00	0.00
329.00	1.00	0.00	0.00	0.00	1.00	0.00	0.00	0.00	0.00	0.00
330.00	1.00	0.00	0.00	0.00	1.00	0.00	0.00	0.00	0.00	0.00
331.00	1.00	0.00	0.00	0.00	1.00	0.00	0.00	0.00	0.00	0.00
332.00	1.00	0.00	0.00	0.00	1.00	0.00	0.00	0.00	0.00	0.00

333.00	1.00	0.00	0.00	0.00	1.00	0.00	0.00	0.00	0.00	0.00
334.00	1.00	0.00	0.00	0.00	1.00	0.00	0.00	0.00	0.00	0.00
335.00	1.00	0.00	0.00	0.00	1.00	0.00	0.00	0.00	0.00	0.00
336.00	0.00	1.00	0.00	0.00	0.00	0.00	0.00	1.00	0.00	0.00
337.00	0.00	1.00	0.00	0.00	0.00	0.00	0.00	1.00	0.00	0.00
338.00	0.00	1.00	0.00	0.00	0.00	0.00	0.00	1.00	0.00	0.00
339.00	0.00	1.00	0.00	0.00	0.00	0.00	0.00	1.00	0.00	0.00
340.00	0.00	1.00	0.00	0.00	0.00	0.00	0.00	1.00	0.00	0.00
341.00	0.00	1.00	0.00	0.00	0.00	0.00	0.00	1.00	0.00	0.00
342.00	0.00	1.00	0.00	0.00	0.00	0.00	0.00	1.00	0.00	0.00
343.00	0.00	1.00	0.00	0.00	0.00	0.00	0.00	1.00	0.00	0.00
344.00	1.00	0.00	0.00	1.00	0.00	0.00	0.00	0.00	0.00	0.00
345.00	1.00	0.00	0.00	1.00	0.00	0.00	0.00	0.00	0.00	0.00
346.00	1.00	0.00	0.00	0.00	1.00	0.00	0.00	0.00	0.00	0.00
347.00	1.00	0.00	0.00	0.00	1.00	0.00	0.00	0.00	0.00	0.00
348.00	0.00	1.00	0.00	0.00	0.00	0.00	0.00	1.00	0.00	0.00
349.00	0.00	1.00	0.00	0.00	0.00	0.00	0.00	1.00	0.00	0.00
350.00	1.00	0.00	0.00	1.00	0.00	0.00	0.00	0.00	0.00	0.00
351.00	1.00	0.00	0.00	1.00	0.00	0.00	0.00	0.00	0.00	0.00
352.00	1.00	0.00	1.00	0.00	0.00	0.00	0.00	0.00	0.00	0.00
353.00	1.00	0.00	1.00	0.00	0.00	0.00	0.00	0.00	0.00	0.00
354.00	1.00	0.00	1.00	0.00	0.00	0.00	0.00	0.00	0.00	0.00
355.00	1.00	0.00	1.00	0.00	0.00	0.00	0.00	0.00	0.00	0.00
356.00	1.00	0.00	1.00	0.00	0.00	0.00	0.00	0.00	0.00	0.00
357.00	0.00	1.00	0.00	0.00	0.00	0.00	1.00	0.00	0.00	0.00
358.00	0.00	1.00	0.00	0.00	0.00	0.00	1.00	0.00	0.00	0.00
359.00	0.00	1.00	0.00	0.00	0.00	0.00	1.00	0.00	0.00	0.00
360.00	999.00	999.00	999.00	999.00	999.00	999.00	999.00	999.00	999.00	999.00
361.00	1.00	0.00	1.00	0.00	0.00	0.00	0.00	0.00	0.00	0.00
362.00	1.00	0.00	1.00	0.00	0.00	0.00	0.00	0.00	0.00	0.00
363.00	999.00	999.00	999.00	999.00	999.00	999.00	999.00	999.00	999.00	999.00
364.00	999.00	999.00	999.00	999.00	999.00	999.00	999.00	999.00	999.00	999.00

365.00	1.00	0.00	0.00	0.00	1.00	0.00	0.00	0.00	0.00	0.00
366.00	1.00	0.00	0.00	0.00	1.00	0.00	0.00	0.00	0.00	0.00
367.00	1.00	0.00	0.00	0.00	1.00	0.00	0.00	0.00	0.00	0.00
368.00	0.00	1.00	0.00	0.00	0.00	0.00	0.00	1.00	0.00	0.00
369.00	1.00	0.00	1.00	0.00	0.00	0.00	0.00	0.00	0.00	0.00
370.00	1.00	0.00	1.00	0.00	0.00	0.00	0.00	0.00	0.00	0.00
371.00	1.00	0.00	0.00	0.00	1.00	0.00	0.00	0.00	0.00	0.00
372.00	1.00	0.00	1.00	0.00	0.00	0.00	0.00	0.00	0.00	0.00
373.00	999.00	999.00	999.00	999.00	999.00	999.00	999.00	999.00	999.00	999.00
374.00	999.00	999.00	999.00	999.00	999.00	999.00	999.00	999.00	999.00	999.00
375.00	999.00	999.00	999.00	999.00	999.00	999.00	999.00	999.00	999.00	999.00
376.00	1.00	0.00	0.00	0.00	1.00	0.00	0.00	0.00	0.00	0.00
377.00	1.00	0.00	1.00	0.00	0.00	0.00	0.00	0.00	0.00	0.00
378.00	999.00	999.00	999.00	999.00	999.00	999.00	999.00	999.00	999.00	999.00
379.00	1.00	0.00	1.00	0.00	0.00	0.00	0.00	0.00	0.00	0.00
380.00	0.00	1.00	0.00	0.00	0.00	0.00	0.00	1.00	0.00	0.00
381.00	1.00	0.00	1.00	0.00	0.00	0.00	0.00	0.00	0.00	0.00
382.00	1.00	0.00	0.00	0.00	0.00	1.00	0.00	0.00	0.00	0.00
383.00	0.00	2.00	0.00	0.00	0.00	0.00	2.00	0.00	0.00	0.00
384.00	999.00	999.00	999.00	999.00	999.00	999.00	999.00	999.00	999.00	999.00
385.00	999.00	999.00	999.00	999.00	999.00	999.00	999.00	999.00	999.00	999.00
386.00	999.00	999.00	999.00	999.00	999.00	999.00	999.00	999.00	999.00	999.00
387.00	999.00	999.00	999.00	999.00	999.00	999.00	999.00	999.00	999.00	999.00
388.00	999.00	999.00	999.00	999.00	999.00	999.00	999.00	999.00	999.00	999.00
389.00	1.00	0.00	0.00	0.00	0.00	1.00	0.00	0.00	0.00	0.00
390.00	1.00	0.00	0.00	0.00	0.00	1.00	0.00	0.00	0.00	0.00
391.00	1.00	1.00	0.00	1.00	0.00	0.00	0.00	1.00	0.00	0.00
392.00	999.00	999.00	999.00	999.00	999.00	999.00	999.00	999.00	999.00	999.00
393.00	999.00	999.00	999.00	999.00	999.00	999.00	999.00	999.00	999.00	999.00
394.00	999.00	999.00	999.00	999.00	999.00	999.00	999.00	999.00	999.00	999.00
395.00	999.00	999.00	999.00	999.00	999.00	999.00	999.00	999.00	999.00	999.00
396.00	999.00	999.00	999.00	999.00	999.00	999.00	999.00	999.00	999.00	999.00

397.00	999.00	999.00	999.00	999.00	999.00	999.00	999.00	999.00	999.00	999.00
398.00	999.00	999.00	999.00	999.00	999.00	999.00	999.00	999.00	999.00	999.00
399.00	999.00	999.00	999.00	999.00	999.00	999.00	999.00	999.00	999.00	999.00
400.00	999.00	999.00	999.00	999.00	999.00	999.00	999.00	999.00	999.00	999.00
401.00	999.00	999.00	999.00	999.00	999.00	999.00	999.00	999.00	999.00	999.00
402.00	999.00	999.00	999.00	999.00	999.00	999.00	999.00	999.00	999.00	999.00
403.00	999.00	999.00	999.00	999.00	999.00	999.00	999.00	999.00	999.00	999.00
404.00	999.00	999.00	999.00	999.00	999.00	999.00	999.00	999.00	999.00	999.00
405.00	999.00	999.00	999.00	999.00	999.00	999.00	999.00	999.00	999.00	999.00
406.00	999.00	999.00	999.00	999.00	999.00	999.00	999.00	999.00	999.00	999.00
407.00	999.00	999.00	999.00	999.00	999.00	999.00	999.00	999.00	999.00	999.00
408.00	999.00	999.00	999.00	999.00	999.00	999.00	999.00	999.00	999.00	999.00
409.00	999.00	999.00	999.00	999.00	999.00	999.00	999.00	999.00	999.00	999.00
410.00	999.00	999.00	999.00	999.00	999.00	999.00	999.00	999.00	999.00	999.00
411.00	999.00	999.00	999.00	999.00	999.00	999.00	999.00	999.00	999.00	999.00
412.00	999.00	999.00	999.00	999.00	999.00	999.00	999.00	999.00	999.00	999.00
413.00	999.00	999.00	999.00	999.00	999.00	999.00	999.00	999.00	999.00	999.00
414.00	999.00	999.00	999.00	999.00	999.00	999.00	999.00	999.00	999.00	999.00
415.00	999.00	999.00	999.00	999.00	999.00	999.00	999.00	999.00	999.00	999.00
416.00	999.00	999.00	999.00	999.00	999.00	999.00	999.00	999.00	999.00	999.00
417.00	999.00	999.00	999.00	999.00	999.00	999.00	999.00	999.00	999.00	999.00
418.00	999.00	999.00	999.00	999.00	999.00	999.00	999.00	999.00	999.00	999.00
419.00	999.00	999.00	999.00	999.00	999.00	999.00	999.00	999.00	999.00	999.00
420.00	999.00	999.00	999.00	999.00	999.00	999.00	999.00	999.00	999.00	999.00
421.00	999.00	999.00	999.00	999.00	999.00	999.00	999.00	999.00	999.00	999.00
422.00	999.00	999.00	999.00	999.00	999.00	999.00	999.00	999.00	999.00	999.00
423.00	999.00	999.00	999.00	999.00	999.00	999.00	999.00	999.00	999.00	999.00
424.00	999.00	999.00	999.00	999.00	999.00	999.00	999.00	999.00	999.00	999.00
425.00	999.00	999.00	999.00	999.00	999.00	999.00	999.00	999.00	999.00	999.00
426.00	999.00	999.00	999.00	999.00	999.00	999.00	999.00	999.00	999.00	999.00
427.00	999.00	999.00	999.00	999.00	999.00	999.00	999.00	999.00	999.00	999.00
428.00	999.00	999.00	999.00	999.00	999.00	999.00	999.00	999.00	999.00	999.00

429.00	999.00	999.00	999.00	999.00	999.00	999.00	999.00	999.00	999.00	999.00
430.00	999.00	999.00	999.00	999.00	999.00	999.00	999.00	999.00	999.00	999.00
431.00	999.00	999.00	999.00	999.00	999.00	999.00	999.00	999.00	999.00	999.00
432.00	999.00	999.00	999.00	999.00	999.00	999.00	999.00	999.00	999.00	999.00
433.00	999.00	999.00	999.00	999.00	999.00	999.00	999.00	999.00	999.00	999.00
434.00	999.00	999.00	999.00	999.00	999.00	999.00	999.00	999.00	999.00	999.00
435.00	1.00	0.00	0.00	0.00	0.00	1.00	0.00	0.00	0.00	0.00
436.00	1.00	0.00	0.00	0.00	0.00	1.00	0.00	0.00	0.00	0.00
437.00	999.00	999.00	999.00	999.00	999.00	999.00	999.00	999.00	999.00	999.00
438.00	0.00	1.00	0.00	0.00	0.00	0.00	1.00	0.00	0.00	0.00
439.00	1.00	0.00	0.00	0.00	0.00	1.00	0.00	0.00	0.00	0.00
440.00	999.00	999.00	999.00	999.00	999.00	999.00	999.00	999.00	999.00	999.00
441.00	0.00	1.00	0.00	0.00	0.00	0.00	1.00	0.00	0.00	0.00
442.00	0.00	2.00	0.00	0.00	0.00	0.00	1.00	0.00	0.00	1.00
443.00	2.00	0.00	0.00	2.00	0.00	0.00	0.00	0.00	0.00	0.00
444.00	1.00	0.00	1.00	0.00	0.00	0.00	0.00	0.00	0.00	0.00
445.00	1.00	0.00	1.00	0.00	0.00	0.00	0.00	0.00	0.00	0.00
446.00	1.00	0.00	1.00	0.00	0.00	0.00	0.00	0.00	0.00	0.00
447.00	1.00	0.00	1.00	0.00	0.00	0.00	0.00	0.00	0.00	0.00
448.00	1.00	0.00	1.00	0.00	0.00	0.00	0.00	0.00	0.00	0.00
449.00	0.00	1.00	0.00	0.00	0.00	0.00	1.00	0.00	0.00	0.00
450.00	0.00	1.00	0.00	0.00	0.00	0.00	1.00	0.00	0.00	0.00
451.00	0.00	2.00	0.00	0.00	0.00	0.00	2.00	0.00	0.00	0.00
452.00	1.00	1.00	1.00	0.00	0.00	0.00	1.00	0.00	0.00	0.00
453.00	0.00	1.00	0.00	0.00	0.00	0.00	1.00	0.00	0.00	0.00
454.00	0.00	1.00	0.00	0.00	0.00	0.00	1.00	0.00	0.00	0.00
455.00	1.00	1.00	0.00	0.00	0.00	1.00	0.00	0.00	0.00	1.00
456.00	1.00	1.00	0.00	0.00	0.00	1.00	0.00	0.00	0.00	1.00
457.00	1.00	0.00	1.00	0.00	0.00	0.00	0.00	0.00	0.00	0.00
458.00	1.00	0.00	0.00	0.00	0.00	1.00	0.00	0.00	0.00	0.00
459.00	1.00	0.00	1.00	0.00	0.00	0.00	0.00	0.00	0.00	0.00
460.00	1.00	0.00	1.00	0.00	0.00	0.00	0.00	0.00	0.00	0.00

461.00	2.00	0.00	1.00	0.00	0.00	1.00	0.00	0.00	0.00	0.00
462.00	999.00	999.00	999.00	999.00	999.00	999.00	999.00	999.00	999.00	999.00
463.00	1.00	0.00	1.00	0.00	0.00	0.00	0.00	0.00	0.00	0.00
464.00	999.00	999.00	999.00	999.00	999.00	999.00	999.00	999.00	999.00	999.00
465.00	2.00	0.00	1.00	0.00	0.00	1.00	0.00	0.00	0.00	0.00
466.00	1.00	0.00	0.00	0.00	0.00	1.00	0.00	0.00	0.00	0.00
467.00	0.00	1.00	0.00	0.00	0.00	0.00	1.00	0.00	0.00	0.00
468.00	0.00	1.00	0.00	0.00	0.00	0.00	1.00	0.00	0.00	0.00
469.00	0.00	1.00	0.00	0.00	0.00	0.00	1.00	0.00	0.00	0.00
470.00	999.00	999.00	999.00	999.00	999.00	999.00	999.00	999.00	999.00	999.00
471.00	999.00	999.00	999.00	999.00	999.00	999.00	999.00	999.00	999.00	999.00
472.00	999.00	999.00	999.00	999.00	999.00	999.00	999.00	999.00	999.00	999.00
473.00	999.00	999.00	999.00	999.00	999.00	999.00	999.00	999.00	999.00	999.00
474.00	999.00	999.00	999.00	999.00	999.00	999.00	999.00	999.00	999.00	999.00
475.00	999.00	999.00	999.00	999.00	999.00	999.00	999.00	999.00	999.00	999.00
476.00	999.00	999.00	999.00	999.00	999.00	999.00	999.00	999.00	999.00	999.00
477.00	999.00	999.00	999.00	999.00	999.00	999.00	999.00	999.00	999.00	999.00
478.00	999.00	999.00	999.00	999.00	999.00	999.00	999.00	999.00	999.00	999.00
479.00	999.00	999.00	999.00	999.00	999.00	999.00	999.00	999.00	999.00	999.00
480.00	999.00	999.00	999.00	999.00	999.00	999.00	999.00	999.00	999.00	999.00
481.00	999.00	999.00	999.00	999.00	999.00	999.00	999.00	999.00	999.00	999.00
482.00	999.00	999.00	999.00	999.00	999.00	999.00	999.00	999.00	999.00	999.00
483.00	999.00	999.00	999.00	999.00	999.00	999.00	999.00	999.00	999.00	999.00
484.00	1.00	1.00	1.00	0.00	0.00	0.00	1.00	0.00	0.00	0.00
485.00	1.00	1.00	1.00	0.00	0.00	0.00	1.00	0.00	0.00	0.00
486.00	999.00	999.00	999.00	999.00	999.00	999.00	999.00	999.00	999.00	999.00
487.00	999.00	999.00	999.00	999.00	999.00	999.00	999.00	999.00	999.00	999.00
488.00	0.00	1.00	0.00	0.00	0.00	0.00	1.00	0.00	0.00	0.00
489.00	0.00	1.00	0.00	0.00	0.00	0.00	1.00	0.00	0.00	0.00
490.00	0.00	1.00	0.00	0.00	0.00	0.00	1.00	0.00	0.00	0.00
491.00	0.00	1.00	0.00	0.00	0.00	0.00	1.00	0.00	0.00	0.00
492.00	0.00	1.00	0.00	0.00	0.00	0.00	0.00	0.00	0.00	1.00

493.00	0.00	1.00	0.00	0.00	0.00	0.00	0.00	0.00	0.00	1.00
494.00	1.00	0.00	1.00	0.00	0.00	0.00	0.00	0.00	0.00	0.00
495.00	1.00	0.00	0.00	0.00	0.00	1.00	0.00	0.00	0.00	0.00
496.00	0.00	3.00	0.00	0.00	0.00	0.00	1.00	0.00	2.00	0.00
497.00	1.00	1.00	1.00	0.00	0.00	0.00	1.00	0.00	0.00	0.00
498.00	0.00	1.00	0.00	0.00	0.00	0.00	1.00	0.00	0.00	0.00
499.00	1.00	1.00	0.00	0.00	0.00	1.00	1.00	0.00	0.00	0.00
500.00	0.00	1.00	0.00	0.00	0.00	0.00	1.00	0.00	0.00	0.00
501.00	0.00	1.00	0.00	0.00	0.00	0.00	0.00	1.00	0.00	0.00
502.00	1.00	0.00	1.00	0.00	0.00	0.00	0.00	0.00	0.00	0.00
503.00	1.00	0.00	1.00	0.00	0.00	0.00	0.00	0.00	0.00	0.00
504.00	1.00	0.00	1.00	0.00	0.00	0.00	0.00	0.00	0.00	0.00
505.00	1.00	0.00	1.00	0.00	0.00	0.00	0.00	0.00	0.00	0.00
506.00	1.00	0.00	0.00	1.00	0.00	0.00	0.00	0.00	0.00	0.00
507.00	0.00	1.00	0.00	0.00	0.00	0.00	1.00	0.00	0.00	0.00
508.00	1.00	0.00	1.00	0.00	0.00	0.00	0.00	0.00	0.00	0.00
509.00	0.00	2.00	0.00	0.00	0.00	0.00	2.00	0.00	0.00	0.00
510.00	1.00	0.00	0.00	0.00	0.00	1.00	0.00	0.00	0.00	0.00
511.00	1.00	0.00	1.00	0.00	0.00	0.00	0.00	0.00	0.00	0.00
512.00	0.00	1.00	0.00	0.00	0.00	0.00	1.00	0.00	0.00	0.00
513.00	0.00	1.00	0.00	0.00	0.00	0.00	1.00	0.00	0.00	0.00
514.00	1.00	0.00	1.00	0.00	0.00	0.00	0.00	0.00	0.00	0.00
515.00	999.00	999.00	999.00	999.00	999.00	999.00	999.00	999.00	999.00	999.00
516.00	1.00	0.00	1.00	0.00	0.00	0.00	0.00	0.00	0.00	0.00
517.00	1.00	0.00	0.00	0.00	0.00	1.00	0.00	0.00	0.00	0.00
518.00	999.00	999.00	999.00	999.00	999.00	999.00	999.00	999.00	999.00	999.00
519.00	1.00	0.00	1.00	0.00	0.00	0.00	0.00	0.00	0.00	0.00
520.00	1.00	0.00	1.00	0.00	0.00	0.00	0.00	0.00	0.00	0.00
521.00	1.00	0.00	1.00	0.00	0.00	0.00	0.00	0.00	0.00	0.00
522.00	1.00	0.00	1.00	0.00	0.00	0.00	0.00	0.00	0.00	0.00
523.00	1.00	0.00	1.00	0.00	0.00	0.00	0.00	0.00	0.00	0.00
524.00	1.00	0.00	1.00	0.00	0.00	0.00	0.00	0.00	0.00	0.00

525.00	2.00	0.00	1.00	1.00	0.00	0.00	0.00	0.00	0.00	0.00
526.00	1.00	0.00	1.00	0.00	0.00	0.00	0.00	0.00	0.00	0.00
527.00	0.00	1.00	0.00	0.00	0.00	0.00	0.00	1.00	0.00	0.00
528.00	1.00	0.00	1.00	0.00	0.00	0.00	0.00	0.00	0.00	0.00
529.00	1.00	0.00	1.00	0.00	0.00	0.00	0.00	0.00	0.00	0.00
530.00	1.00	0.00	1.00	0.00	0.00	0.00	0.00	0.00	0.00	0.00
531.00	1.00	0.00	1.00	0.00	0.00	0.00	0.00	0.00	0.00	0.00
532.00	1.00	0.00	1.00	0.00	0.00	0.00	0.00	0.00	0.00	0.00
533.00	1.00	0.00	1.00	0.00	0.00	0.00	0.00	0.00	0.00	0.00
534.00	0.00	1.00	0.00	0.00	0.00	0.00	1.00	0.00	0.00	0.00
535.00	1.00	0.00	0.00	0.00	1.00	0.00	0.00	0.00	0.00	0.00
536.00	2.00	0.00	1.00	1.00	0.00	0.00	0.00	0.00	0.00	0.00
537.00	1.00	0.00	1.00	0.00	0.00	0.00	0.00	0.00	0.00	0.00
538.00	0.00	1.00	0.00	0.00	0.00	0.00	1.00	0.00	0.00	0.00
539.00	1.00	0.00	1.00	0.00	0.00	0.00	0.00	0.00	0.00	0.00
540.00	2.00	0.00	1.00	0.00	0.00	1.00	0.00	0.00	0.00	0.00
541.00	1.00	0.00	1.00	0.00	0.00	0.00	0.00	0.00	0.00	0.00
542.00	1.00	0.00	1.00	0.00	0.00	0.00	0.00	0.00	0.00	0.00
543.00	1.00	0.00	1.00	0.00	0.00	0.00	0.00	0.00	0.00	0.00
544.00	0.00	1.00	0.00	0.00	0.00	0.00	1.00	0.00	0.00	0.00
545.00	999.00	999.00	999.00	999.00	999.00	999.00	999.00	999.00	999.00	999.00
